# Tuning the activity of iminosugars: novel *N*-alkylated deoxynojirimycin derivatives as strong BuChE inhibitors

**DOI:** 10.1080/14756366.2020.1847101

**Published:** 2020-11-23

**Authors:** Ana I. Ahuja-Casarín, Penélope Merino-Montiel, José Luis Vega-Baez, Sara Montiel-Smith, Miguel X. Fernandes, Irene Lagunes, Inés Maya, José M. Padrón, Óscar López, José G. Fernández-Bolaños

**Affiliations:** a Facultad de Ciencias Químicas, Ciudad Universitaria, Benemérita Universidad Autónoma de Puebla, Puebla, México; b BioLab, Instituto Universitario de Bio-Orgánica “Antonio González” (IUBO-AG), Universidad de La Laguna, La Laguna, Spain; c Departamento de Química Orgánica, Facultad de Química, Universidad de Sevilla, Seville, Spain

**Keywords:** Iminosugars, 1-DNJ, cholinesterase inhibitors, anti-Alzheimer’s agents, docking simulations

## Abstract

We have designed unprecedented cholinesterase inhibitors based on 1-deoxynojirimycin as potential anti-Alzheimer’s agents. Compounds are comprised of three key structural motifs: the iminosugar, for interaction with cholinesterase catalytic anionic site (CAS); a hydrocarbon tether with variable lengths, and a fragment derived from 2-phenylethanol for promoting interactions with peripheral anionic site (PAS). Title compounds exhibited good selectivity towards BuChE, strongly depending on the substitution pattern and the length of the tether. The lead compounds were found to be strong mixed inhibitors of BuChE (IC_50_ = 1.8 and 1.9 µM). The presumptive binding mode of the lead compound was analysed using molecular docking simulations, revealing H-bond interactions with the catalytic subsite (His438) and CAS (Trp82 and Glu197) and van der Waals interactions with PAS (Thr284, Pro285, Asn289). They also lacked significant antiproliferative activity against tumour and non-tumour cells at 100 µM, making them promising new agents for tackling Alzheimer’s disease through the cholinergic approach.

## Graphical Abstract

**Figure:**
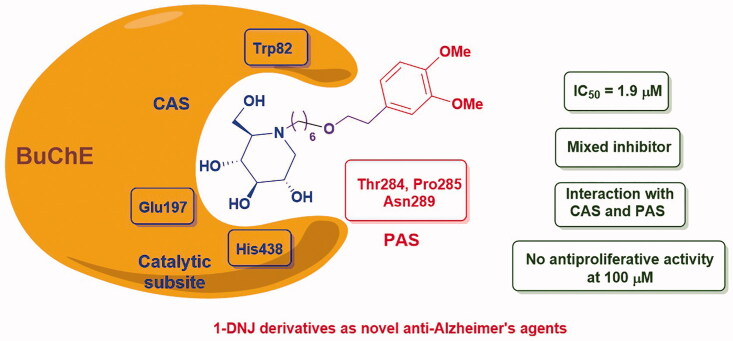

## Introduction

Imino- and azasugars, that is, carbohydrate mimetics where the endocyclic oxygen or a carbon atom, respectively, has been replaced by a nitrogen atom that has attracted great attention within Medicinal Chemistry since several decades ago^1^. The key structural motif of such glycomimetics is a protonable nitrogen atom, which might allow them to mimic transition states (in terms of charge and geometry)^2^ of the glycosidase-mediated hydrolysis of glycosidic bonds. Because of this, many imino- and azasugars behave as potent inhibitors of ubiquitous glycosidases and glycosyltransferases^1^, key enzymes in a plethora of biological processes where carbohydrates are involved, including metabolic pathways, cell wall formation, and recognition events. Numerous imino- and azasugars have shown relevant pharmacological activities^3^, such as antidiabetic^4^, anticancer^5^, antifungal^6^, antiprotozoal^7^ or antiviral agents^8^, among others. In this sense, Sirona Biochem Corp. has just announced^9^ the launching of an intense programme searching for new antivirals for combating the pandemic caused by COVID-19, in which iminosugars are one of the three categories in the study. This choice is strongly supported by the World Laureates Association Shanghai Centre. Some iminosugars have also been found to act as chaperones^10^ against lysosomal storage disorders, like Gaucher’s^11^ and Fabry’s^12^ diseases. Iminosugars have also been found to be effective against cystic fibrosis^13^, an autosomal recessive disease-causing severe multi-system organ damage, particularly to the respiratory system. Iminosugars can act as correctors of defective cystic fibrosis transmembrane conductance regulator (CFTR)^14^^,^^15^, an ABC transporter-class protein that controls the content of ion and water in epithelial cells, or alternatively they can reduce the inflammatory response of pathogens, like *P. aeruginosa*^16^. More recently, conjugation of iminosugars with sulphonamides resulted in the inhibition of relevant human carbonic anhydrases^17^. Among the vast number of iminosugars and related structures reported so far, 1-deoxynojirimycin (1-DNJ), a natural iminosugar isolated from mulberry leaves^18^, is the basis for the so-far two marketed drugs derived from iminosugars: Miglitol^®^ (*N*-hydroxyethyl-1-deoxynojirimycin)^19^ and Zavesca^®^ (*N*-butyl-1-deoxynojirimycin)^20^, used for tackling non-insulin-dependent diabetes, and Gaucher’s disease, respectively.

Nevertheless, a field that still remains relatively unexplored concerning iminosugars is Alzheimer’s disease. Alzheimer’s is a devastating neurodegenerative disease, considered nowadays the most prevalent form of dementia, counting for roughly 70% of total cases^21^. It causes severe impairment of cognitive functionality, neurodegeneration, and even Parkinsonian symptoms, leading the patient to a complete dependence even for accomplishing daily tasks^22^. According to the World Alzheimer Report 2019, roughly 50 million people have been diagnosed worldwide, and this number is expected to increase almost exponentially to 152 million people by 2050^23^. Another worrying aspect is that, although Alzheimer’s disease has been classically associated with elderly people, the average age of patients has decreased significantly^24^.

From a pathophysiological point of view, Alzheimer’s disease is one of the most complex diseases nowadays, whose multifactorial aetiology is not even completely understood. This hampers enormously finding a treatment. The most recognised hallmarks are^25^ the deposit of toxic amyloid plaques, neurofibrillary tangles (via hyperphosphorylation of tau proteins), and low levels of the neurotransmitter acetylcholine, which is associated with the cognitive decline.

Currently, there are four marketed drugs for ameliorating the symptoms of Alzheimer’s disease^26^ aiming at two therapeutic targets: the inhibitors of cholinesterases (acetylcholinesterase, AChE; and butyrylcholinesterase, BuChE) donepezil, rivastigmine and galantamine, which increase the levels of acetylcholine (*cholinergic hypothesis*), and the antagonist of *N*-methyl-d-aspartic receptors (NMDA) memantine, for regulating glutamate levels in brain cells.

In connection with iminosugars, Zavesca^®^ was recently found to reduce the amyloid plaque production in a cell model^27^. Compain and co-workers analysed the cholinesterase inhibition of a series of iminosugars with different substituents motifs and stereochemistry^28^. In our research group, we recently combined 1-DNJ with a selenoureido appendage to achieve strong β-glucosidase and AChE inhibitors^29^. To the best of our knowledge, these are the only examples of iminosugars exhibiting anti-Alzheimer’s properties.

Our main target herein has been the design of new potential AChE and/or BuChE inhibitors against Alzheimer’s disease using 1-DNJ as the core template. Rationalisation of their biological properties by analysing interactions with key enzymatic domains is also covered herein.

## Materials and methods

### General procedures

^1^H (300.1 MHz) and ^13^C (75.5 MHz) NMR spectra were recorded at 25 °C on a Bruker Avance 300 spectrometer using the deuterated solvent indicated in each case. ^1^H and ^13^C assignments were confirmed by 2D COSY and HSQC experiments. Mass spectra (ESI) were recorded on a Q Exactive mass spectrometer. TLCs were performed on aluminium pre-coated sheets (E. Merck Silica gel 60 F_254_); spots were visualised by UV light, and by charring with 10% vanillin in EtOH containing 1% of H_2_SO_4_, or with 10% H_2_SO_4_ in EtOH. Column chromatography was performed using E. Merck Silica Gel 60 (40–63 μm), using the eluent indicated in each case.

### Chemistry

#### General procedure for the preparation of compounds 7 and 8

To a solution of the corresponding 2-arylethanol derivatives **14a–g** (2.19 mmol) in DMF (5 ml), 60% NaH (525 mg, 13.14 mmol, 6.0 equiv.) was added and the resulting mixture was kept stirring at rt under an inert atmosphere for 30 min. Then, 1,6-dibromohexane or 1,5-dibromopentane was added (17.52 mmol, 8.0 equiv.), and stirring was kept for a further 5 h in the case of **7**, and 4 h in the case of **8**. The reaction was quenched by the careful addition of water at 0 °C. After that, the crude reaction mixture was diluted with brine (50 ml) and extracted with ethyl acetate (3 × 50 ml). The combined organic fractions were dried over Na_2_SO_4_, filtered and the filtrate was concentrated to dryness. The residue was purified by column chromatography, using the eluent indicated in each case to give compounds **7** and **8** as colourless oils (See Supplementary Material).

#### General procedure for the preparation of compounds 9 and 10

To a solution of per-*O*-benzylated 1-deoxynojirimycin **6** (250 mg, 0.48 mmol) in DMF (5 ml), K_2_CO_3_ (199 mg, 1.44 mmol, 3.0 equiv.) and *O*-alkylated derivatives **7** or **8** (1.19 mmol, 2.5 equiv.) were added. The corresponding mixture was kept stirring at 85 °C for 15 h. Then water was added, and products were portioned between brine (50 ml) and EtOAc (3 × 50 ml). The combined organic fractions were dried over Na_2_SO_4_, filtered and the filtrate was concentrated to dryness under reduced pressure. The residue was purified by column chromatography using the eluents indicated in each case, to give compounds **9** and **10** as colourless syrups (See Supplementary Material).

#### General procedure for the preparation of compounds 11 and 12

To a solution of benzylated derivative **9** or **10** (0.20 mmol) in a 1:1 CH_2_Cl_2_‒MeOH mixture (6 ml), AcOH (0.4 ml) and Pd(OH)_2_/C (150 mg) were added, and the corresponding mixture was hydrogenated for 24 h. After that, it was filtered over a Celite^®^ pad and washed with MeOH. The filtrate was concentrated to dryness and purified by column chromatography (190:10:1 → 140:60:1 CH_2_Cl_2_‒MeOH‒Et_3_N) in the case of **11**. For compounds **12**, no further purification was accomplished after filtration. Derivatives **11** and **12** were obtained as colourless syrups (See Supplementary Material).

### Enzymatic assays

For both families of enzymes used herein (glycosidases and cholinesterases), stock solutions of the inhibitors were prepared in DMSO; DMSO content was kept at 5% (V/V) for glycosidases and 1.25% (V/V) for cholinesterases. Enzymes were properly dissolved in water in a concentration in which the rate of the reaction when using [S] = 4 × *K*_M_ is in the range 0.12–0.15 Abs/min. In both kinds of enzymes, an initial screening at 100 µM inhibitor concentration was performed, using the following substrate concentrations: [S] = 0.25 mM for α-glucosidase (*Saccharomyces cerevisiae*), 4.0 mM for β-glucosidase (almonds), 0.60 mM for α-galactosidase (green coffee beans), 0.51 mM for β-galactosidase (*Escherichia coli*), 1.5 mM for β-galactosidase (*Aspergillus Oryzae*), 0.12 mM for AChE (*Electrophorus electricus*), 0.11 mM for BuChE (equine serum). When the percentage of inhibition was higher than 50%, the kinetic parameters and the inhibition constants were calculated using five different substrate concentrations ranging from ¼ *K*_M_ to 4 × *K*_M_, and 2–4 different inhibitor concentrations (affording roughly 20–70% inhibition). Each assay was run in duplicate.

Glycosidase assays were accomplished using the methodology reported by Bols and co-workers^30^. Each set of experiments is prepared in PS cuvettes using 0.1 M phosphate buffer (pH 6.8) and the corresponding *o*- or *p*-nitrophenyl glycopyranosides as substrates. Reactions were monitored at 25 °C by following the formation of the corresponding nitrophenolates at 400 nm (glucosidases and α-galactosidase) or 420 nm (β-galactosidases) over a time of 125 s.

For cholinesterases, minor modifications on Ellman’s assay^31^ were performed. The activity was measured in PS cuvettes containing 0.1 mM phosphate buffer (pH 8.0), 0.88 mM DTNB, and substrate (acetyl- and butyryl-thiocholine iodides for AChE and BuChE, respectively), The formation of the chromophore was monitored at 405 nm over a time of 125 s.

For strong inhibitors, the mode of inhibition was determined using the Cornish–Bowden method, which involves the use of two different plots: 1/v vs. [I] (Dixon plot) and [S]/v vs. [I]. For the calculation of kinetic parameters (*K*_M_, *V*_max_) a nonlinear regression analysis (least squares fit, GraphPad Prism 8.01) was used. Data are expressed as the mean ± SD.

### Docking simulations

Interactions of cholinesterases with the compounds were analysed by computational docking using MOE software (Chemical Computing Group). Crystallographic structures of human AChE and human BuChE was obtained from Protein Data Bank (PDB code 4EY6 and 4AQD, respectively). Protein structures was energetically minimised using Amber10 force field with EHT parameters for small molecules, R-field solvation model, dielectric constant of 1 for the protein interior and 80 for the exterior. Ligand structure was drawn in MOE software, and its energy was minimised with the above parameters using as stop criterion an RMS gradient lower than 0.01 kcal/mol/Å. For the docking calculations: in the placement stage we used the Triangle Matcher algorithm with the London dG scoring scheme. In the refinement stage, we kept the receptor rigid and used the GBVI/WSA dG scoring scheme. 2D diagrams were obtained from MOE software and 3D illustrations were obtained using Pymol software.

### Antiproliferative assays

We selected the human neuroblastoma cell line SH-SY5Y and the fibroblast cell line BJ-hTERT to evaluate the antiproliferative activity of the compounds. The tests were performed in 96-well plates using the SRB assay^32^ with the following specifications. Cell seeding densities were 5000 cells/well for SH-SY5Y and 7000 cells/well for BJ-hTERT. Drug incubation times were 48 h. The optical density of each well was measured at 530 (primary) and 620 (secondary) nm. The antiproliferative activity expressed as 50% growth inhibition (GI_50_), was calculated according to NCI formulas^33^.

## Results and discussion

### Chemistry

In terms of inhibition, AChE contains two key regions^34^: the catalytic anionic site (CAS), which enables cation–π interactions with the quaternary ammonium fragment of acetylcholine, the natural substrate of the enzyme, and the peripheral anionic site (PAS), enriched with aromatic amino acid residues, and located roughly 15 Å from CAS through a narrow gorge, capable of establishing interactions with planar and aromatic residues. It is important to mention that PAS is also involved in the initial step of β-amyloid aggregation^35^. Accordingly, dual inhibitors establishing favourable interactions with both, CAS and PAS, can be not only strong inhibitors, but they can also contribute to ameliorate the formation of neurotoxic amyloid plaques.

**Figure 1.**
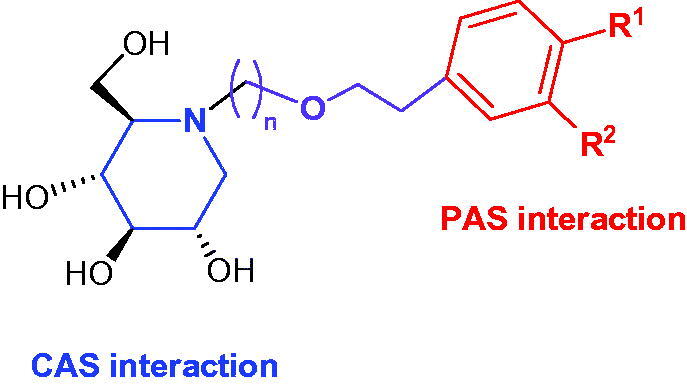
General structure of 1-DNJ derivatives as potential cholinesterase inhibitors designed on this work.

Inspired by the structure of AChE, we propose herein the preparation of a series of iminosugars-based cholinesterase inhibitors. The general structure for such derivatives is depicted in Figure 1, with three key structural motifs: the iminosugar residue, an alkoxy tether, and an aromatic motif with different substitution patterns. Oxygenated *N*-alkyl iminosugars have been found to reduce cytotoxicity when evaluated as antiviral agents^36^, thus diminishing side-effects. In particular, we envisioned the possibility of using 1-DNJ as the key core.

On the one hand, *N*-alkyl-DNJ derivatives could be partially protonated at physiological pH, thus mimicking the ammonium cation of acetylcholine, and enabling favourable interactions within the CAS. Although it has been reported that the presence of aromatic substituents connected to iminosugars through a short alkyl tether (2–3 carbon atoms) leads to p*K*a of 6.0^37^ or below 6.0^38^, because of the electron-withdrawing effect of the aryl moiety, compounds prepared herein, with longer alkyl fragments are expected to tentatively exhibit higher p*K*a values, probably in between for those reported for *N*-nonyl-DNJ and *N*-butyl-DNJ (6.7 and 7.1, respectively)^39^. On the other hand, as aforementioned, 1-DNJ has positive effects against amyloidogenesis. Moreover, 1-DNJ has recently been found^40^ to ameliorate stable angina pectoris in patients with coronary heart disease by reducing inflammatory responses and by increasing the antioxidant machinery. Taking into consideration that profound oxidative stress and neuroinflammation are common features of Alzheimer’s disease^41^, the use of 1-DNJ as the pharmacophore might also provide activity against other targets of this multifactorial disease. Furthermore, different substitution patterns on the aromatic appendage might modulate the interactions within the PAS region.

**Scheme 1.**
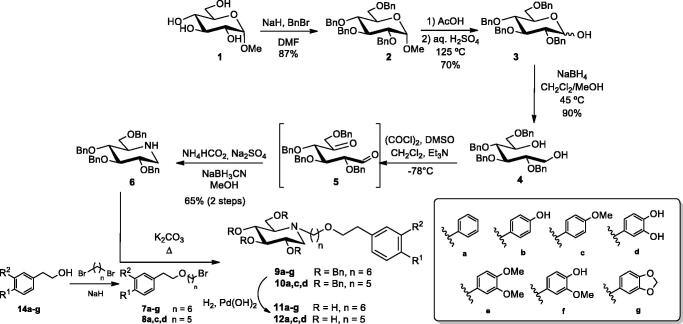
General procedure for the preparation of *N*-alkylated derivatives **11** and **12**.

For accessing compounds **11** and **12**, depicted in Scheme 1, the key intermediate is per-*O*-benzylated 1-DNJ **6**, which was accessed in a 5-step linear pathway (36% overall yield), following the procedure reported in the literature^42^, starting from commercially-available methyl α-d-glucopyranoside **1** (Scheme 1). Such procedure involves fully *O*-protection, acidic hydrolysis of the glycoside moiety, reduction of the masked aldehyde of **3**, Swern oxidation^43^, and fast reductive amination of transient unstable dicarbonyl compound **5**; the last step consists of a double reductive amination (DRA), a cascade reaction that provides a straightforward methodology for accessing polyhydroxylated piperidines^44^. Base-promoted *N*-alkylation with bromoderivatives **7** and **8**, and deprotection by hydrogenolysis, furnished compounds **11** and **12**, respectively, with the substitution pattern depicted in Scheme 1. We hypothesised that the presence of one or two methoxy groups could resemble the dimethoxy arene-based fragment of the indanone moiety in donepezil, and thus, might enable interaction with the PAS region of cholinesterases. Moreover, the use of a *o*-dihydroxyphenyl ring (catechol moiety) could afford relevant antioxidant properties^45^, as well as the capacity for chelating metal ions (Zn, Cu, Fe are present at toxic levels in the brain of Alzheimer’s patients)^46^.

To avoid side-reactions, those starting materials bearing free phenolic hydroxyl groups (natural tyrosol **13b**, hydroxytyrosol **13d** and homovanillic alcohol **13f**) were protected using a chemoselective benzylation under mild basic conditions (See Supporting Material, compounds **14b**, **d**, and **f**). Moreover, for the scaffold incorporating a methylidene motif, commercially-available 2–(3′,4′-methylenedioxyphenyl)acetic acid **15** was reduced (See Supporting Material) under mild conditions, using a combination of NaBH_4_ and I_2_^47^. It has been reported^48^ that such conditions lead to the *in situ* generations of borane, which reduces efficiently the carboxyl group to a primary alcohol. Such treatment furnished **14g** in a quantitative yield after chromatographic purification.

Mono-alkylation of 2-arylethanol derivatives **14a–g** was accomplished using an excess of 1,6-dibromohexane under basic conditions (NaH) in general from excellent to quantitative yields (Scheme 1) to give **7**, **8**. Alkylation on the endocyclic nitrogen atom of **6** (Scheme 1) was more complicated than previously anticipated. Nucleophilic displacement of **6** on the terminal bromine atom of **7a–g** accomplished derivatives **9a–g** in moderate yields (46‒68%). Final deprotection using standard hydrogenolysis conditions furnished derivatives **11a–g** in moderate to quantitative yields.

To analyse the influence of the linkage length on the biological properties, homologous **12a**, **c**, and **d** were also prepared as representative examples (Scheme 1). Noteworthy, extraordinary differences in activity towards cholinesterases were found by our group upon assessment of tacrine-derived homo- and heterodimers as anti-Alzheimer’s agents by small variations in the tether length^49^. Again, the limiting step in terms of yields was the *N*-alkylation of *O*-protected 1-DNJ 6. Analogously, previous attempts^50^ to alkylate the same position on azafagomine, a cyclic hydrazine analogue of 1-DNJ *via* nucleophilic substitutions failed. This was attributed to the high sterical hindrance exerted by the bulky exocyclic benzyloxymethyl moiety, in its major *gt* conformation, that occluded the entry of the reagents.

### Biological assays

The ten 1-DNJ derivatives prepared herein (**11a–g**, **12a**, **c**, and **d**) were subjected to inhibition assays on two families of enzymes: glycosidases and cholinesterases. In both cases, an initial screening was performed at 100 µM inhibitor concentration. For those compounds exhibiting a percentage of inhibition higher than 50% under such conditions, the kinetic parameters (*K*_M,app_ and *V*_max,app_) were obtained, that were used for calculating the inhibition constants (*K*_i_’s). The mode of inhibition was also established for the most potent compounds, using the Cornish–Bowden method^51^.

#### Glycosidase and cholinesterase inhibition studies

As aforementioned, glycosidases play a pivotal role in controlling not only the metabolic pathways where carbohydrates are present but also many other significant biochemical events, like cell-to-cell communication or recognition processes. Herein, a panel of five commercially-available glycosidases have been tested, as models of carbohydrate-mediated diseases: α-glucosidase (from *Saccharomyces cerevisiae*), β-glucosidase (from almonds), α-galactosidase (from green coffee beans) and β-galactosidase (from *A. oryzae* and from *E. coli*). Deficiencies in such enzymes are associated with diabetes, Gaucher’s disease, Fabry’s disease, and gangliosidosis, respectively^3^.

Assessment of the inhibitory capacity against glycosidases was accomplished using the methodology reported by Bols and co-workers^30^. *p*-Nitrophenyl glycosides (*o-* for β-galactosidases) were used as model substrates, and the release of the corresponding *p*-nitrophenolates at pH 6.8 was monitored spectrophotometrically (*λ*_max_ = 400 or 420 nm for *p*- or *o*-nitrophenolates, respectively).

Selected results are shown in Table 1, where the IC_50_ and inhibition constants values, together with the mode of inhibition are depicted. 1-DNJ is a good inhibitor of α-galactosidase and moderate of α-glucosidase (IC_50_ = 16 and 35 µM, respectively), and a weak inhibitor of β-glucosidase (IC_50_ = 71 µM). However, compounds **11** and **12** lacked significant activity against α-glucosidase and α/β-galactosidases (Table 1 and Table S1), and regarding β-glucosidase, compounds **11** ranged from good (**11b**, **d**, and **e**, IC_50_: 12–15 µM) to strong competitive inhibitors (**11a**, **c**, **f**, and **g**, IC_50_: 4.6–8.4 µM). This means a complete reversal of selectivity towards β-glucosidase, with a 15-fold increased activity compared to natural 1-DNJ, suggesting that the more lipophilic substituents favour interaction with β-glucosidase. The same behaviour was found upon alkylation on *N*-2 position of azafagomine^50^. Interestingly, a decrease in the tether length (compounds **12**) was found to be detrimental for the inhibition of β-glucosidase (e.g. 4‒6-fold for **12a**, **c**), showing the great influence of such structural motif.

**Table 1.** Enzyme inhibition for selected compounds **11a–g** (IC_50_, *K*_i_ in µM).


Compound	Enzyme			
	α-Glucosidase	β-Glucosidase	AChE	BuChE
**11a** R^1^=R^2^=H	IC_50_ = 126 *K*_ia_ = 93 ± 32 (Competitive)	**IC_50_ = 4.6** ***K*_ia_ = 6.5 ± 0.1** (Competitive)	IC_50_ >100	IC_50_ >100
**11b** R^1^=OH, R^2^=H	IC_50_ >100	IC_50_ = 12 *K*_ia_ = 17 ± 5 (Competitive)	IC_50_ >100	IC_50_ >100
**11c** R^1^=OMe, R^2^=H	IC_50_ >100	**IC_50_ = 6.0** ***K*_ia_ = 4.5 ± 0.7** (Competitive)	IC_50_ >100	IC_50_ >100
**11d** R^1^=R^2^=OH	IC_50_ >100	IC_50_ = 15 *K*_ia_ = 11 ± 4 (Competitive)	IC_50_ >100	IC_50_= 76
**11e** R^1^=R^2^=OMe	IC_50_ >100	IC_50_ = 14 *K*_ia_ = 15 ± 3 (Competitive)	**IC_50_= 5.8** ***K*_ia_ = 9.3 ± 1.2** ***K*_ib_ = 5.2 ± 0.5** (Mixed)	**IC_50_ = 1.9** ***K*_ia_ = 1.4 ± 0.4** ***K*_ib_ = 4.5 ± 1.4** (Mixed)
**11f** R^1^=OH, R^2^=OMe	IC_50_ >100	**IC_50_ = 8.2** ***K*_ia_ = 9.6 ± 0.8** (Competitive)	**IC_50_= 7.3** *K*_ia_ = *K*_ib_ = 17 ± 5 (Non-competitive)	**IC_50_ = 1.8** ***K*_ia_ = 1.5 ± 0.2** ***K*_ib_ = 5.0 ± 2.0** (Mixed)
**11g** R^1^,R^2^=OCH_2_O	IC_50_ >100	**IC_50_= 8.4** ***K*_ia_ = 9.0 ± 3.0** (Competitive)	IC_50_ = 48 *K*_ia_= 56 ± 10 *K*_ib_= 43 ± 14 (Mixed)	**IC_50_ = 7.3** ***K*_ia_= *K*_ib_ =9.2 ± 1.9** (Non-competitive)
**1-DNJ**	IC_50_ = 35	IC_50_ = 71	IC_50_ >100	IC_50_ = 10 *K*_ia_ = *K*_ib_ = 16 ± 3 (Non-competitive)

Derivatives **11** and **12** were tested also against AChE (*Electrophorus electricus*) and BuChE (equine serum), as recognised models for human cholinesterases due to their close structural resemblance^52^. For that purpose, Ellman’s colorimetric assay was used; this is an indirect test in which acetyl and butyryl thiocholine iodides are used as model substrates, which upon interaction with the enzyme release thiocholine, which in turn reacts with the chromogen reagent 5,5′-dithiobis(2-nitrobenzoic acid (DTNB) to give 2-nitro-5-mercaptobenzoate (at pH 8.0). The monitoring of the latter at 405 nm furnishes the kinetic parameters.

Interesting structure-activity relationships can be extracted also from activity data (Table 1). Regarding AChE, clearly, the presence of a dimethoxyphenyl moiety furnishes the most potent compound (**11e**, IC_50,_ and *K*i’s in the low micromolar range, mixed inhibitor), as was initially hypothesised.

However, what is more, interesting about the anticholinergic activity of these compounds is that they show a clear preference towards BuChE. Thus, relatively lipophilic disubstitution (**11e–g**) affords the strongest compounds in the series, within the low micromolar range (IC_50_ 1.8–7.3 µM) (di-OMe≈OMe/OH > OCH_2_O).

An issue that must be addressed is that BuChE has a more prominent role than AChE in elderly people and in more advanced stages of Alzheimer’s disease^53^. Moreover, BuChE has also been reported to participate in amyloid plaques and neurofibrillary tangles-mediated neurodegeneration^54^. This means that compounds **11e–g** could be useful not only for re-establishing the cognitive functionality of the patients (by increasing the levels of cholinesterase) but also for retarding or ameliorating neurotoxicity associated with amyloidogenesis in advanced stages of the disease.

The absence of substitution on the aromatic scaffold, monosubstitution, or the presence of a polar catechol moiety completely abolished activity.

**Figure 2.**
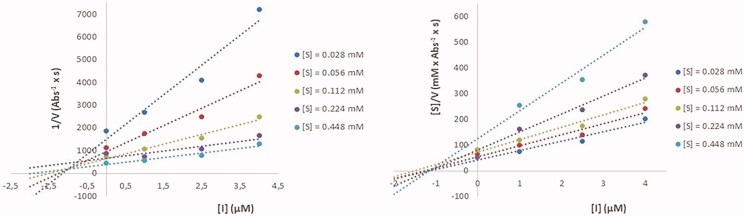
Cornish–Bowden plots (BuChE) for compound **11e**.

Compounds **11e–g** turned out to be either mixed or non-competitive inhibitors, as evidenced by the Cornish–Bowden plots. This means that the title compound can bind either the free enzyme (*K*_ia_) or the complex enzyme-substrate (*K*_ib_). In order to illustrate this issue, Cornish–Bowden plots for derivative **11e** against BuChE (mixed inhibition) are depicted in Figure 2.

#### Docking studies

**Figure 3.**
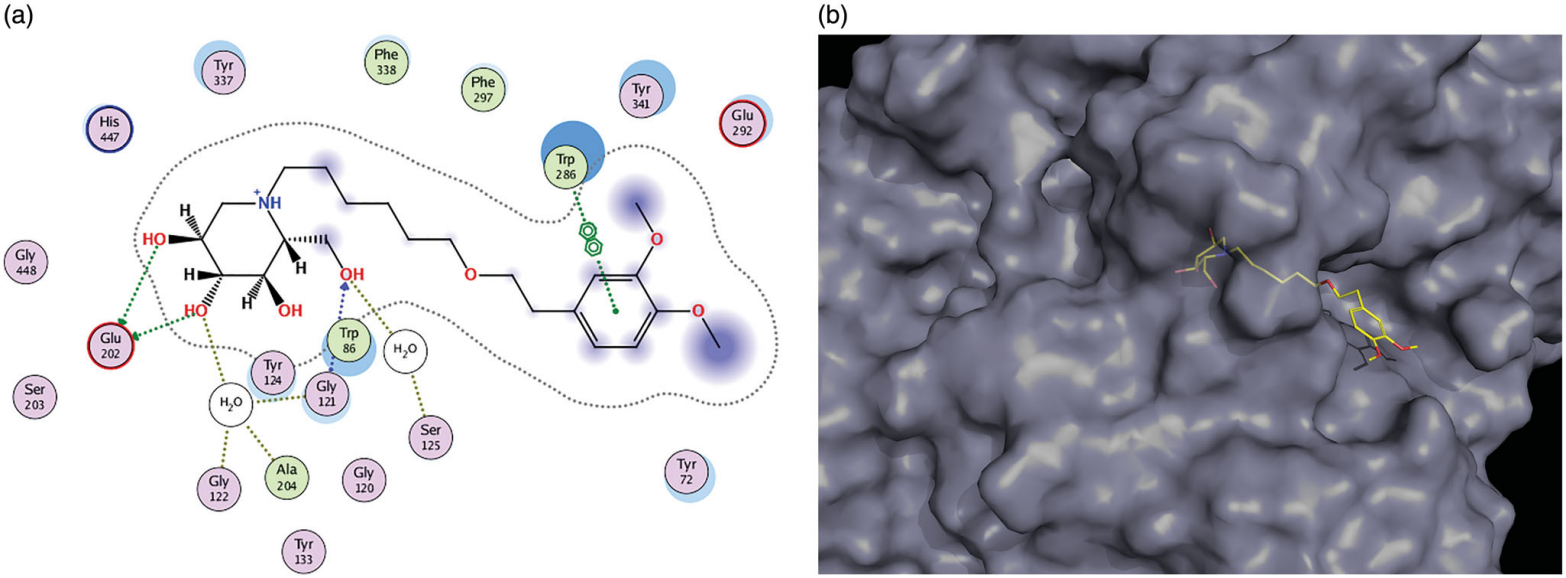
(a, b) Docking simulations for the interactions **11e**-AChE.

Docking calculations predict that compound **11e** forms a complex with AChE (Figure 3). The iminosugar moiety establishes H-bond interactions with Trp86 and Glu202 at the cationic anionic subsite (CAS). Additionally, the aromatic ring moiety is involved in π stacking interactions with Trp286 at the peripheral anionic subsite (PAS). The aromatic ring also participates in van der Waals interactions with several residues at the peripheral anionic subsite.

**Figure 4.**
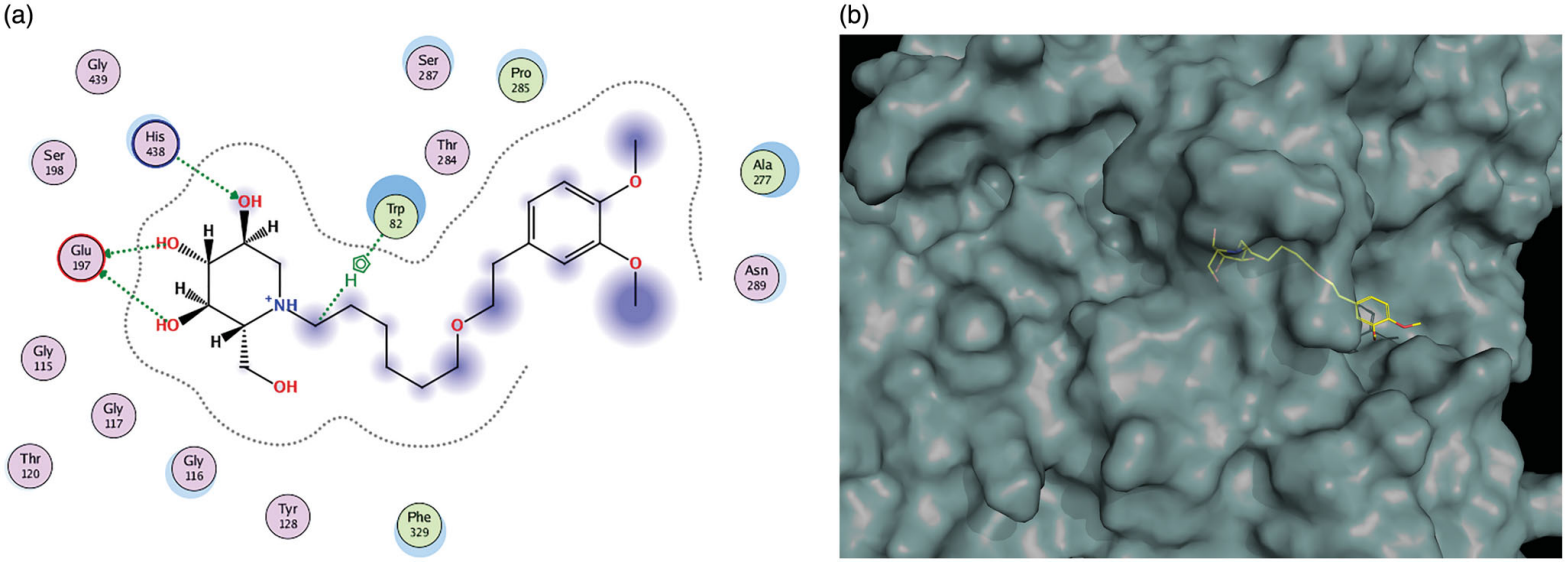
(a, b) Docking simulations for the interactions **11e**-BuChE.

Docking calculations also predict that compound **11e** forms a complex with BuChE (Figure 4). The iminosugar moiety establishes H-bond interactions with Trp82 and Glu197 at the cationic active subsite (CAS) and with His438 at the catalytic subsite. Additionally, the aromatic ring moiety is involved in van der Waals interactions with residues Thr284, Pro285, Asn289 at the peripheral anionic subsite (PAS). Taken together, these calculations predict that **11e** interacts in similar modes with both enzymes (as seen in the 3D figures) except for the interaction with His438 in BuChE which is missing in AChE. The π stacking interactions with Trp286 seen in AChE are not present in BuChE because there is no equivalent residue at the BuChE peripheral anionic subsite.

#### Antiproliferative activity

To analyse the potential cytotoxicity of title compounds, their antiproliferative activity was measured. For that purpose, one solid tumour cell line (SH-SY5Y, glioblastoma) as a model of brain cells, and one non-tumour cell line (BJ-hTERT human fibroblasts) were used. Data show no disruption of cell growth for most of the compounds tested. Only catechol-containing **11d**, exhibited a moderate antiproliferative effect against the non-tumour cell line (GI_50_ = 39 ± 5 µM). Interestingly, the rest of the compounds, and particularly the lead compounds (**11e–g**) lacked significant growth inhibition effects when tested as a concentration as high as 100 µM (GI_50_ > 100 µM).

## Conclusions

We have accomplished the straightforward preparation of hitherto unknown cholinesterases inhibitors based on the use of the iminosugar 1-DNJ as the key pharmacophore for struggling with the low levels of the neurotransmitter acetylcholine in Alzheimer’s disease. Appendage of an ether-connected aromatic motif with different substitution patterns allowed the modulation of the activity towards cholinesterases. Compounds bearing relatively lipophilic disubstituted phenyl moieties (3,4-diOMe, 3-OH/4-OMe, 3,4-OCH_2_O) turned out to be strong inhibitors of AChE, and particularly of BuChE, with IC_50_ and *K*_i_ values within the low micromolar range. The presumptive binding mode of the lead compound to cholinesterases was analysed using molecular docking simulations, revealing interactions with two key regions of the enzyme, CAS, and PAS. Moreover, as well as the strong anticholinergic activity, the lack of remarkable growth inhibition effects at a concentration as high as 100 µM suggests a good profile for the development of future anti-Alzheimer’s drugs based on iminosugars.

## Supplementary Material

Supplemental Material
