# Supplementary material for: Tuning the activity of iminosugars: novel N-alkylated deoxynojirimycin derivatives as strong BuChE inhibitors
Source: J Enzyme Inhib Med Chem. 2020 Nov 23;36(1):138–46. doi: 10.1080/14756366.2020.1847101 (PMC7717699; doi:10.1080/14756366.2020.1847101)
Supplement: Supplemental Material [file IENZ_A_1847101_SM7352.pdf]

Tuning the activity of iminosugars: novel *N*-alkylated deoxynojirimycin derivatives as strong BuChE inhibitors

Ana I. Ahuja-Casarín,[a] Penélope Merino-Montiel,*[,a] José Luis Vega-Baez,[a] Sara Montiel-Smith,[a] Miguel X. Fernandes,[b] Irene Lagunes,[b] Inés Maya,[c] José M. Padrón,*[,b] Óscar López,*[,c] and José G. Fernández-Bolaños[c]

[a]*Facultad de Ciencias Químicas, Ciudad Universitaria, Benemérita Universidad Autónoma de Puebla, 72570, Puebla, PUE, México; e-mail: penelope.merino@correo.buap.mx*

[b]*BioLab, Instituto Universitario de Bio-Orgánica "Antonio González" (IUBO-AG), Universidad de La Laguna, c/ Astrofísico Francisco Sánchez 2, E-38206 La Laguna, Spain, e-mail: jmpadron@ull.edu.es*

[c]*Departamento de Química Orgánica, Facultad de Química, Universidad de Sevilla, Apartado 1203, E-41071 Seville, Spain. e-mail: osc-lopez@us.es; Tel: +34 954 559997*

Synthesis……………………………………………………………………………..……..S2

Ω-Bromoalkyl derivatives ***7,8***………………………………..…………..…**S2–S6**

*O-protected-*N*-Alkyl 1-DNJ derivatives* ***9,10***…………………………….…….**S6–S14**

Preparation of O*-unprotected 1-DNJ derivatives* ***11,12***……………..…**S14–S19**

Table S1……………………………………………………………………………....S20

^{1}H- and ^{13}C-NMR spectra……………………..………..……………………S22-S76

Synthesis

2-Arylethanol derivatives 14b,d,f,g

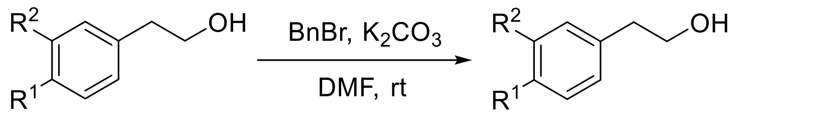

13b R^1=OH, R_2=H
13d R^1=R_2=OH
13f R^1=OH, R_2=OMe

14b R^1=OBn, R^2=H (87%)
14d R^1=R^2=OBn (74%)
14f R^1=OBn, R^2=OMe (95%)

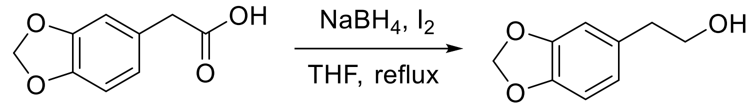

15

14g (quant.)

Alkylation of 14a-g

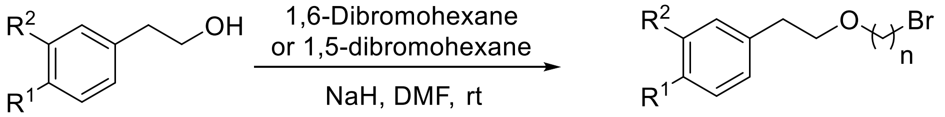

14a-g

n = 6

7a (quant.); **7b** (85%); **7c** (94%)
7d (12%); **7e** (quant.); **7f** (75%)
7g (73%)

n = 5
8a (81%)

{2’-[(6’’-Bromohexyl)oxy]ethyl}benzene (7a). 2-Phenylethanol **14a** (268 mg, 2.19 mmol) was used. Column chromatography (cyclohexane → 49:1 cyclohexane–EtOAc) afforded **7a** (625 mg, quant.). R_f 0.71 (7:3 cyclohexane–EtOAc); ^{1}H-NMR (300 MHz, $CDCl_3$) δ 7.19 (m, 5H, Ar-H), 3.57 (t, 2H, $J_{1',2'}$=7.2 Hz, H-2´), 3.38 (t, 2H, $J_{1'',2''}$=6.5 Hz, H-1´´), 3.32 (t, 2H, $J_{5'',6''}$=6.8Hz, H-6´´), 2.84 (t, 2H, H-1´), 1.79 (m, 2H, H-5´´), 1.53 (m, 2H, H-2´´), 1.34 (m, 4H, H-3´´, H-4´´) ppm; ^{13}C-NMR (75.5 MHz, $CDCl_3$) δ

139.1 (Ar-C*ipso*), 128.9 (Ar-C*m*), 128.3 (Ar-C*o*), 126.1 (Ar-C*p*), 71.8 (C-1´´), 70.7 (C-2´), 36.4 (C-1´), 33.8 (C-6´´), 32.7 (C-5´´), 29.5 (C-4´´), 27.9 (C-2´´), 25.4 (C-3´´) ppm; HRESI–MS calcd. for $C_{14}H_{21}BrNaO$ ([M+Na]$^+$): 307.0668, found: 307.0668.

1-Benzyloxy-4-{2’-[(6’’-bromohexyl)oxy]ethyl}benzene (7b). 2-(4’-Benzyloxyphenyl)ethanol **14b** (500 mg, 2.19 mmol) was used. Column chromatography (cyclohexane → 19:1 cyclohexane–EtOAc) afforded **7b** (728 mg, 85%). R_f 0.69 (7:3 cyclohexane–EtOAc); ^{1}H-NMR (300 MHz, $CDCl_3$) δ 7.39 (m, 5H, Ar-H Bn), 7.15 (m, 2H, Ar-H), 6.94 (m, 2H, Ar-H), 5.06 (s, 2H, C*H*2Ph), 3.61 (t, 2H, $J_{1',2'}$=7.2 Hz, H-2´), 3.45 (t, 2H, $J_{1'',2''}$=6.5 Hz, H-1´´), 3.41 (t, 2H, $J_{6'',5''}$=6.8 Hz, H-6´´), 2.85 (t, 2H, H-1´), 1.87 (m, 2H, H-5´´), 1.60 (m, 2H, H-2´´), 1.42 (m, 4H, H-3´´, H-4´´) ppm; ^{13}C-NMR (75.5 MHz, $CDCl_3$) δ 157.3 (C-1), 137.2 (C-1´´´), 131.4 (C-4), 129.8 (C-3, C-5), 128.5 (Ar-C Bn), 127.8 (Ar-C Bn), 127.4 (Ar-C Bn), 114.7 (C-2, C-6), 72.0 (C-1´´), 70.7 (*C*H_2Ph), 70.0 (C-2´), 35.5 (C-1´), 33.8 (C-6´´), 32.7 (C-5´´), 29.5 (C-4´´), 27.9 (C-2´´), 25.4 (C-3´´) ppm; HRESI–MS calcd. for $C_{21}H_{27}BrNaO_2$ ([M+Na]$^+$): 413.1087, found: 413.1083.

1-{2’-[(6’’-Bromohexyl)oxy]ethyl}-4-methoxybenzene (7c). 2-(4’-Methoxyphenyl)ethanol **14c** (333 mg, 2.19 mmol) was used. Column chromatography (cyclohexane → 46:1 cyclohexane–EtOAc) afforded **7c** (653 mg, 94%). R_f 0.65 (7:3 cyclohexane–EtOAc); ^{1}H-NMR (300 MHz, $CDCl_3$) δ 7.14 (m, 2H, Ar-H*o*), 6.84 (m, 2H, Ar-H*m*), 3.77 (s, 3H, OMe), 3.59 (t, 2H $J_{1',2'}$=7.2 Hz, H-2´), 3.43 (t, 2H, $J_{1'',2''}$=6.5 Hz, H-1´´), 3.40 (t 2H, $J_{5'',6''}$=6.8Hz, H-6´´), 2.83 (t, 2H, H-1´), 1.85 (m, 2H, H-5´´), 1.59 (m, 2H, H-2´´), 1.41 (m, 4H, H-3´´, H-4´´) ppm; ^{13}C-NMR (75.5 MHz, $CDCl_3$) δ 158.1 (C-4), 131.2 (C-1), 129.8 (C-2, C-6), 113.8 (C-3, C-5), 72.1 (C-1´´), 70.8 (C-2´), 55.3 (OMe), 35.5 (C-1´), 33.9 (C-6´´), 32.8 (C-5´´), 29.6 (C-4´´), 28.0 (C-2´´), 25.4 (C-3´´) ppm; HRESI–MS calcd. for $C_{15}H_{23}BrNaO_2$ ([M+Na]$^+$): 337.0774, found: 337.0773.

1,2-Dibenzyloxy-4-{2’-[(6’’-bromohexyl)oxy]ethyl}benzene (7d). 2-(3’,4’-Dibenzyloxy)ethanol **14d** (732 mg, 2.19 mmol) was used. Column chromatography (cyclohexane → 49:1 cyclohexane–EtOAc) afforded **7d** (135 mg, 12%). R_f 0.68 (7:3 cyclohexane–EtOAc); ^{1}H-NMR (300 MHz, $CDCl_3$) δ 7.36–7.09 (m, 10H, Ar-H Bn), 6.77-6.74 (m, 2H, H-5, H-6), 6.62 (m, 1H, H-3), 5.03 (s, 2H, CH_2Ph), 5.01 (s, 2H, CH_2Ph), 3.45 (t, 2H, $J_{1',2'}$=7.2 Hz, H-2´), 3.32–3.24 (m, 4H, H-1´´, H-6´´), 2.67 (t, 2H, H-1´), 1.72 (m, 2H, H-5´´), 1.45 (m, 2H, H-2´´), 1.38–1.15 (m, 4H, H-3´, H-4´´) ppm; ^{13}C-NMR (75.5 MHz, $CDCl_3$) δ 148.9 (C-2), 147.5 (C-1), 137.6 (Ar-C_{ipso} Bn), 137.4 (Ar-C_{ipso} Bn), 132.7 (C-4), 128.5 (Ar-C Bn), 127.8 (Ar-C Bn), 127.7 (Ar-C Bn), 127.4 (Ar-C Bn), 127.3 (Ar-C Bn), 121.8 (C-5), 116.2 (C-3), 115.3 (C-6), 71.9 (C-2’), 71.5 (*C*H_2Ph), 71.4 (*C*H_2Ph), 70.8 (C-2´), 35.9 (C-1´), 33.9 (C-6´´), 32.8 (C-5´´), 29.5 (C-4´´), 28.0 (C-2´´), 25.4 (C-3´´) ppm; HRESI–MS calcd. for $C_{28}H_{33}BrNaO_3$ ($[M+Na]^+$): 519.1505, found: 519.1501

4-{2’-[(6’’-Bromohexyl)oxy]ethyl}-1,2-dimethoxybenzene (7e). 2-(3’,4’-Dimethoxyphenyl)ethanol **14e** (400 mg, 2.19 mmol) was used. Column chromatography (cyclohexane → 19:1 cyclohexane–EtOAc) afforded **7e** (552 mg, 73%). R_f 0.59 (7:3 cyclohexane–EtOAc); ^{1}H-NMR (300 MHz, $CDCl_3$) δ 6.75 (m, 3H, H-3, H-5, H-6), 3.86 (s, 3H, OMe), 3.84 (s, 3H, OMe), 3.59 (t, 2H, $J_{1',2'}$=7.2 Hz, H-2´), 3.42 (t, 2H, $J_{1'',2''}$=6.5 Hz, H-1´´), 3.38 (t, 2H, $J_{5'',6''}$=6.8 Hz, H-6´´), 2.81 (t, 2H, $J_{1',2'}$=7.2 Hz, H-1´), 1.84 (m, 2H, H-5´´), 1.57 (m, 2H, H-2´´), 1.39 (m, 4H, H-3´´, H-4´´) ppm; ^{13}C-NMR (75.5 MHz, $CDCl_3$) δ 148.8, 147.5 (C-1, C-2), 131.8 (C-4), 120.8 (C-5), 112.4 (C-3), 111.2 (C-6), 72.0 (C-1´´), 70.8 (C-2´), 56.0 (OMe), 55.9 (OMe), 36.0 (C-1´), 33.9 (C-6´´), 32.8 (C-5´´), 29.6 (C-4´´), 28.0 (C-2´´), 25.4 (C-3´´) ppm; HRESI–MS calcd. for $C_{16}H_{25}BrNaO_3$ ($[M+Na]^+$): 367.0879, found: 367.0876.

1-Benzyloxy-4-{2'-[(6''-bromohexyl)oxy]ethyl}-2-methoxybenzene (7f). 2-(4'-Benzyloxyl-3'-methoxyphenyl)ethanol **14f** (566 mg, 2.19 mmol) was used. Column chromatography (cyclohexane → 19:1 cyclohexane–EtOAc) afforded **7g** (692 mg, 75%). R_f 0.67 (7:3 cyclohexane–EtOAc); ^{1}H-NMR (300 MHz, $CDCl_3$) δ 7.22 (m, 5H, Ar-H Bn), 6.62 (m, 3H, H-3, H-5, H-6), 5.00 (s, 2H, CH_2Ph), 3.76 (s, 3H, OMe), 3.49 (t, 2H, $J_{1',2'}$=7.1 Hz, H-2´), 3.32 (t, 2H, $J_{1'',2''}$=6.5 Hz, H-1´´), 3.27 (t, 2H, $J_{5',-6''}$=6.8 Hz, H-6´´), 2.71 (t, 2H, H-1´), 1.73 (m, 2H, H-5´´), 1.47 (m, 2H, H-2´´), 1.28 (m, 4H, H-3´´, H-4´´) ppm; ^{13}C-NMR (75.5 MHz, $CDCl_3$) δ 149.6 (C-2), 146.7 (C-1), 137.5 (Ar-C_{ipso} Bn), 132.5 (C-4), 128.6 (Ar-C*m* Bn), 127.8 (Ar-C*p* Bn), 127.3 (Ar-C*o* Bn), 120.9 (C-5), 114.3, 113.0 (C-3, C-6), 72.0 (C-2'), 71.2 (C-1''), 70.9 (*C*H_2Ph), 56.1 (OMe), 36.0 (C-1´), 34.0 (C-6´´), 32.8 (C-5´´), 29.6, 28.0, 25.5 (C-2'', C-3'', C-4'') ppm; HRESI–MS calcd. for $C_{22}H_{29}BrNaO_3$ ($[M + Na]^+$): 443.1192, found: 443.1188.

4-{2'-[(6''-Bromohexyl)oxy]ethyl}-1,2-(methylenedioxy)benzene (7g). 2-(3',4'-Methylenedioxyphenyl)ethanol **14g** (264 mg, 2.19 mmol) was used. Column chromatography (cyclohexane → 49:1 cyclohexane–EtOAc) afforded **7g** (721 mg, quantitative). R_f 0.64 (7:3 cyclohexane–EtOAc); ^{1}H-NMR (300 MHz, $CDCl_3$) δ 6.62 (m, 3H, H-3, H-5, H-6), 5.84 (s, 2H, OCH_2O), 3.49 (t, 2H, $J_{1',2'}$=7.1 Hz, H-2´), 3.37–3.29 (m, 4H, H-1´´, H-6´´), 2.71 (t, 2H, H-1´), 1.77 (m, 2H, H-5´´), 1.50 (m, 2H, H-2´´), 1.30 (m, 4H, H-3´´, H-4´´) ppm; ^{13}C-NMR (75.5 MHz, $CDCl_3$) δ 147.6 (C-2), 146.0 (C-1), 133.0 (C-4), 121.8 (C-5), 109.5 (C-3), 108.2 (C-6), 100.9 (OCH_2O), 72.0 (C-1´´), 70.8 (C-2´), 36.1 (C-1´), 33.9 (C-6´´), 32.8 (C-5´´), 29.6 (C-4´´), 28.1 (C-2´´), 25.5 (C-3´´) ppm; HRESI–MS calcd. for $C_{15}H_{21}BrNaO_3$ ($[M+Na]^+$): 351.0566, found: 351.0563.

{2'-[(6''-Bromopentyl)oxy]ethyl}benzene (8a). 2-Phenylethanol **14a** (268 mg, 2.19 mmol) was used. Column chromatography (cyclohexane → 49:1 cyclohexane–EtOAc) afforded **8a** (482 mg, 81%). R_f 0.78 (7:3 cyclohexane–EtOAc); ^{1}H-NMR (300 MHz,

$CDCl_3$) δ 7.28–7.01 (m, 5H, Ar-H), 3.55 (t, 2H, $J_{1´,2´}$=7.2 Hz, H-2´), 3.36 (t, 2H, $J_{1´´,2´´}$=6.3 Hz, H-1´´), 3.31 (t, 2H, $J_{5´´,6´´}$=6.8 Hz, H-6´´), 2.80 (t, 2H, H-1´), 1.78 (m, 2H, H-5´´), 1.52 (m, 2H, H-2´´), 1.45-1.33 (m, 4H, H-3´´, H-4´´) ppm; ^{13}C-NMR (75.5 MHz, $CDCl_3$) δ 139.2 (C-1), 129.0 (C-2, C-6), 128.4 (C-3, C-5), 126.3 (C-4), 71.9 (C-1´´), 70.7 (C-2´), 36.5 (C-1´), 33.9 (C-5´´), 32.7 (C-4´´), 28.9 (C-2´´), 25.0 (C-3´´) ppm; HRESI–MS calcd. for $C_{13}H_{19}BrNaO$ ($[M+Na]^+$): 293.0511, found: 293.0514.

_N_-Alkylation of _O_-protected 1-DNJ 6

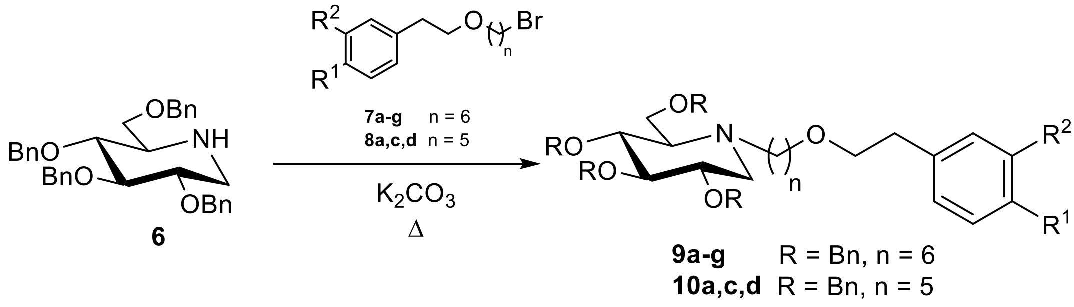

2,3,4,6-Tetra-_O_-benzyl-_N_-[6’-(2’’-phenylethoxy)hexyl]-1-deoxynojirimycin (9a). Compound **7a** (339 mg, 1.19 mmol) was used. Column chromatography (9:1→17:3 cyclohexane–EtOAc) gave **9a** (195 mg, 56%). R_f 0.60 (7:3 cyclohexane–EtOAc); $[\alpha]_D^{25}$ +5 (*c* 1.16, CH_2Cl_2); ^{1}H-NMR (300 MHz, $CDCl_3$) δ 7.33–7.09 (m, 25H, Ar-H), 4.86 (m, 3H, C*H*$_2$Ph), 4.65 (m, 2H, C*H*$_2$Ph), 4.43 (m, 3H, C*H*$_2$Ph), 3.69–3.61 (m, 2H, H-2, H-4), 3.61–3.47 (m, 4H, H-6´, H-1´´), 3.44 (m, 1H, H-6a), 3.41–3.35 (m, 2H, H-3, H-6b), 3.06 (dd, 1H, $J_{1a,1b}$=11.1 Hz, $J_{1a,2}$=4.9 Hz, H-1a), 2.87 (t, 2H, $J_{1´´,2´´}$=7.3 Hz, H-2´´), 2.59 (m, 2H, H-1´), 2.26 (brd, 1H, $J_{1a,1b}$ = 9.5 Hz, H-1b), 2.20 (t, 1H, $J_{4,5}$= $J_{5,6}$= 10.8 Hz, H-5), 1.52 (m, 2H, H-5´), 1.36–1.05 (m, 6H, H-2´, H-3´, H-4´) ppm; ^{13}C-NMR (75.5 MHz, $CDCl_3$) δ 139.2 (C-1´´´), 138.7 (Ar-C$_{ipso}$ Bn), 138.7 (Ar-C$_{ipso}$ Bn), 137.9 (Ar-C$_{ipso}$ Bn), 129.0 (x5) (Ar-C), 128.5 (Ar-C Bn), 128.5 (Ar-C Bn), 128.4 (Ar-C Bn), 128.4 (Ar-C

Bn), 128.0 (Ar-C Bn), 128.0 (Ar-C Bn), 127.7 (Ar-C Bn), 127.6 (Ar-C Bn), 127.5 (Ar-C Bn), 126.2 (Ar-C Bn), 87.5 (C-3), 78.7 (C-4), 78.7 (C-2), 75.4 (CH$_2$Ph), 75.3 (CH$_2$Ph), 73.5 (CH$_2$Ph), 72.8 (CH$_2$Ph), 71.9 (C-6´), 71.0 (C-1´´), 65.4 (C-6), 63.8 (C-5), 54.6 (C-1), 52.4 (C-1´), 36.5 (C-2´´), 29.8 (C-5´), 27.5 (C-2´), 26.2 (C-3´), 23.7 (C-4´) ppm; HRESI–MS calcd. for $C_{48}H_{58}NO_5$ ($[M+H]^+$): 728.4310, found: 728.4299.

2,3,4,6-Tetra-*O*-benzyl-*N*-{6'-[2''-(p-benzyloxyphenyl)ethoxy]hexyl}-1-deoxynojirimycin (9b). Compound **7b** (466 mg, 1.19 mmol) was used. Column chromatography (17:3 cyclohexane–EtOAc) gave **9b** (180 mg, 45%). R_f 0.47 (7:3 cyclohexane–EtOAc); $[\alpha]_D^{25}$ +5 (c 1.0, CH_2Cl_2); ^{1}H-NMR (300 MHz, $CDCl_3$) δ 7.27 (m, 23H, Ar-H Bn), 7.08 (m, 4H, Ar-H), 6.84 (m, 2H, Ar-H), 4.97 (s, 2H, CH_2Ph), 4.82 (m, 3H, CH_2Ph), 4.61 (m, 2H, CH_2Ph), 4.39 (m, 3H, CH_2Ph), 3.65–3.56 (m, 2H, H-2, H-4), 3.56 (m, 1H, H-6b), 3.55–3.48 (m, 4H, H-6´, H-1´´), 3.45 (m, 1H, H-6a), 3.38-3.31 (m, 2H, H-3, H-6b), 3.03 (dd, 1H, $J_{1a,1b}$=11.1 Hz, $J_{1a,2}$ =4.8 Hz, H-1a), 2.77 (t, 2H, $J_{1'',2''}$=7.3 Hz, H-2´´), 2.56 (m, 2H, H-1´), 2.26 (t, 1H, $J_{4,5}$= $J_{5,6}$= 10.8 Hz, H-5), 2.16 (brd, 1H, $J_{1a,1b}$ = 9.4 Hz, H-1b), 1.47 (m, 2H, H-5´), 1.20 (m, 6H, H-2´, H-3´, H-4´) ppm; ^{13}C-NMR (75.5 MHz, $CDCl_3$) δ 157.4 (C-4´´´), 139.2 (Ar-C_{ipso} Bn), 138.7 (Ar-C_{ipso} Bn), 138.7 (Ar-C_{ipso} Bn), 137.9 (Ar-C_{ipso} Bn), 137.3 (Ar-C_{ipso} Bn), 131.5 (C-1´´´), 129.9 (C-2´´´, C-6´´´), 128.7 (Ar-C Bn), 128.5 (Ar-C Bn), 128.5 (Ar-C Bn), 128.4 (Ar-C Bn), 128.4 (Ar-C Bn), 128.0 (Ar-C Bn), 128.0 (Ar-C Bn), 128.0 (Ar-C Bn), 127.7 (Ar-C Bn), 127.6 (Ar-C Bn), 127.5 (Ar-C Bn), 127.5 (Ar-C Bn), 114.9 (C-3´´´, C-5´´´), 87.5 (C-3), 78.7 (C-4), 78.7 (C-2), 75.4 (CH$_2$Ph), 75.3 (CH$_2$Ph), 73.5 (CH$_2$Ph), 72.8 (CH$_2$Ph), 72.2 (C-6´), 71.0 (C-1´´), 70.1 (CH$_2$Ph), 65.4 (C-6), 63.8 (C-5), 54.6 (C-1), 52.5 (C-1´), 35.6 (C-2´´), 29.8 (C-5´), 27.5 (C-2´), 26.2 (C-3´), 23.7 (C-4´); HRESI–MS calcd. for $C_{55}H_{64}NO_6$ ($[M+H]^+$): 834.4728, found: 834.4720.

2,3,4,6-Tetra-*O*-benzyl-*N*-{6'-[(2''-p-methoxyphenyl)ethoxy]hexyl}-1-deoxynojirimycin (9c). Compound **7c** (394 mg, 1.19 mmol) was used. Column chromatography (9:1→19:3 cyclohexane–EtOAc) gave **9c** (167 mg, 46%). R_f 0.53 (7:3 cyclohexane–EtOAc); $[\alpha]_D^{25}$ +8 (*c* 1.07, CH_2Cl_2); ^{1}H-NMR (300 MHz, $CDCl_3$) δ 7.22 (m, 18H, Ar-H Bn), 7.08 (m, 4H, Ar-H), 6.77 (m, 2H, Ar-H), 4.85 (m, 3H, CH_2Ph), 4.61 (m, 2H, CH_2Ph), 4.39 (m, 3H, CH_2Ph), 3.70 (s, 3H, OMe), 3.65–3.57 (m, 2H, H-2, H-3), 3.56–3.48 (m, 4H, H-6´, H-1´´), 3.43 (m, 1H, H-6a), 3.38–3.30 (m, 2H, H-4, H-6b), 3.03 (dd, 1H, $J_{1a,1b}$=11.1 Hz, $J_{1a,2}$=4.8 Hz, H-1a), 2.70 (t, 2H, $J_{1'',2''}$=7.3 Hz, H-2´´), 2.56 (m, 2H, H-1´), 2.24 (brd, 1H, $J_{1a,1b}$= 9.5 Hz, H-1b), 2.17 (t, 1H, $J_{4,5}$= $J_{5,6}$= 10.8 Hz, H-5), 1.48 (m, 2H, H-5´), 1.41–1.03 (m, 6H, H-2´, H-3´, H-4´) ppm; ^{13}C-NMR (75.5 MHz, $CDCl_3$) δ 158.1 (C-4´´´), 139.1 (Ar-C$_{ipso}$ Bn), 138.7 (Ar-C$_{ipso}$ Bn), 138.7 (Ar-C$_{ipso}$ Bn), 137.9 (Ar-C$_{ipso}$ Bn), 131.2 (C-1´´´), 129.9 (C-2´´´, C-6´´´), 128.5 (Ar-C Bn), 128.4 (Ar-C Bn), 128.4 (Ar-C Bn), 128.4 (Ar-C Bn), 127.9 (Ar-C Bn), 127.9 (Ar-C Bn), 127.7 (Ar-C Bn), 127.6 (Ar-C Bn), 127.5 (Ar-C Bn), 113.8 (C-3´´´, C-5´´´), 87.5 (C-3), 78.7 (C-4), 78.6 (C-2), 75.4 (*C*H$_2$Ph), 75.2 (*C*H$_2$Ph), 73.5 (*C*H$_2$Ph), 72.8 (*C*H$_2$Ph), 72.2 (C-6´), 71.0 (C-1´´), 65.4 (C-6), 63.8 (C-5), 55.3 (OMe), 54.5 (C-1), 52.4 (C-1´), 35.5 (C-2´´), 29.8 (C-5´), 27.4 (C-2´), 26.2 (C-3´), 23.7 (C-4´) ppm; HRESI–MS calcd. for $C_{49}H_{60}NO_6$ ($[M+H]^+$): 758.4415, found: 758.4406.

2,3,4,6-Tetra-*O*-benzyl-*N*-{6'-[2''-(3''',4'''-dibenzyloxyphenyl)ethoxy]hexyl}-1-deoxynojirimycin (9d). Compound **7d** (592 mg, 1.19 mmol) was used. Column chromatography (9:1→17:3 cyclohexane–EtOAc) gave **9d** (226 mg, 50%). R_f 0.50 (7:3 cyclohexane–EtOAc); $[\alpha]_D^{25}$ +4 (*c* 0.96, CH_2Cl_2); ^{1}H-NMR (300 MHz, $CDCl_3$) δ 7.23 (m, 30H, Ar-H Bn), 6.76 (m, 2H, H-5''', H-6'''), 6.66 (m, 1H, H-2'''), 5.05 (s, 2H, CH_2Ph), 5.03 (s, 2H, CH_2Ph) 4.81 (m, 3H, CH_2Ph), 4.57 (m, 2H, CH_2Ph), 4.35 (m, 3H, CH_2Ph), 3.61–3.53 (m, 2H, H-2, H-4), 3.51–3.42 (m, 4H, H-6´, H-1´´), 3.37 (m, 1H, H-

6a), 3.34–3.28 (m, 2H, H-3, H-6b), 3.00 (dd, 1H, $J_{1a,1b}$=11.1 Hz, $J_{1a,2}$=4.8 Hz, H-1a), 2.70 (t, 2H, $J_{1'',2''}$=7.3 Hz, H-2´´), 2.53 (m, 2H, H-1´), 2.20 (brd, 1H, $J_{1a,1b}$= 9.5 Hz, H-1b), 2.12 (t, 1H, $J_{4,5}$=10.8 Hz, H-5), 1.45 (m, 2H, H-5´), 1.17 (m, 6H, H-2´, H-3´, H-4') ppm; ^{13}C-NMR (75.5 MHz, $CDCl_3$) δ 149.0 (C-4´´´), 147.6 (C-3´´´), 139.1 (Ar-C*ipso* Bn), 138.7 (Ar-C*ipso* Bn), 138.7 (Ar-C*ipso* Bn), 137.9 (Ar-C*ipso* Bn), 137.6 (Ar-C*ipso* Bn), 137.5 (Ar-C*ipso* Bn), 132.7 (C-1´´´), 128.5 (Ar-C Bn), 128.5 (Ar-C Bn), 128.4 (Ar-C Bn), 128.4 (Ar-C Bn), 128.0 (Ar-C Bn), 127.9 (Ar-C Bn), 127.9 (Ar-C Bn), 127.8 (Ar-C Bn), 127.7 (Ar-C Bn), 127.6 (Ar-C Bn), 127.5 (Ar-C Bn), 127.5 (Ar-C Bn), 127.4 (Ar-C Bn), 121.8 (C-6´´´), 116.2 (C-2´´´), 115.4 (C-5´´´), 87.5 (C-3), 78.7 (C-4), 78.7 (C-2), 75.4 (CH_2Ph), 75.3 (CH_2Ph), 73.5 (CH_2Ph), 72.8 (CH_2Ph), 72.0 (C-6´), 71.6 (CH_2Ph), 71.5 (CH_2Ph), 71.1 (C-1´´), 65.4 (C-6), 63.8 (C-5), 54.6 (C-1), 52.4 (C-1´), 36.0 (C-2´´), 29.8 (C-5´), 27.5 (C-2´), 26.2 (C-3´), 23.7 (C-4´) ppm; HRSESI–MS calcd. for $C_{62}H_{70}NO_7$ ([M+H]$^+$): 940.5147, found: 940.5138.

2,3,4,6-Tetra-*O*-benzyl-*N*-{6'-[2''-(3''',4'''-dimethoxyphenyl)ethoxy]hexyl}-1-deoxynojirimycin (9e). Compound **7e** (411 mg, 1.19 mmol) was used. Column chromatography (9:1→19:3 cyclohexane–EtOAc) gave **9e** (257 mg, 68%). R_f 0.31 (7:3 cyclohexane–EtOAc); $[\alpha]_D^{25}$ +5 (*c* 1.04, CH_2Cl_2); ^{1}H-NMR (300 MHz, $CDCl_3$) δ 7.18 (m, 21H, Ar-H), 6.71 (m, 2H, Ar-H), 4.83 (m, 3H, CH_2Ph), 4.61 (m, 2H, CH_2Ph), 4.39 (m, 3H, CH_2Ph), 3.80 (s, 3H, OMe), 3.78 (s, 3H, OMe), 3.66–3.58 (m, 2H, H-2, H-4), 3.58–3.45 (m, 4H, H-6´, H-1´´), 3.40 (m, 1H, H-6a), 3.38–3.32 (m, 2H, H-3, H-6b), 3.03 (dd, 1H, $J_{1a,1b}$=11.1 Hz, $J_{1a,2}$=4.8 Hz, H-1a), 2.78 (t 2H, $J_{1'',2''}$=7.3 Hz, H-2´´), 2.55 (m, 2H, H-1´), 2.23 (brd, 1H, $J_{1a,1b}$= 9.3 Hz, H-1b), 2.17 (t, 1H, $J_{4,5}$=10.8 Hz, H-5), 1.50 (m, 2H, H-5´), 1.22 (m, 6H, H-2´, H-3´, H-4´) ppm; ^{13}C-NMR (75.5 MHz, $CDCl_3$) δ 148.8 (C-4´´´), 147.5 (C-3´´´), 139.1 (Ar-C*ipso* Bn), 138.6 (Ar-C*ipso* Bn), 138.6 (Ar-C*ipso* Bn), 137.9 (Ar-C*ipso* Bn), 131.8 (C-1´´´), 128.5 (Ar-C), 128.4 (Ar-C), 128.4 (Ar-C),

128.3 (Ar-C), 127.9 (Ar-C), 127.9 (Ar-C), 127.7 (Ar-C), 127.6 (Ar-C), 127.5 (Ar-C), 120.8 (C-6´´´), 112.3 (C-2´´´), 111.3 (C-5´´´), 87.4 (C-3), 78.7 (C-4), 78.6 (C-2), 75.3 (*C*H$_2$Ph), 75.2 (*C*H$_2$Ph), 73.5 (*C*H$_2$Ph), 72.8 (C-6´), 72.1 (C-1´´), 71.0 (*C*H$_2$Ph), 65.4 (C-6), 63.8 (C-5), 56.0 (OMe), 55.9 (OMe), 54.5 (C-1), 52.4 (C-1´), 36.0 (C-2´´), 29.8 (C-5´), 27.4 (C-2´), 26.2 (C-3´), 23.7 (C-4´) ppm; HRESI–MS calcd. for $C_{50}H_{62}NO_7$ ($[M+H]^+$): 788.4521, found: 788.4514.

2,3,4,6-Tetra-*O*-benzyl-*N*-{6'-[2''-(4'''-benzyloxy-3'''-methoxyphenyl)ethoxy]hexyl}-1-deoxynojirimycin (9f). Compound **7f** (503 mg, 1.19 mmol) was used. Column chromatography (9:1 cyclohexane–EtOAc) gave **9f** (278 mg, 67%). $[\alpha]_D^{25}$–2 (*c* 0.77, CH_2Cl_2); R_f 0.51 (7:3 cyclohexane–EtOAc); ^{1}H-NMR (300 MHz, $CDCl_3$) δ 7.23 (m, 25H, Ar-H Bn), 6.69 (m, 3H, H-2''', H-5''', H-6'''), 5.05 (s, 2H, C*H*$_2$Ph), 4.83 (m, 3H, C*H*$_2$Ph), 4.61 (m, 2H, C*H*$_2$Ph), 4.40 (m, 3H, C*H*$_2$Ph), 3.81 (s, 3H, OMe), 3.65–3.57 (m, 2H, H-2, H-4), 3.56–3.46 (m, 4H, H-6´, H-1´´), 3.42 (m, 1H, H-6a), 3.39–3.31 (m, 2H, H-3, H-6b), 3.04 (dd, 1H, $J_{1a,1b}$=11.1 Hz, $J_{1a,2}$=4.8 Hz, H-1a), 2.77 (t, 2H, $J_{1'',2''}$=7.2 Hz, H-2´´), 2.55 (m, 2H, H-1´), 2.23 (brd, 1H, $J_{1a,1b}$= 9.6 Hz, H-1b), 2.17 (t, 1H, $J_{4,5}$=10.7 Hz, H-5), 1.48 (m, 2H, H-5´), 1.33–1.02 (m, 6H, H-2´, H-3´, H-4´) ppm; ^{13}C-NMR (75.5 MHz, $CDCl_3$) δ 149.6 (C-3´´´), 146.7 (C-4´´´), 139.1 (Ar-C$_{ipso}$ Bn), 138.7 (Ar-C$_{ipso}$ Bn), 138.7 (Ar-C$_{ipso}$ Bn), 137.9 (Ar-C$_{ipso}$ Bn), 137.5 (Ar-C$_{ipso}$ Bn), 132.4 (C-1´´´), 128.6 (Ar-C), 128.5 (Ar-C), 128.4 (Ar-C), 128.4 (Ar-C), 128.4 (Ar-C), 127.9 (Ar-C), 127.9 (Ar-C), 127.8 (Ar-C), 127.7 (Ar-C), 127.6 (Ar-C), 127.5 (Ar-C), 127.3 (Ar-C), 120.8 (C-6´´´), 114.3 (C-5´´´´), 113.0 (C-2´´´), 87.5 (C-3), 78.7 (C-4), 78.6 (C-2), 75.4 (*C*H$_2$Ph), 75.2 (*C*H$_2$Ph), 73.5 (*C*H$_2$Ph), 72.8 (*C*H$_2$Ph), 72.1 (C-6´), 71.3 (C-1´´), 71.1 (*C*H$_2$Ph), 65.4 (C-6), 63.8 (C-5), 56.1 (OMe), 54.6 (C-1), 52.4 (C-1´), 36.1 (C-2´´), 29.8 (C-5´), 27.5 (C-2´), 26.2 (C-3´), 23.7 (C-4´) ppm; HRESI–MS calcd. for $C_{56}H_{66}NO_7$ ($[M+H]^+$): 864.4834, found: 864.4826.

2,3,4,6-Tetra-*O*-benzyl-*N*-{6'-[2''-(3''',4'''-methylenedioxyphenyl)ethoxy]hexyl}-1-deoxynojirimycin (9g). Compound **7f** (390 mg, 1.19 mmol) was used. Column chromatography (9:1→17:3 cyclohexane–EtOAc) gave **9g** (184 mg, 50%). R_f 0.54 (7:3 cyclohexane–EtOAc); $[\alpha]_D^{25}$ +3 (*c* 1.36, CH_2Cl_2); ^{1}H-NMR (300 MHz, $CDCl_3$) δ 7.16 (m, 18H, Ar-H Bn), 6.61 (m, 5H, Ar-H Bn, H-2''', H-5''', H-6'''), 5.80 (s, 2H, OCH_2O), 4.82 (m, 3H, CH_2Ph), 4.59 (m, 2H, CH_2Ph), 4.37 (m, 3H, CH_2Ph), 3.63–3.53 (m, 2H, H-2, H-4), 3.53–3.41 (m, 4H, H-6´, H-1´´), 3.38 (m, 1H, H-6a), 3.35–3.28 (m, 2H, H-3, H-6b), 3.03 (dd, 1H, $J_{1a,1b}$=11.1 Hz, $J_{1a\text{-}2}$ =4.8 Hz, H-1a), 2.71 (t, 2H, $J_{1'',2''}$ =7.1 Hz, H-2´´), 2.52 (m, 2H, H-1´), 2.22 (brd, 1H, $J_{1a,1b}$= 9.3 Hz, H-1b), 2.14 (t, 1H, $J_{4,5}$=10.8 Hz, H-5), 1.44 (m, 2H, H-5´), 1.34–1.00 (m, 6H, H-2´, H-3´, H-4´) ppm; ^{13}C-NMR (75.5 MHz, $CDCl_3$) δ 147.6 (C-4´´´), 146.0 (C-3´´´), 139.1 (Ar-C*ipso* Bn), 138.7 (Ar-C*ipso* Bn), 138.7 (Ar-C*ipso* Bn), 137.9 (Ar-C*ipso* Bn), 133.0 (C-1´´´), 128.5 (Ar-C), 128.4 (Ar-C), 128.4 (Ar-C), 128.4 (Ar-C), 127.9 (Ar-C), 127.9 (Ar-C), 127.7 (Ar-C), 127.6 (Ar-C), 127.5 (Ar-C), 121.7 (C-6´´´), 109.4 (C-5´´´), 108.2 (C-2´´´), 100.8 (OCH_2O), 87.5 (C-3), 78.7 (C-4), 78.6 (C-2), 75.4 (*C*H_2Ph), 75.2 (*C*H_2Ph), 73.5 (*C*H_2Ph), 72.8 (*C*H_2Ph), 72.0 (C-6´), 71.0 (C-1´´), 65.4 (C-6), 63.8 (C-5), 54.5 (C-1), 52.4 (C-1´), 36.1 (C-2´´), 29.8 (C-5´), 27.4 (C-2´), 26.2 (C-3´), 23.7 (C-4´) ppm; HRESI–MS calcd. for $C_{49}H_{58}NO_7$ ($[M+H]^+$): 772.4208, found: 772.4199.

2,3,4,6-Tetra-*O*-benzyl-*N*-[5'-(2''-phenylethoxy)pentyl]-1-deoxynojirimycin (10a). Compound **8a** (324 mg, 1.19 mmol) was used. Column chromatography (19:3→17:3 cyclohexane–EtOAc) gave **10a** (133 mg, 39%). R_f 0.54 (7:3 cyclohexane–EtOAc); $[\alpha]_D^{25}$ +7 (*c* 0.61, CH_2Cl_2); ^{1}H-NMR (300 MHz, $CDCl_3$) δ 7.34–6.97 (m, 25H, Ar-H), 4.80 (m, 3H, CH_2Ph), 4.59 (m, 2H, CH_2Ph), 4.35 (m, 3H, CH_2Ph), 3.63–3.55 (m, 2H, H-2, H-4), 3.54–3.42 (m, 4H, H-5´, H-1´´), 3.37 (m, 1H, H-6a), 3.33–3.29 (m, 2H, H-3, H-6b), 3.00 (dd, 1H, $J_{1a,1b}$=11.1 Hz, $J_{1a\text{-}2}$=4.8 Hz, H-1a), 2.81 (t, 2H, $J_{1'',2''}$=7.3 Hz, H-

2´´), 2.54 (m, 2H, H-1´), 2.22 (brd, 1H, $J_{1a,1b}$= 9.5 Hz, H-1b), 2.15 (t, 1H, $J_{4,5}$=10.8 Hz, H-5), 1.46 (m, 2H, H-2´), 1.32–1.05 (m, 4H, H-3´, H-4´) ppm; ^{13}C-NMR (75.5 MHz, $CDCl_3$) δ 139.1 (C-1´´´), 138.7 (Ar-C*ipso* Bn), 137.9 (Ar-C*ipso* Bn), 129.0 (x5) (Ar-C), 128.5 (Ar-C), 128.5 (Ar-C), 128.4 (Ar-C), 128.4 (Ar-C), 128.0 (Ar-C), 128.0 (Ar-C), 127.7 (Ar-C), 127.6 (Ar-C), 127.5 (Ar-C), 126.3 (Ar-C), 87.5 (C-3), 78.7 (C-4), 78.7 (C-2), 75.4 (CH_2Ph), 75.3 (CH_2Ph), 73.6 (CH_2Ph), 72.9 (CH_2Ph), 72.0 (C-5´), 71.0 (C-1´´), 65.4 (C-6), 63.8 (C-5), 54.6 (C-1), 52.4 (C-1´), 36.5 (C-2´´), 29.7 (C-4´), 24.2 (C-2´), 23.5 (C-3´) ppm; HRESI–MS calcd. for $C_{47}H_{56}NO_5$ ($[M+H]^+$): 714.4153, found: 714.4147.

2,3,4,6-Tetra-*O*-benzyl-*N*-{5’-[2’’-p-methoxyphenyl)ethoxy]pentyl}-1-deoxynojirimycin (10c). Compound **8c** was used (359 mg, 1.19 mmol). Column chromatography (19:3→17:3 cyclohexane–EtOAc) gave **10c** (137 mg, 38%). R_f 0.57 (7:3 cyclohexane–EtOAc); $[\alpha]_D^{25}$ +3 (*c* 0.75, CH_2Cl_2); ^{1}H-NMR (300 MHz, $CDCl_3$) δ 7.25–7.14 (m, 18H, Ar-H Bn), 7.09–7.02 (m, 4H, Ar-H), 6.78–6.71 (m, 2H, Ar-H), 4.85 (m, 3H, CH_2Ph), 4.59 (m, 2H, CH_2Ph), 4.35 (m, 3H, CH_2Ph), 3.69 (s, 3H, OMe), 3.61–3.54 (m, 2H, H-2, H-4), 3.53–3.43 (m, 4H, H-5´, H-1´´), 3.40–3.36 (m, 1H, H-6a), 3.34–3.29 (m, 2H, H-3, H-6b), 3.01 (dd, 1H, $J_{1a,1b}$=11.1 Hz, $J_{1a,2}$=4.8 Hz, H-1a), 2.75 (t, 2H, $J_{1'',2''}$=7.3 Hz, H-2´´), 2.54 (m, 2H, H-1´), 2.22 (brd, 1H, $J_{1a,1b}$= 9.5 Hz, H-1b), 2.14 (t, 1H, $J_{4,5}$=10.8 Hz, H-5), 1.45 (m, 2H, H-2´), 1.33–1.02 (m, 4H, H-3´, H-4´) ppm; ^{13}C-NMR (75.5 MHz, $CDCl_3$) δ 158.2 (C-4´´´), 139.2 (Ar-C*ipso* Bn), 138.7 (Ar-C*ipso* Bn), 138.7 (Ar-C*ipso* Bn), 137.9 (Ar-C*ipso* Bn), 131.2 (C-1´´´), 129.9 (C-2´´´, C-6´´´), 128.6 (Ar-C), 128.5 (Ar-C), 128.4 (Ar-C), 128.2 (Ar-C), 128.0 (Ar-C), 127.9 (Ar-C), 127.7 (Ar-C), 127.6 (Ar-C), 127.5 (Ar-C), 113.9 (C-3´´´, C-5´´´), 87.5 (C-3), 78.7 (C-4), 78.7 (C-2), 75.4 (CH_2Ph), 75.3 (CH_2Ph), 73.6 (CH_2Ph), 72.9 (CH_2Ph), 72.2 (C-5´), 71.0 (C-1´´), 65.4 (C-6), 63.8 (C-5), 55.3 (OMe), 54.6 (C-1), 52.4 (C-1´), 35.6 (C-2´´), 29.7 (C-

4´), 24.2 (C-2´), 23.6 (C-3´) ppm; HRESI–MS calcd. for $C_{48}H_{58}NO_6$ ($[M+H]^+$): 744.4259, found: 744.4250.

2,3,4,6-Tetra-*O*-benzyl-*N*-{5'-[2''-(3''',4'''-dibenzyloxyphenyl)ethoxy]pentyl}-1-deoxynojirimycin (10d). Compound **8d** (577 mg, 1.19 mmol) was used. Column chromatography (19:3→17:3 cyclohexane–EtOAc) gave **10d** (165 mg, 37%). $[\alpha]_D^{25}$ +4 (*c* 0.9, CH_2Cl_2); R_f 0.50 (7:3 cyclohexane–EtOAc); ^{1}H-NMR (300 MHz, $CDCl_3$) δ 7.42–7.00 (m, 30H, Ar-H Bn), 6.84–6.59 (m, 3H, H-2''', H-5''', H-6'''), 5.05 (s, 2H, CH_2Ph), 5.03 (s, 2H, CH_2Ph) 4.81 (m, 3H, CH_2Ph), 4.58 (m, 2H, CH_2Ph), 4.35 (m, 3H, CH_2Ph), 3.63–3.53 (m, 2H, H-2, H-4), 3.52–3.42 (m, 4H, H-5´, H-1´´), 3.37 (m, 1H, H-6a), 3.30–3.26 (m, 2H, H-3, H-6b), 3.00 (dd, $J_{1a\text{-}1b}$=11.1 Hz, $J_{1a\text{-}2}$= 4.8 Hz, 1H, H-1a), 2.70 (t, $J_{2''\text{-}1''}$=7.3 Hz, 2H, H-2´´), 2.51 (m, 2H, H-1´), 2.21 (brd, 1H, $J_{1a,1b}$= 11.0 Hz, H-1b), 2.14 (t, 1H, $J_{4,5}$=10.8 Hz, H-5), 1.43 (m, 2H, H-2´), 1.30–1.03 (m, 4H, H-3´, H-4´) ppm; ^{13}C-NMR (75.5 MHz, $CDCl_3$) δ 149.0 (C-4´´´), 147.6 (C-3´´´), 139.1 (Ar-C$_{ipso}$ Bn), 138.7 (Ar-C$_{ipso}$ Bn), 138.7 (Ar-C$_{ipso}$ Bn), 137.9 (Ar-C$_{ipso}$ Bn), 137.6 (Ar-C$_{ipso}$ Bn), 137.5 (Ar-C$_{ipso}$ Bn), 132.6 (C-1´´´), 128.5 (Ar-C), 128.5 (Ar-C), 128.4 (Ar-C), 128.0 (Ar-C), 128.0 (Ar-C), 127.9 (Ar-C), 127.8 (Ar-C), 127.7 (Ar-C), 127.6 (Ar-C), 127.5 (Ar-C), 127.5 (Ar-C), 127.4 (Ar-C), 121.9 (C-6´´´), 116.3 (C-2´´´), 115.4 (C-5´´´), 87.5 (C-3), 78.7 (C-4), 78.6 (C-2), 75.4 (CH$_2$Ph), 75.3 (CH$_2$Ph), 73.5 (CH$_2$Ph), 72.9 (CH$_2$Ph), 72.0 (C-5´), 71.6 (CH$_2$Ph), 71.5 (CH$_2$Ph), 71.0 (C-1´´), 65.4 (C-6), 63.9 (C-5), 54.6 (C-1), 52.4 (C-1´), 35.9 (C-2´´), 29.7 (C-4´), 24.2 (C-2´), 23.6 (C-3´) ppm; HRESI–MS calcd. for $C_{61}H_{68}NO_7$ ($[M+H]^+$): 926.4990, found: 926.4986.

Deprotection of *O*-protected DNJ derivatives 9. 10

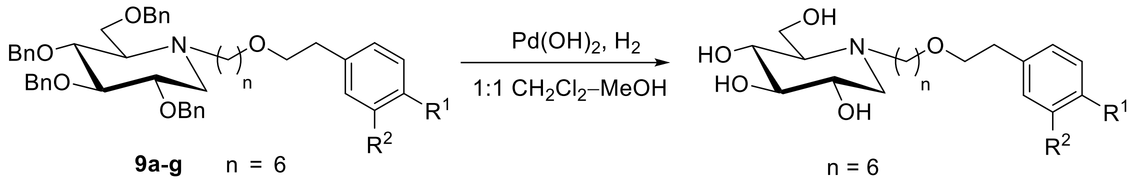

9a-g n = 6
10a,c,d n = 5

n = 6

11a (62%); **11b** (quant.); **11c** (63%)
11d (51%); **11e** (84%); **11f** (91%)
11g (46%)

n = 5

12a (32%), **12c** (32%), **12d** (11%)

***N*-[6’-(2’’-Phenylethoxy)hexyl]-1-deoxynojirimycin (11a).** Compound **9a** (146 mg, 0.20 mmol) was used. Yield: 46 mg, 62%). R_f 0.43 (5:2 CH_2Cl_2–MeOH); $[\alpha]_D^{25}$ –7 (*c* 0.98, MeOH); ^{1}H-NMR (300 MHz, CD_3OD) δ 7.29–7.15 (m, 5H, Ar-H), 3.87 (m, 2H, H-6a, H-6b), 3.63 (t, 2H, $J_{1'',2''}$= 6.9 Hz, H-1’’), 3.50 (m, 1H, H-2), 3.44 (t, 2H, $J_{5'6'}$= 6.4 Hz, H-6´), 3.36 (t, 1H, $J_{3,4}$= $J_{4,5}$= 9.2 Hz, H-4), 3.17 (t, 1H, $J_{2,3}$= 9.0 Hz, H-3), 3.04 (dd, 1H, $J_{1a,2}$= 4.7 Hz, $J_{1a,1b}$= 11.3 Hz, H-1a), 2.82 (t, 1H, H-2’’), 2.89–2.79 (m, 1H, H-1’a), 2.63 (m, 1H, H-1’b), 2.26 (t, $J_{1b,2}$= 10.7 Hz, H-1b), 2.22 (td, 1H, $J_{5,6a}$= $J_{5,6b}$= 2.5 Hz, H-5), 1.60–1.26 (m, 8H, H-2’–H-5’) ppm; ^{13}C-NMR (75.5 MHz, CD_3OD) δ 139.1 (C-1´´´), 128.5 (C-2´´´, C-6´´´), 127.9 (C-3´´´, C-5´´´), 125.8 (C-4´´´), 78.8 (C-3), 71.4 (C-6´), 70.4 (C-1´´), 70.1 (C-4), 68.8 (C-2), 66.0 (C-5), 58.7 (C-6), 57.4 (C-1), 52.4 (C-1´), 35.8 (C-2´´), 29.2 (C-5´), 26.8 (C-2´), 25.7 (C-4´), 23.6 (C-3´) ppm; HRESI-MS calcd. for $C_{20}H_{34}NO_5$ ($[M+H]^+$): 368.2431, found: 368.2436.

***N*-{6’-[2’’-(*p*-Hydroxyphenyl)ethoxy]hexyl}-1-deoxynojirimycin (11b).** Compound **9b** (167 mg, 0.20 mmol) was used. Yield: 77 mg (quant.). R_f 0.42 (5:2 CH_2Cl_2–MeOH); $[\alpha]_D^{25}$–6 (*c* 1.27, MeOH); ^{1}H-NMR (300 MHz, CD_3OD) δ 7.03 (m, 2H, Ar-H*o*), 6.70 (m, 1H, Ar-H*m*), 3.90 (m, 2H, H-6a, H-6b), 3.57 (t, 1H, $J_{1'',2''}$= 7.0 Hz, H-1’’), 3.59–

3.51 (m, 1H, H-2), 3.43 (t 1H, $J_{5',6'}$= 6.3 Hz, H-6'), 3.46–3.41 (m, 1H, H-4), 3.21 (t, 1H, $J_{2,3}$= $J_{3,4}$= 9.1 Hz, H-3), 3.12 (dd, 1H, $J_{1a,2}$= 5.0 Hz, $J_{1a,1b}$= 11.3 Hz, H-1a), 2.99–2.89 (m, 1H, H-1'a), 2.74 (t, 1H, H-2''), 2.82–2.69 (m, 1H, H-1'b), 2.45–2.37 (m, 2H, H-1b, H-5), 1.60–1.43 (m, 4H, H-4', H-5'), 1.41–1.27 (m, 4H, H-2', H-3') ppm; ^{13}C-NMR (75.5 MHz, CD_3OD) δ 156.9 (C-4´´´), 131.3 (C-1´´´), 131.0 (C-2´´´, C-6´´´), 116.2 (C-3´´´, C-5´´´), 80.3 (C-3), 73.4 (C-6´), 71.9 (C-1´´), 71.6 (C-4), 70.4 (C-2), 67.5 (C-5), 58.9 (C-6), 57.3 (C-1), 53.8 (C-1´), 36.5 (C-2´´), 30.7 (C-5´), 28.4 (C-2´), 27.2 (C-4´), 25.2 (C-3´); HRESI–MS calcd. for $C_{20}H_{34}NO_6$ ($[M+H]^+$): 384.2381, found: 384.2386.

***N*-{6'-[2''-(*p*-Methoxyphenyl)ethoxy]hexyl}-1-deoxynojirimycin (11c).** Compound **9c** (152 mg, 0.20 mmol) was used. Yield: 50 mg, 63%. R_f 0.59 (5:2 CH_2Cl_2–MeOH); $[\alpha]_D^{25}$ –8 (*c* 1.07, CH_2Cl_2); ^{1}H-NMR (300 MHz, $(CD_3)_2CO$) δ 7.15 (m, 2H, Ar-H*o*), 6.83 (m, 2H, Ar-H*m*), 4.18 (m, 6H, 5OH, H-6a), 3.87 (m, 1H, H-6b), 3.74 (s, 3H, OMe), 3.67–3.58 (m, 1H, H-2), 3.53 (t, 1H, $J_{1'',2''}$= 7.1 Hz, H-1''), 3.40 (t, 1H, $J_{5',6'}$= 6.4 Hz, H-6'), 3.56-3.52 (m, 1H, H-4), 3.27–3.22 (m, 1H, H-3), 3.06 (m, 1H, H-1a), 2.87–2.83 (m, 1H, H-1'a), 2.76 (t, 2H, H-2''), 2.58 (m, 1H, H-1'b), 2.25 (m, 2H, H-1b, H-5), 1.58–1.44 (m, 4H, H-4', H-5'), 1.42–1.23 (m, 4H, H-2', H-3') ppm; ^{13}C-NMR (75.5 MHz, $(CD_3)_2CO$) δ 158.2 (C-4´´´), 131.3 (C-1´´´), 129.8 (C-2´´´, C-6´´´), 113.6 (C-3´´´, C-5´´´), 79.4 (C-3), 71.7 (C-6´), 70.8 (C-1´´), 70.4 (C-4), 69.4 (C-2), 66.1 (C-5), 58.5 (C-6), 56.4 (C-1), 54.6 (OMe), 52.4 (C-1´), 35.2 (C-2´´), 29.6 (C-5´), 27.2 (C-2´), 26.0 (C-4´), 24.5 (C-3´) ppm; HRESI–MS calcd. for $C_{21}H_{36}NO_6$ ($[M+H]^+$): 398.2537, found: 398.2529.

***N*-{6'-[(2''-(3''',4'''-Dihydroxyphenyl)ethoxy]hexyl}-1-deoxynojirimycin (11d).** Compound **9d** (180 mg, 0.20 mmol) was used. Yield: (41 mg, 51%). R_f 0.30 (3:2 Et_2O–MeOH); $[\boldsymbol{\alpha}]_{\mathbf{D}}^{\mathbf{25}}$ –5 (*c* 0.94, CH_2Cl_2); ^{1}H-NMR (300 MHz, CD_3OD) δ 6.68 (brd, 1H, H-

5’’’), 6.67 (brd, H-2’’’), 6.53 (dd, 1H, $J_{5,6}$= 8.0 Hz, $J_{2,6}$= 2.1 Hz, H-6’’’), 3.94 (dd, 1H, $J_{5,6a}$= 2.6 Hz, $J_{6a,6b}$= 12.3 Hz, H-6a), 3.87 (dd, 1H, $J_{5,6b}$= 2.7 Hz, H-6b), 3.57 (t, 2H, $J_{H,H}$= 7.0 Hz, H-1’’), 3.63–3.52 (m, 1H, H-2), 3.45 (t, 1H, $J_{3,4}$= $J_{4,5}$= 9.3 Hz, H-4), 3.43 (t, 2H, $J_{5',6'}$= 6.3 Hz, H-6’), 3.25–3.13 (m , 2H, H-3, H-1a), 3.04–2.91 (m, 1H, H-1’a), 2.81–2.73 (m, 1H, H-1’b), 2.69 (t, 2H, H-2’’), 2.45 (t, 1H, $J_{1b,2}$= $J_{1a,1b}$= 11.1 Hz, H-1b), 2.45 (dt, 1H, H-5), 1.58–1.51 (m, 4H, H-4’, H-5’), 1.38–1.28 (m, 4H, H-2’, H-3’) ppm; ^{13}C-NMR (75.5 MHz, CD_3OD) δ 146.2 (C-3´´´), 144.6 (C-4´´´), 132.2 (C-1´´´), 121.3 (C-6´´´), 117.3 (C-2´´´), 116.4 (C-5´´´), 78.9 (C-3), 73.2 (C-6´), 71.7 (C-1´´), 69.9 (C-4), 68.7 (C-2), 67.5 (C-5), 56.5 (C-6), 55.7 (C-1), 54.1 (C-1´), 36.7 (C-2´´), 30.5 (C-5´), 27.7 (C-2´), 27.0 (C-4´), 24.6 (C-3´) ppm; HRESI–MS calcd. for $C_{20}H_{34}NO_7$ ($[M+H]^+$): 400.2330, found: 400.2335.

***N*-{6’-[2’’-(3’’’,4’’’-Dimethoxyphenyl)ethoxy]hexyl}-1-deoxynojirimycin (11e).** Compound **9e** (158 mg, 0.20 mmol) was used. Yield: 72 mg (84%). R_f 0.59 (5:2 CH_2Cl_2–MeOH); $[\alpha]_D^{25}$–5 (*c* 1.10, MeOH); ^{1}H-NMR (300 MHz, $(CD_3)_2CO$) δ 6.87 (d, 1H, $J_{2''',6'''}$= 1.6 Hz, H-2’’’), 6.83 (d, 1H, $J_{5''',6'''}$= 8.2 Hz, H-5’’’), 6.75 (dd, 1H, H-6’’’), 3.85 (m, 7H, 5OH, H-6a, H-6b), 3.78 (s, 3H, OMe), 3.76 (s, 3H, OMe), 3.56 (t, 1H, $J_{1'',2''}$= 7.0 Hz, H-1’’), 3.58–3.52 (m, 2H, H-2, H-4), 3.40 (m, 2H, H-6’), 3.24 (m, 1H, H-3), 3.08 (m, 1H, H-1a), 2.87 (m, 1H, H-1’a), 2.76 (t, 2H, H-2’’), 2.55 (m, 1H, H-1’b), 2.25 (m, 2H, H-1b, H-5), 1.58–1.47 (m, 4H, H-4’, H-5’), 1.42-1.28 (m, 4H, H-2’, H-3’) ppm; ^{13}C-NMR (75.5 MHz, $(CD_3)_2CO$) δ 149.3 (C-4´´´), 147.9 (C-3´´´), 132.1 (C-1´´´), 120.8 (C-6´´´), 113.1 (C-2´´´), 112.0 (C-5´´´), 79.3 (C-3), 71.7 (C-6´), 70.8 (C-1´´), 70.4 (C-4), 69.3 (C-2), 66.2 (C-5), 58.5 (C-6), 58.1 (C-1), 55.3 (OMe), 55.2 (OMe), 52.4 (C-1´), 35.7 (C-2´´), 29.7 (C-5’), 27.1 (C-2’), 26.0 (C-4’), 24.6 (C-3´) ppm; HRESI–MS calcd. for $C_{22}H_{38}NO_7$ ($[M+H]^+$): 428.2643, found: 428.2649.

***N*-{6’-[2’’-(4’’’-Hydroxy-3’’’-methoxyphenyl)ethoxy]hexyl}-1-deoxynojirimycin (11f).** Compound **9f** (173 mg, 0.20 mmol) was used. Yield: 75 mg (91%). R_f 0.56 (5:2 CH_2Cl_2–MeOH); $[\alpha]_D^{25}$–9 (*c* 0.87, MeOH); ^{1}H-NMR (300 MHz, CD_3OD) δ 6.80 (d, 1H, $J_{2''',6'''}$= 1.6 Hz, H-2’’’), 6.71 (d, 1H, $J_{5''',6'''}$= 8.0 Hz, H-5’’’), 6.64 (dd, 1H, H-6’’’), 3.86 (m, 2H, H-6a, H-6b), 3.83 (s, 3H, OMe), 3.60 (t, 2H, $J_{1'',2''}$= 6.9 Hz, H-1´´), 3.50 (m, 1H, H-2), 3.44 (t, 2H, $J_{5',6'}$= 6.4 Hz, H-6´), 3.38 (m, 1H, H-4), 3.16 (t, 1H, $J_{2,3}$= $J_{3,4}$= 9.2 Hz, H-3), 3.03 (dd, 1H, $J_{1a,2}$= 4.8 Hz, $J_{1a,1b}$= 11.2 Hz, H-1a), 2.84 (m, 1H, H-1’a), 2.76 (t, 2H, H-2’’), 2.61 (m, 1H, H-1’b), 2.25 (t, 1H, $J_{1b,2}$= 10.9 Hz, H-1b), 2.20 (dt. 1H, $J_{4,5}$= 8.4 Hz, $J_{5,6a}$ = $J_{5,6b}$= 2.6 Hz, H-5), 1.60–1.44 (m, 4H, H-4’, H-5’), 1.40–1.31 (m, 4H, H-2’, H-3’) ppm; ^{13}C-NMR (75.5 MHz, CD_3OD) δ 148.7 (C-3’’’), 145.8 (C-4’’’), 132.0 (C-1’’’), 122.3 (C-6’’’), 116.1 (C-5’’’), 113.7 (C-2’’’), 80.4 (C-3), 73.2 (C-6´), 71.8 (C-1´´), 70.5 (C-4), 67.3 (C-2), 60.1 (C-5), 59.1 (C-6), 57.5 (C-1), 56.4 (OMe), 53.7 (C-1´), 36.8 (C-2´´), 30.6 (C-5´), 28.3 (C-2´), 27.2 (C-4´), 25.1 (C-3´) ppm; HRESI-MS calcd. for $C_{21}H_{36}NO_7$ ($[M+H]^+$): 414.2486, found: 414.2491.

***N*-{6’-[2’’-(3’’’,4’’’-Methylenedioxyphenyl)ethoxy]hexyl}-1-deoxynojirimycin (11g).** Compound **9g** (154 mg, 0.20 mmol) was used. Yield: 38 mg (46%). R_f 0.70 (5:2 CH_2Cl_2–MeOH); $[\alpha]_D^{25}$–8 (*c* 1.74, MeOH); ^{1}H-NMR (300 MHz, $(CD_3)_2CO$) δ 6.78 (d, 1H, $J_{2''',3'''}$= 1.2 Hz, H-2’’’), 6.72 (d, 1H, $J_{5''',6'''}$= 7.8 Hz, H-5’’’), 6.69 (dd, 1H, H-6’’’), 5.93 (s, 2H, OCH_2O), 3.91 (m, 6H, 4OH, H-6a, H-6b), 3.54 (t, 2H, 3.54 (t, 2H, $J_{1'',2''}$= 6.9 Hz, H-1’’), 3.40 (t, 2H, $J_{5',6'}$= 6.4 Hz, H-6’), 3.56–3.37 (m, 2H, H-2, H-4), 3.19 (m, 1H, H-3), 3.02 (m, 1H, H-1a), 2.85–273 (m, 1H, H-1’a), 2.75 (m, 2H, H-2’’), 2.48 (m, 1H, H-1’b), 2.19–2.13 (m, 2H, H-1b, H-5), 1.58–1.40 (m, 4H, H-3’, H-5’), 1.40–1.12 (m, 4H, H-2’, H-4’) ppm; ^{13}C-NMR (75.5 MHz, $(CD_3)_2CO$) δ 148.4 (C-4´´´), 146.7 (C-3´´´), 134.2 (C-1´´´), 122.6 (C-6´´´), 110.1 (C-2´´´), 108.7 (C-5´´´), 101.6 (OCH_2O), 80.4 (C-3), 72.4 (C-6´), 71.9 (C-1´´), 71.2 (C-4), 70.4 (C-2), 67.0 (C-5), 59.3 (C-6), 57.4

(C-1), 53.2 (C-1´), 36.6 (C-2´´), 30.5 (C-5´), 28.0 (C-2´), 26.9 (C-4´), 25.6 (C-3´) ppm; HRESI–MS calcd. for $C_{21}H_{34}NO_7$ ($[M+H]^+$): 412.2330, found: 412.2336.

***N*-[5'-(2''-Phenylethoxy)pentyl]-1-deoxynojirimycin (12a).** Compound **10a** (143 mg, 0.20 mmol) was used. Yield: 71 mg (quant.). R_f 0.48 (5:2 CH_2Cl_2–MeOH); $[\alpha]_D^{25}$–3 (*c* 0.92, CH_2Cl_2); ^{1}H NMR (300 MHz, CD_3OD) δ 7.29–7.15 (m, 5H, Ar-H), 4.10 (m, 1H, H-6a), 3.94 (m, 1H, H-6b), 3.78 (m, 1H, H-2), 3.67–3.61 (m, 4H, H-1'', H-5'), 3.52–3.42 (m, 3H, H-3, H-4, H-1a), 3.21–2.99 (m, 4H, H-1'a, H-1'b, H-2''), 2.89–2.82 (m, 2H, H-1b, H-5), 1.87–1.36 (m, 6H, H-2', H-3', H-4') ppm; ^{13}C-NMR (75.7 MHz, CD_3OD) δ 140.4 (C-1´´´), 129.9 (C-2´´´, C-6´´´), 129.3 (C-3´´´, 5´´´), 127.1 (C-4´´´), 77.9 (C-3), 72.8 (C-1''), 71.3 (C-5´), 68.8 (C-4), 67.8 (C-2), 67.4 (C-5), 55.3 (C-6), 54.7 (C-1), 54.1 (C-1´), 37.2 (C-2´´), 29.9 (C-4´), 24.3 (C-2'), 24.0 (C-3´) ppm; HRESI–MS calcd. for $C_{19}H_{32}NO_5$ ($[M+H]^+$): 354.2275, found: 354.2274.

***N*-{5'-[2''-(p-Methoxyphenyl)ethoxy]pentyl}-1-deoxynojirimycin (12c).** Compound **10c** (149 mg, 0.20 mmol) was used. Yield: 77 mg (quant.). R_f 0.43 (5:2 CH_2Cl_2–MeOH); $[\alpha]_D^{25}$–1 (*c* 1.13, CH_2Cl_2); ^{1}H-NMR (300 MHz, CD_3OD) δ 7.13 (m, 2H, Ar-H), 6.83 (m, 2H, Ar-H), 4.07 (brd, 1H, $J_{6a,6b}$= 12.3 Hz, H-6a), 3.90 (brd, 1H, H-6b), 3.75 (s, 3H, OMe), 3.70 (m, 1H, H-2), 3.60 (t, 2H, $J_{1'',2''}$ = 3.9 Hz, H-1''), 3.62–3.58 (m, 1H, H-4), 3.46 (t, 2H, $J_{4',5'}$ = 6.0 Hz, H-5'), 3.37–3.11 (m, 4H, H-3, H-1a, H-1'a, H-1'b), 2.96–2.84 (m, 2H, H-1b, H-5), 2.78 (t, 2H, H-2''), 1.71–1.57 (m, 4H, H-2', H-4'), 1.45–1.36 (m, 2H, H-3') ppm; ^{13}C-NMR (75.7 MHz, CD_3OD) δ 159.5 (C-4´´´), 132.4 (C-1´´´), 130.9 (C-2´´´, C-6´´´), 114.8 (C-3´´´, C-5´´´), 78.4 (C-3), 73.1 (C-1''), 71.4 (C-5´), 69.2 (C-4), 68.1 (C-2), 67.4 (C-5), 55.8 (C-6), 55.7 (OMe), 55.1 (C-1), 54.0 (C-1'), 36.3 (C-2´´), 30.1 (C-4´), 24.5 (C-2´), 24.0 (C-3´) ppm; HRESI–MS calcd. for $C_{20}H_{34}NO_6$ ($[M+H]^+$): 384.2381, found: 384.2376.

***N*-{5’-[2’’-(3’’’,4’’’-dihydroxyphenyl)ethoxy]pentyl}-1-deoxynojirimycin (12d).** Compound **10d** (185 mg, 0.20 mmol) was used. Yield: 25 mg (32%). R_f 0.29 (5:2 CH_2Cl_2–MeOH); $[\alpha]_D^{25}$ –7 (*c* 0.53, CH_2Cl_2); ^{1}H-NMR (300 MHz, CD_3OD) δ 6.71 (brd, 1H, $J_{5''',6'''}$=8.0 Hz, H-5’’’), 6.69 (brs, 1H, H-2’’’), 6.55 (dd, $J_{2''',6'''}$= 1.9 Hz, H-6’’’), 3.96–3.85 (m, 2H, H-6a, H-6b), 3.80–3.66 (m, 1H, H-2), 3.60 (t, 2H, $J_{1'',2''}$= 7.1 Hz, H-1’’), 3.64–3.53 (m, 1H, H-4), 3.46 (t, 2H, $J_{4',5'}$= 6.4 Hz, H-5’), 3.24 (t, 1H, $J_{2,3}$= $J_{3,4}$= 9.1 Hz, H-3), 3.18–3.09 (m, 2H, H-1a, H-1’a), 2.92 (m, 1H, H-1’b), 2.71 (t, 2H, H-2’’), 2.39 (t, 1H, $J_{1b,2}$= $J_{1a,1b}$= 11.3 Hz, H-1b), 2.37 (m, 1H, H-5), 1.64–1.50 (m, 4H, H-2’, H-4’), 1.30–1.30 (m, 2H, H-3’) ppm; HRESI-MS calcd. for $C_{19}H_{32}NO_7$ ($[M+H]^+$): 386.2173, found: 386.2169.

Table S1. Inhibitory profile of compounds 11,12 (IC_{50}, K_i in µM)

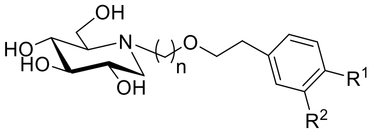

Comp.	Enzyme						
	**α-Glucosidase (*Saccharomyces cerevisiae*)**	**β-Glucosidase (almonds)**	**α-Galactosidase (green coffee beans)**	**β-Galactosidase (*Asp. Oryzae*)**	**β-Galactosidase (*E. coli*)**	**AChE (*Electrophorus electricus)***	**BuChE (equine serum)**
**11a**<br>n = 6<br>R^1=R^2=H	IC_{50} = 126<br><br>K_{ia} = 93 ± 32<br>(Competitive)	**IC_{50}= 4.6**<br><br>**K_{ia} = 6.5 ± 0.1**<br>(Competitive)	IC_{50} >100	IC_{50} >100	IC_{50} >100	IC_{50} >100	IC_{50} >100
**11b**<br>n = 6<br>R^1=OH, R^2=H	IC_{50} >100	IC_{50} = 12<br><br>K_{ia} = 17 ± 5<br>(Competitive)	IC_{50} >100	IC_{50} >100	IC_{50} >100	IC_{50} >100	IC_{50} >100
**11c**<br>n = 6<br>R^1=OMe, R^2=H	IC_{50} >100	**IC_{50} = 6.0**<br><br>**K_{ia} = 4.5 ± 0.7**<br>(Competitive)	IC_{50} >100	IC_{50} >100	IC_{50} >100	IC_{50} >100	IC_{50} >100
**11d**<br>n = 6<br>R^1=R^2=OH	IC_{50} >100	IC_{50} = 15<br><br>K_{ia} = 11 ± 4<br>(Competitive)	IC_{50} >100	IC_{50} >100	IC_{50} >100	IC_{50} >100	IC_{50}= 76
**11e**<br>n = 6<br>R^1=R^2=OMe	IC_{50} >100	IC_{50} = 14<br><br>K_{ia} = 15 ± 3<br>(Competitive)	IC_{50} >100	IC_{50} >100	IC_{50} >100	**IC_{50}= 5.8**<br><br>**K_{ia}= 9.3 ± 1.2**<br>**K_{ib}= 5.2 ± 0.5**<br>(Mixed)	**IC_{50} = 1.9**<br><br>**K_{ia}= 1.4 ± 0.4**<br>**K_{ib}= 4.5 ± 1.4**<br>(Mixed)
**11f**<br>n = 6<br>R^1=OH, R^2=OMe	IC_{50} >100	**IC_{50} = 8.2**<br><br>**K_{ia} = 9.6 ± 0.8**<br>(Competitive)	IC_{50} >100	IC_{50} >100	IC_{50} >100	**IC_{50}= 7.3**<br><br>K_{ia}= K_{ib}= 17 ± 5<br>(Non-competitive)	**IC_{50} = 1.8**<br><br>**K_{ia}= 1.5 ± 0.2**<br>**K_{ib}= 5.0 ± 2.0**<br>(Mixed)
**11g**<br>n = 6<br>R^1,R^2=<br>O-CH_2-O	IC_{50} >100	**IC_{50}= 8.4**<br><br>**K_{ia} = 9.0 ± 3.0**<br>(Competitive)	IC_{50} >100	IC_{50} >100	IC_{50} >100	IC_{50} = 48<br><br>K_{ia}= 56 ± 10<br>K_{ib}= 43 ± 14<br>(Mixed)	**IC_{50} = 7.3**<br><br>**K_{ia}= K_{ib} =9.2 ± 1.9**<br>(Non-competitive)
**12a**	IC_{50} >100	IC_{50} = 28	IC_{50} >100	IC_{50} >100	IC_{50} >100	IC_{50} >100	IC_{50} >100

n = 5<br>R^1=R^2=H		K_{ia} = 23 ± 4<br>(Competitive)					
**12c**<br>n = 5<br>R^1=OMe, R^2=H	IC_{50} >100	IC_{50} = 25<br><br>K_{ia} = 14 ± 6<br>(Competitive)	IC_{50} >100	IC_{50} >100	IC_{50} >100	IC_{50} >100	IC_{50} >100
**12d**<br>n = 5<br>R^1=R^2=OH	IC_{50} >100	IC_{50} >50	N.T.	N.T.	N.T.	IC_{50} >100	IC_{50} >100
**1-DNJ**	IC_{50} = 35	IC_{50} = 71	IC_{50} = 16	IC_{50} >100	IC_{50} >100	IC_{50} >100	IC_{50}= 10<br><br>K_{ia}= K_{ib}= 16 ± 3<br>(Non-competitive)

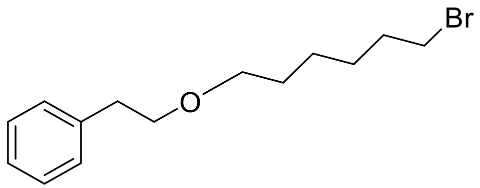

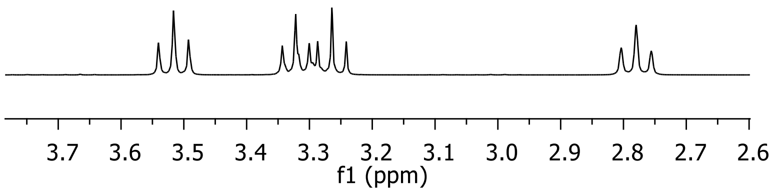

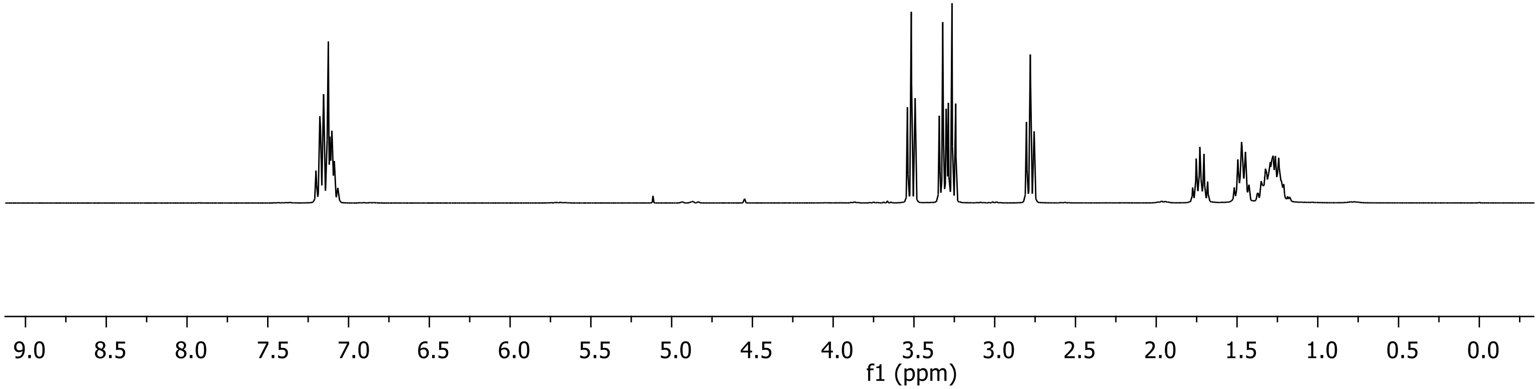

^{1}H-NMR (300 MHz, $CDCl_3$) of **7a**

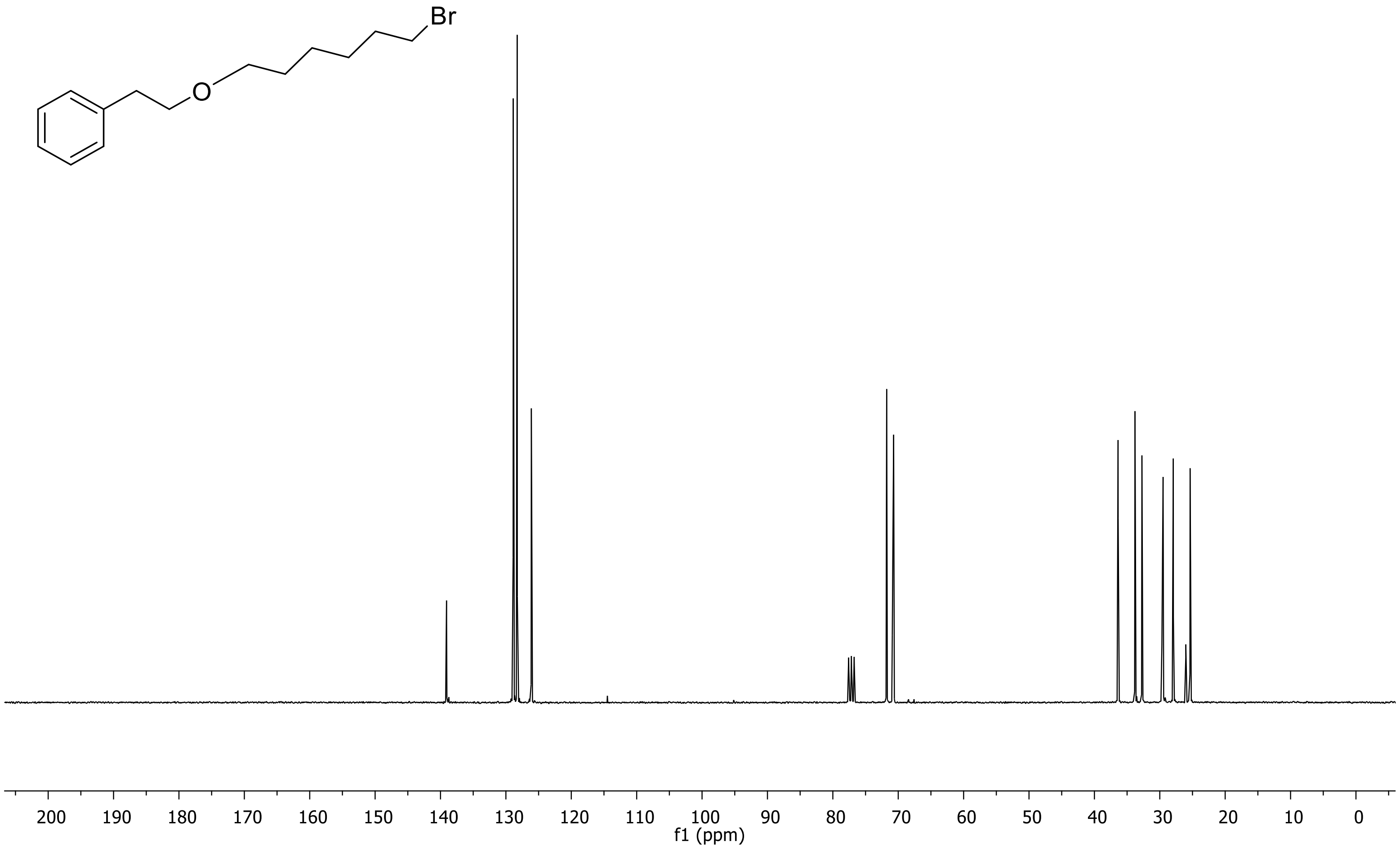

^{13}C-NMR (75.5 MHz, $CDCl_3$) of **7a**

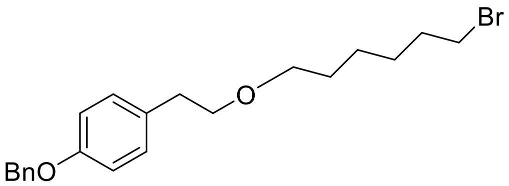

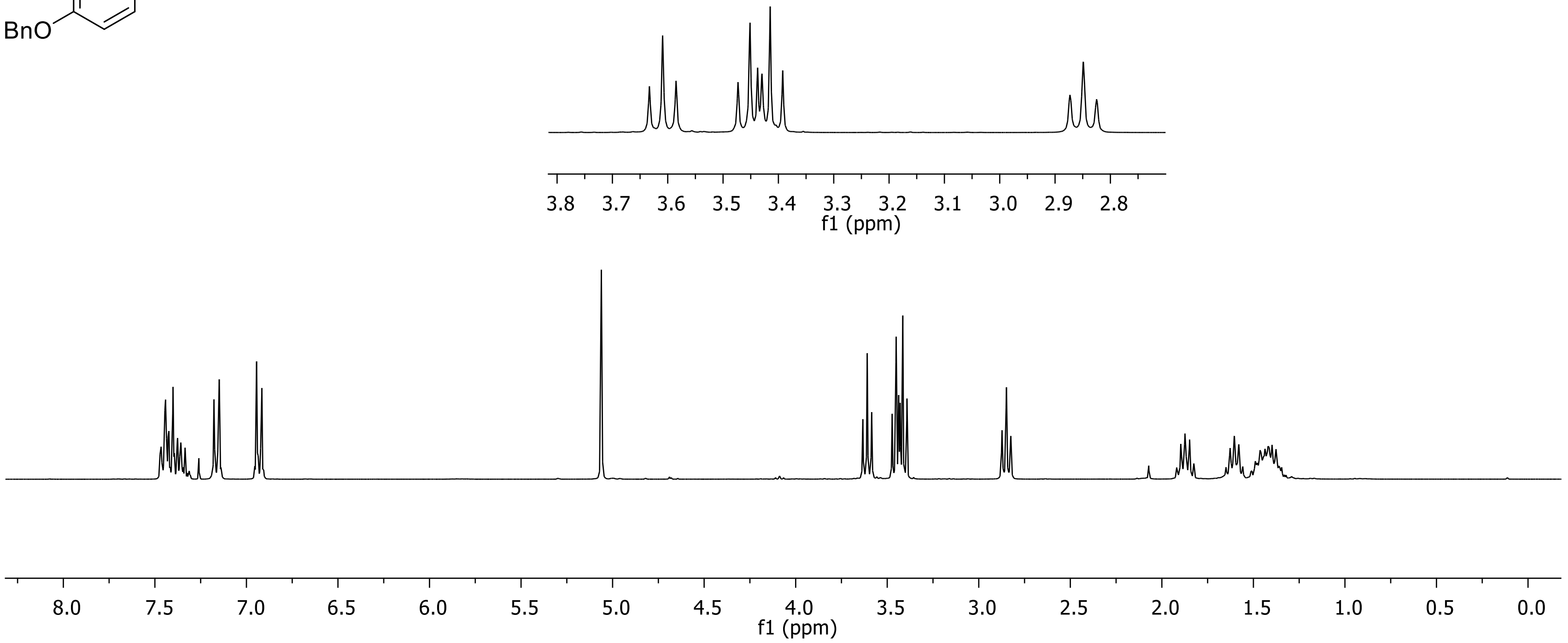

^{1}H-NMR (300 MHz, $CDCl_3$) of **7b**

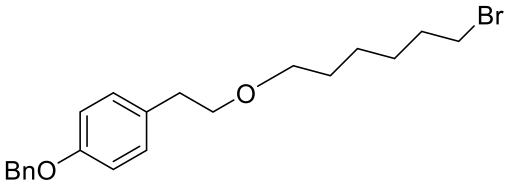

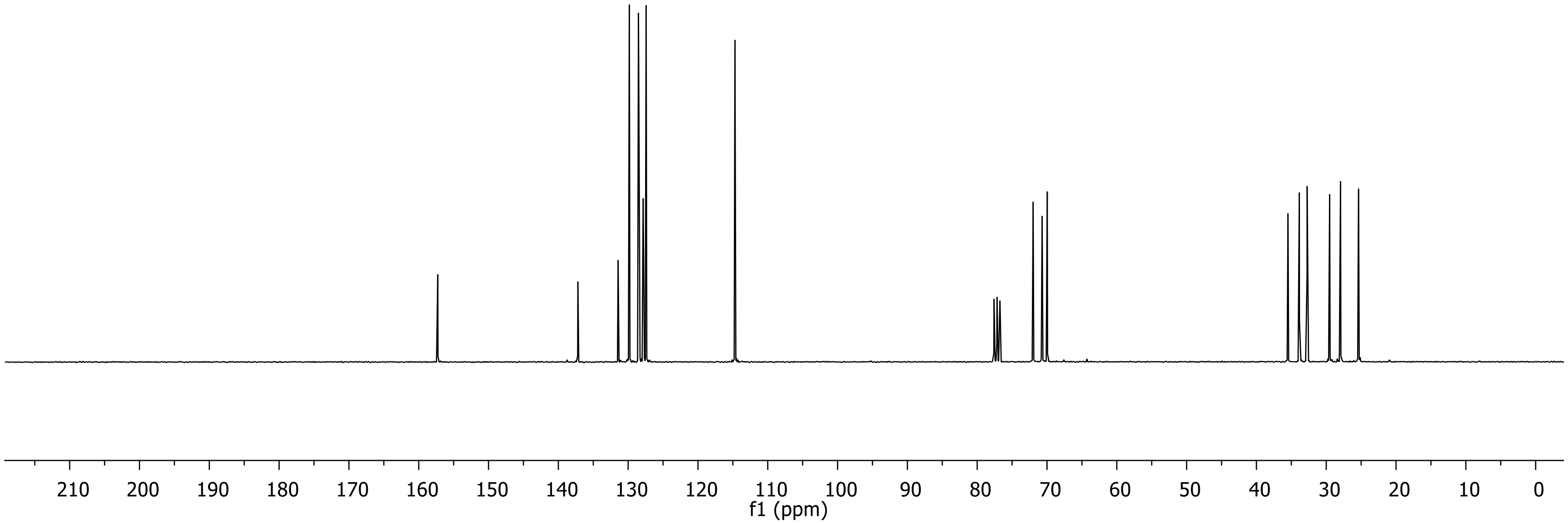

^{13}C-NMR (75.5 MHz, $CDCl_3$) of **7b**

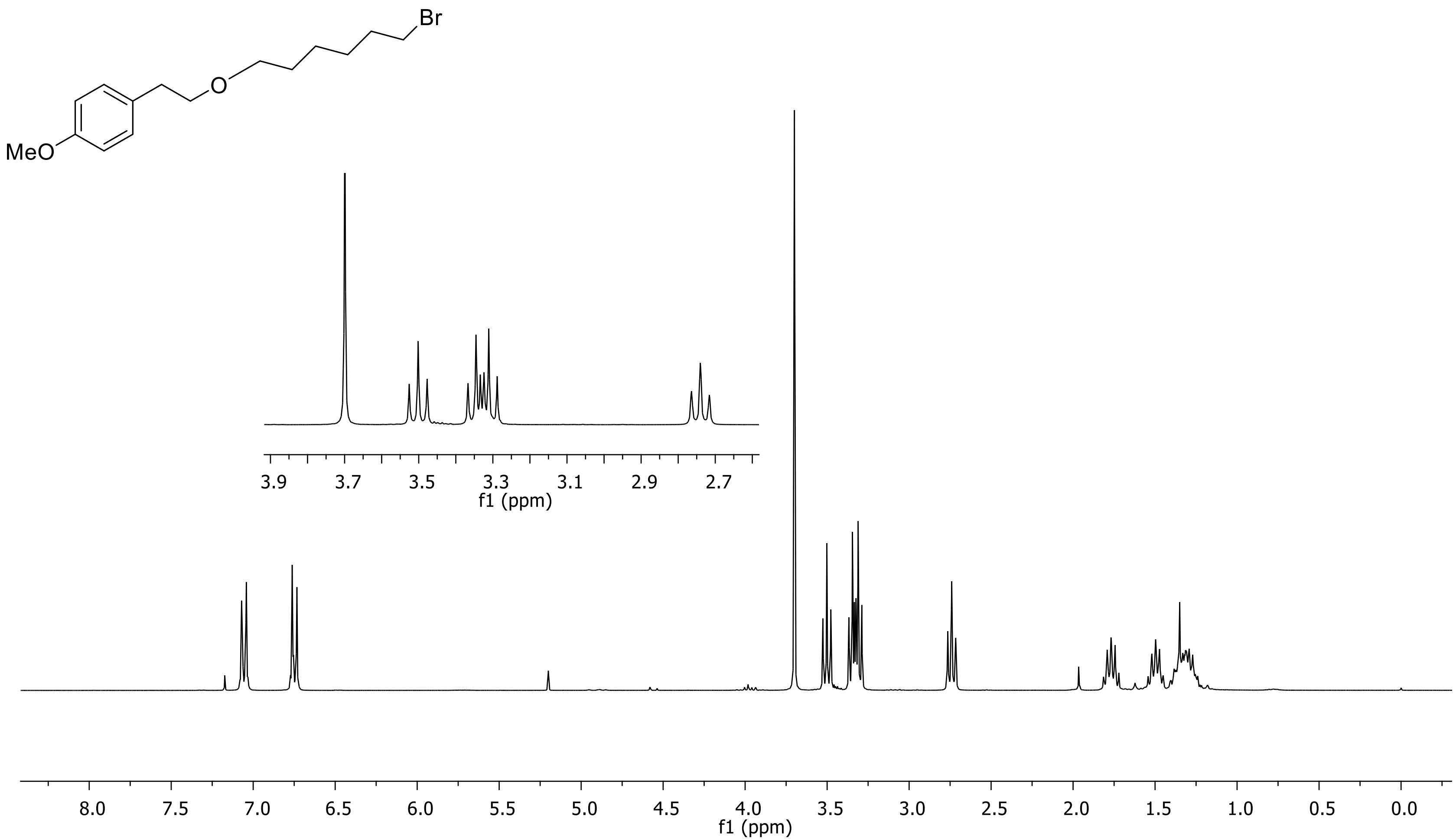

^{1}H-NMR (300 MHz, $CDCl_3$) of **7c**

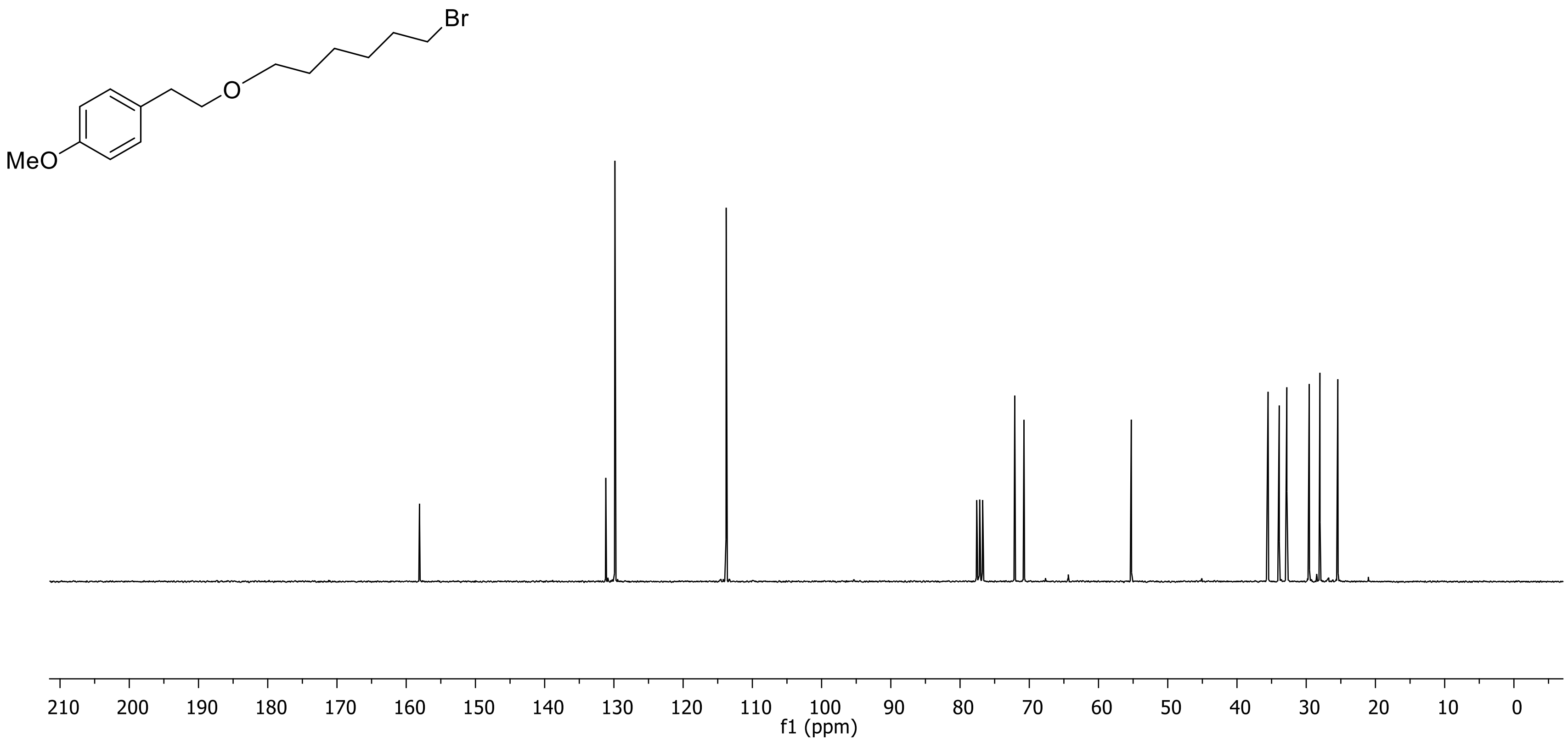

^{13}C-NMR (75.5 MHz, $CDCl_3$) of **7c**

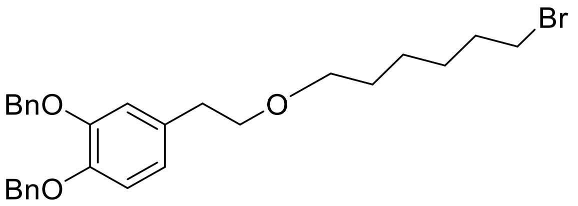

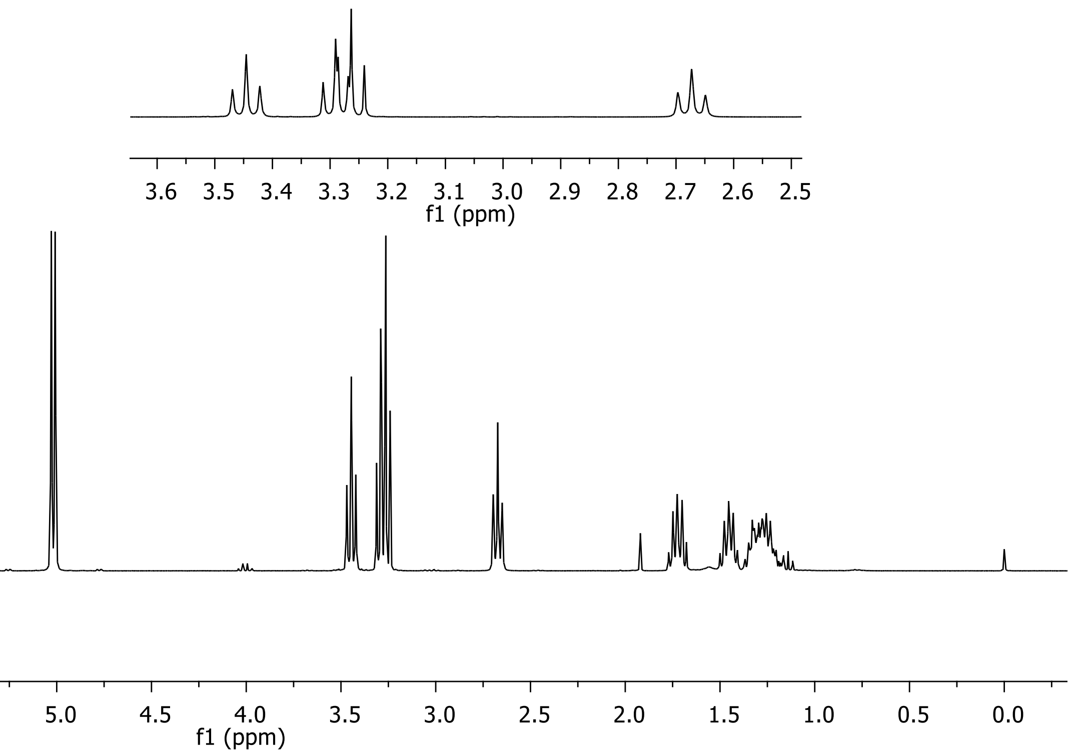

^{1}H-NMR (300 MHz, $CDCl_3$) of **7d**

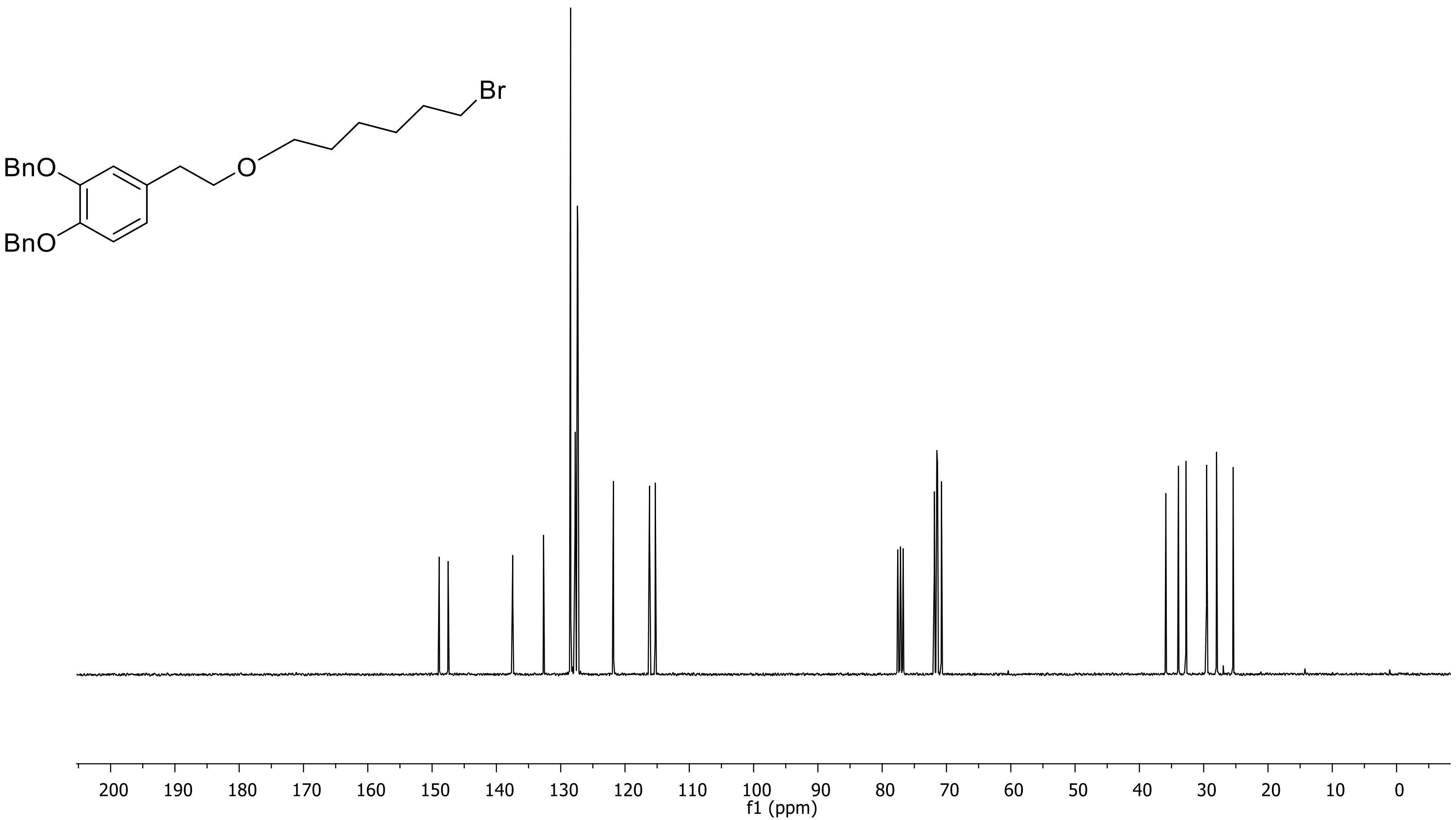

^{13}C-NMR (75.5 MHz, $CDCl_3$) of **7d**

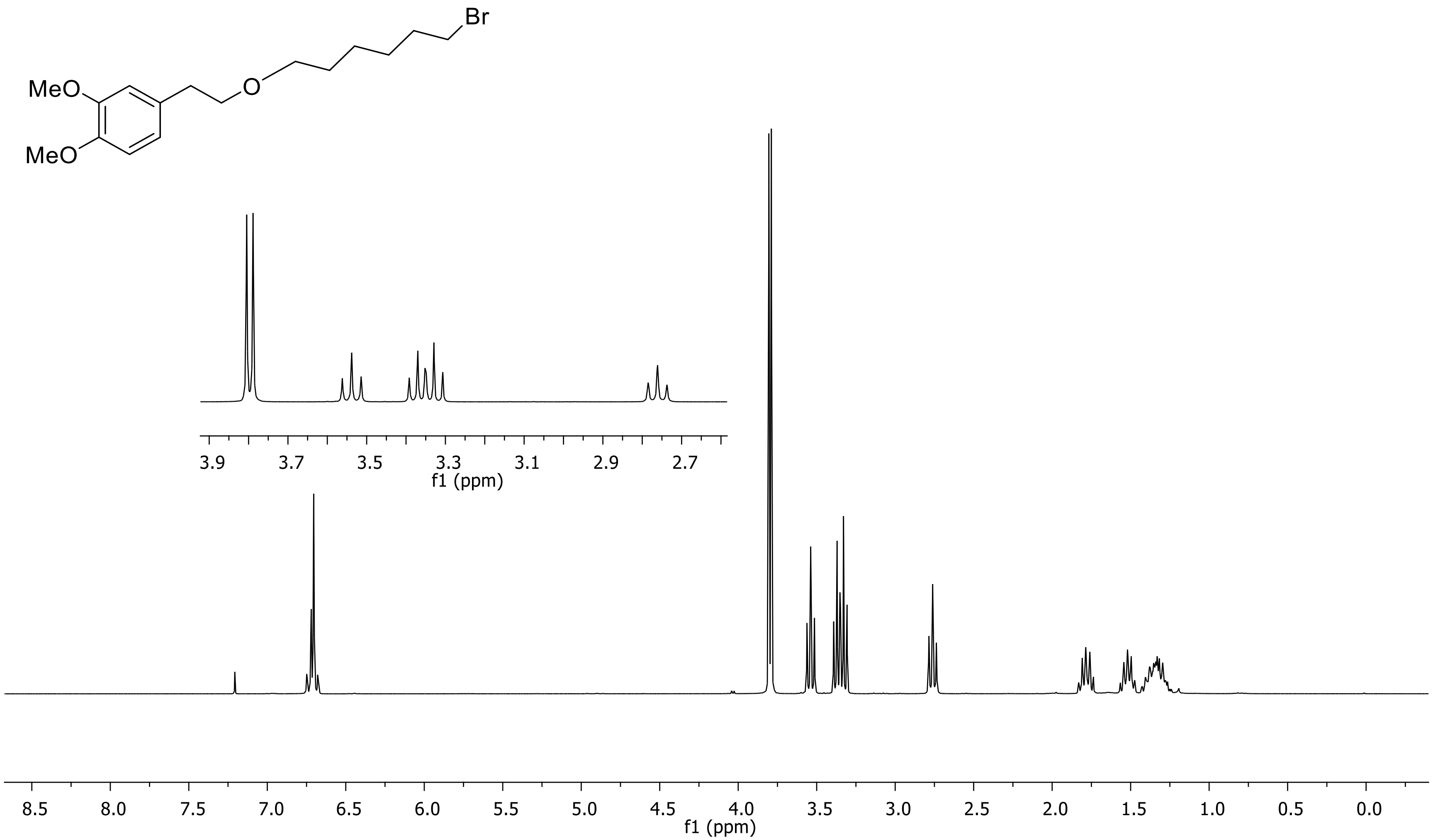

^{1}H-NMR (300 MHz, $CDCl_3$) of **7e**

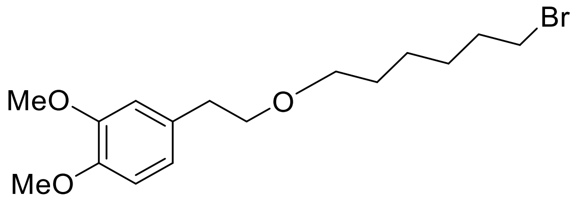

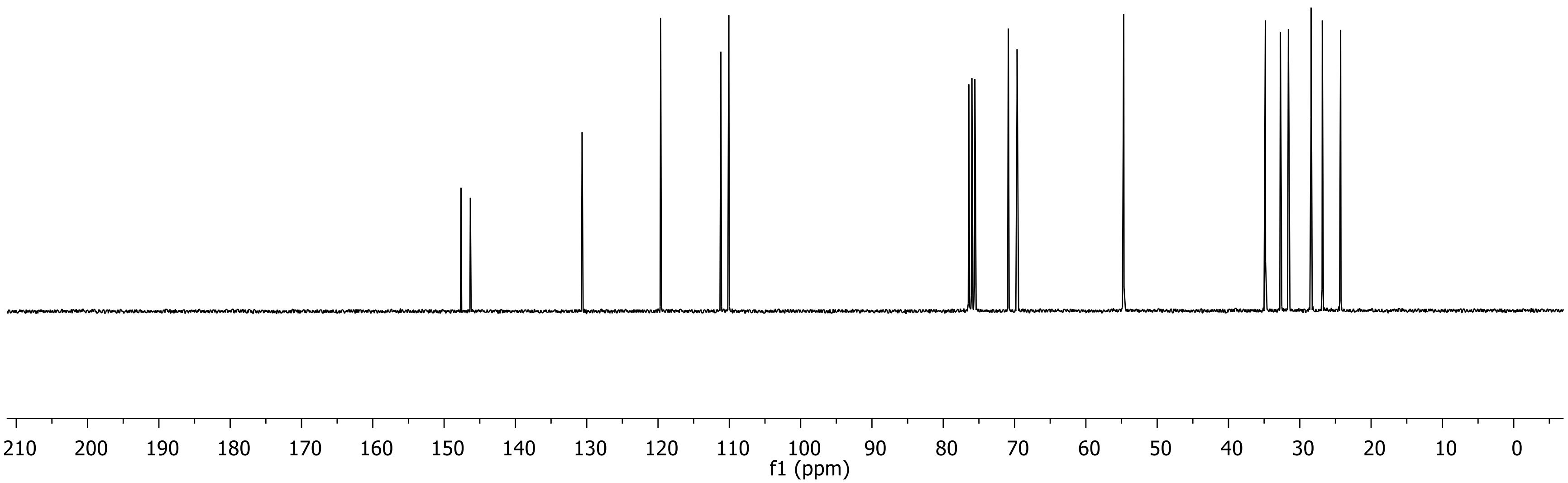

^{13}C-NMR (75.5 MHz, $CDCl_3$) of **7e**

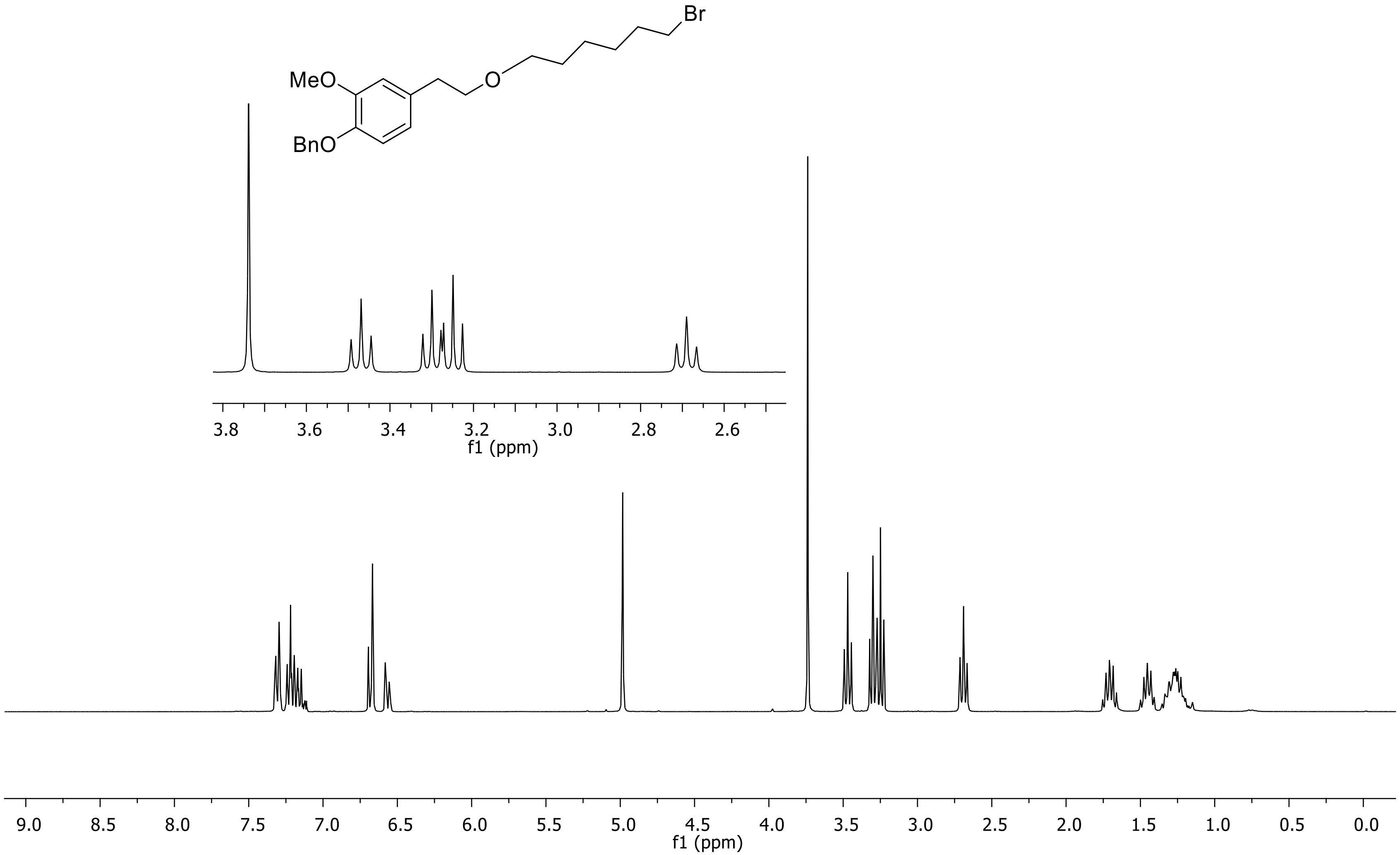

^{1}H-NMR (300 MHz, $CDCl_3$) of **7f**

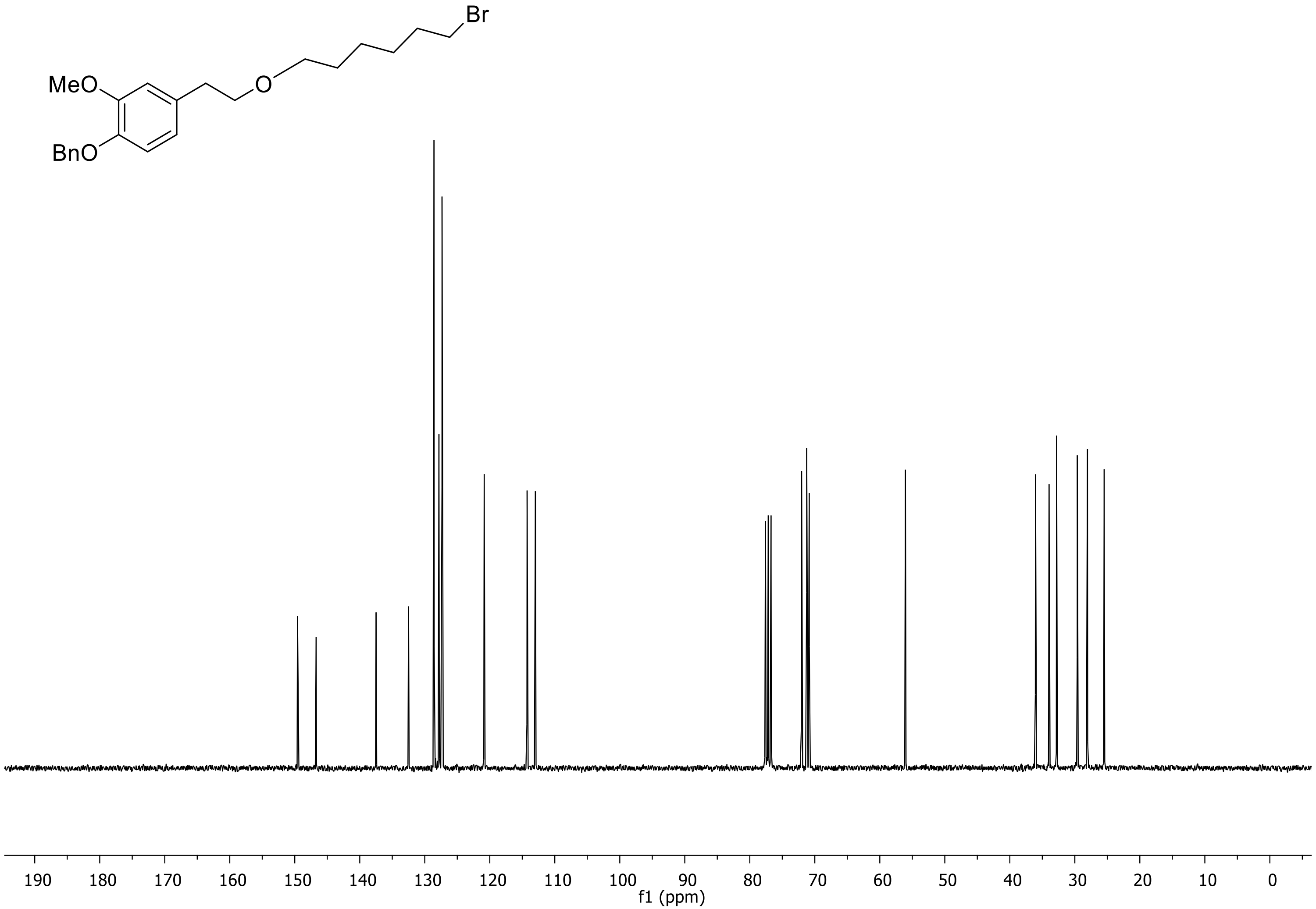

^{13}C-NMR (75.5 MHz, $CDCl_3$) of **7f**

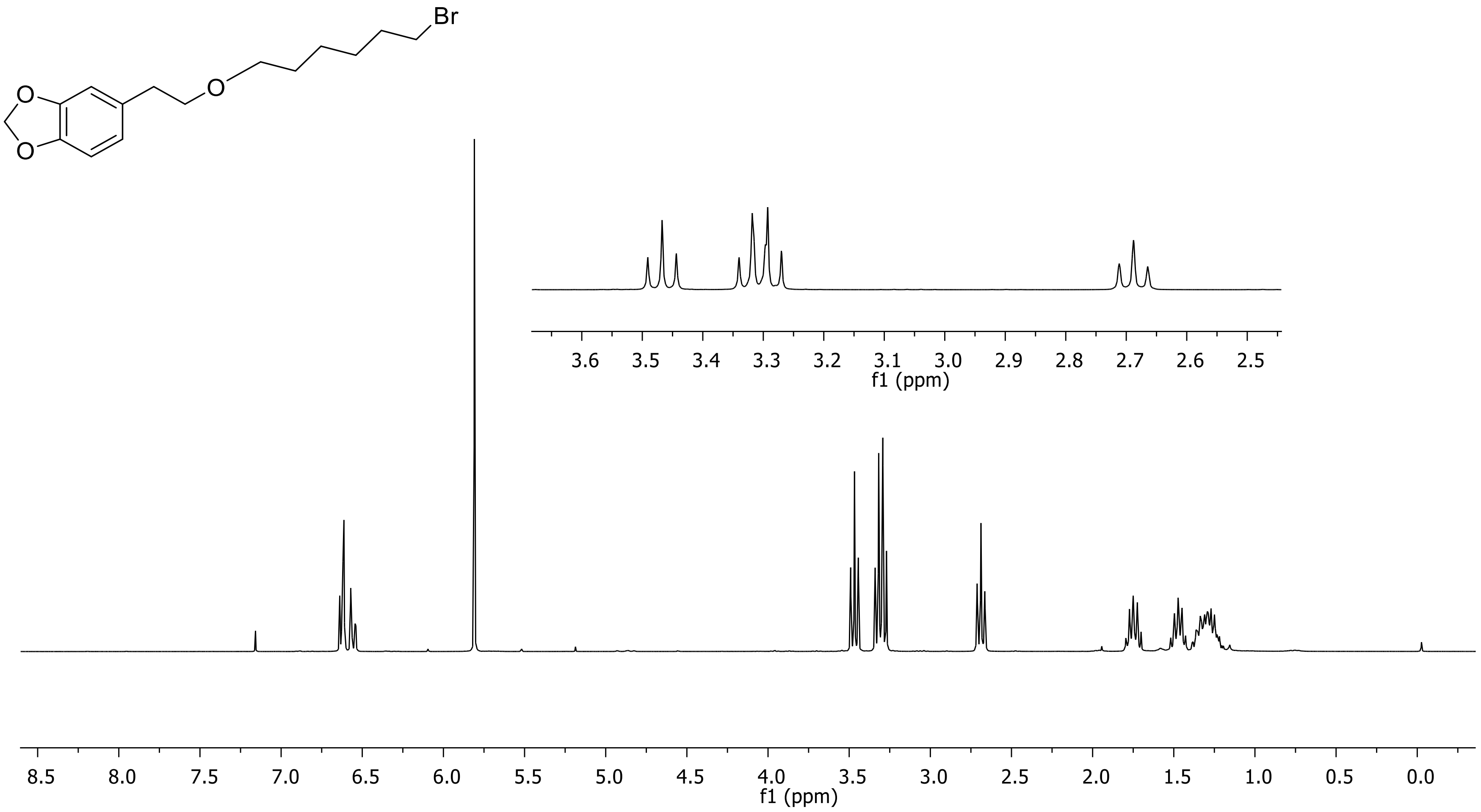

^{1}H-NMR (300 MHz, $CDCl_3$) of **7g**

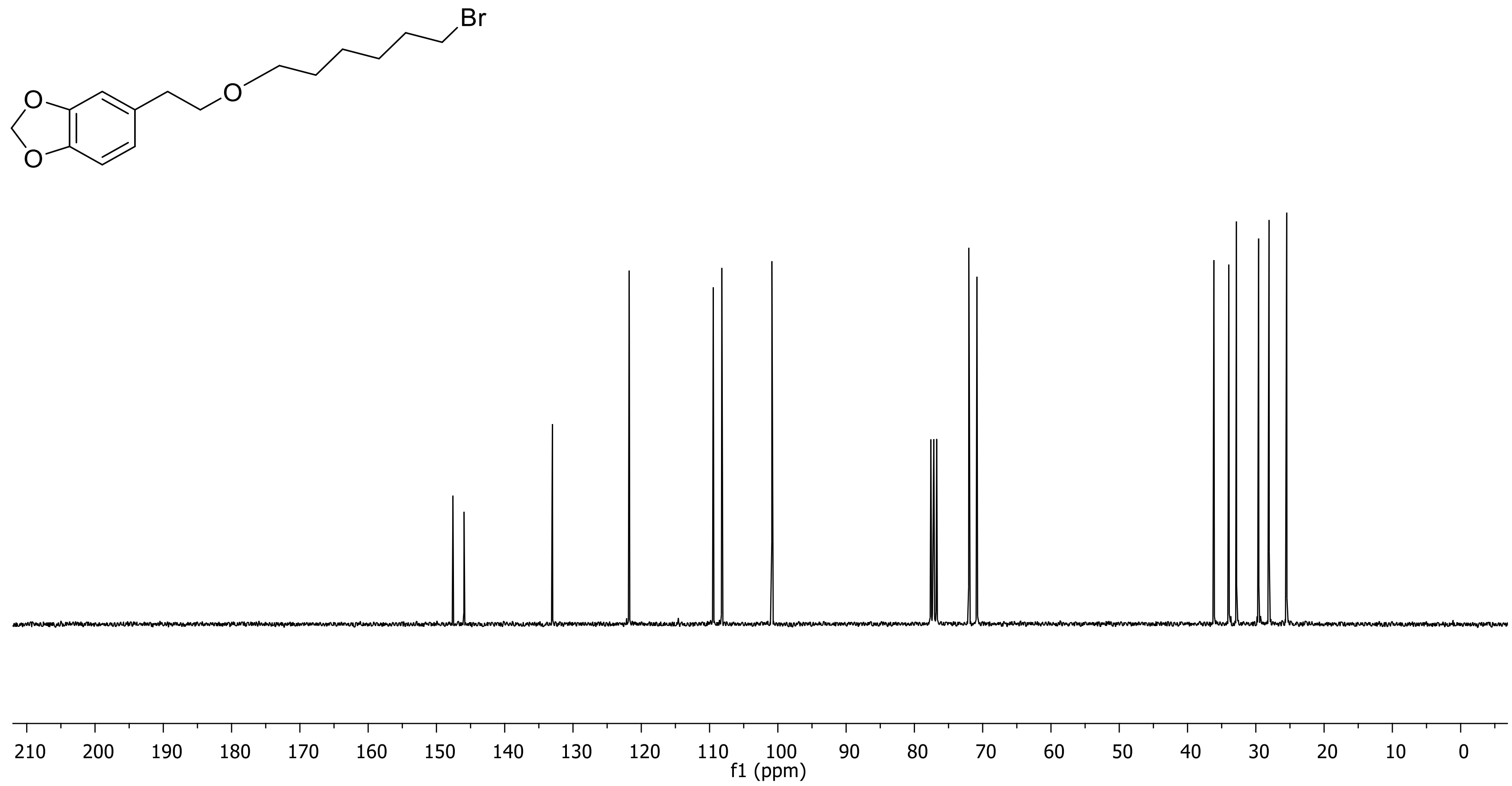

^{13}C-NMR (75.5 MHz, $CDCl_3$) of **7g**

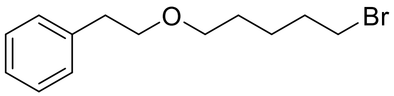

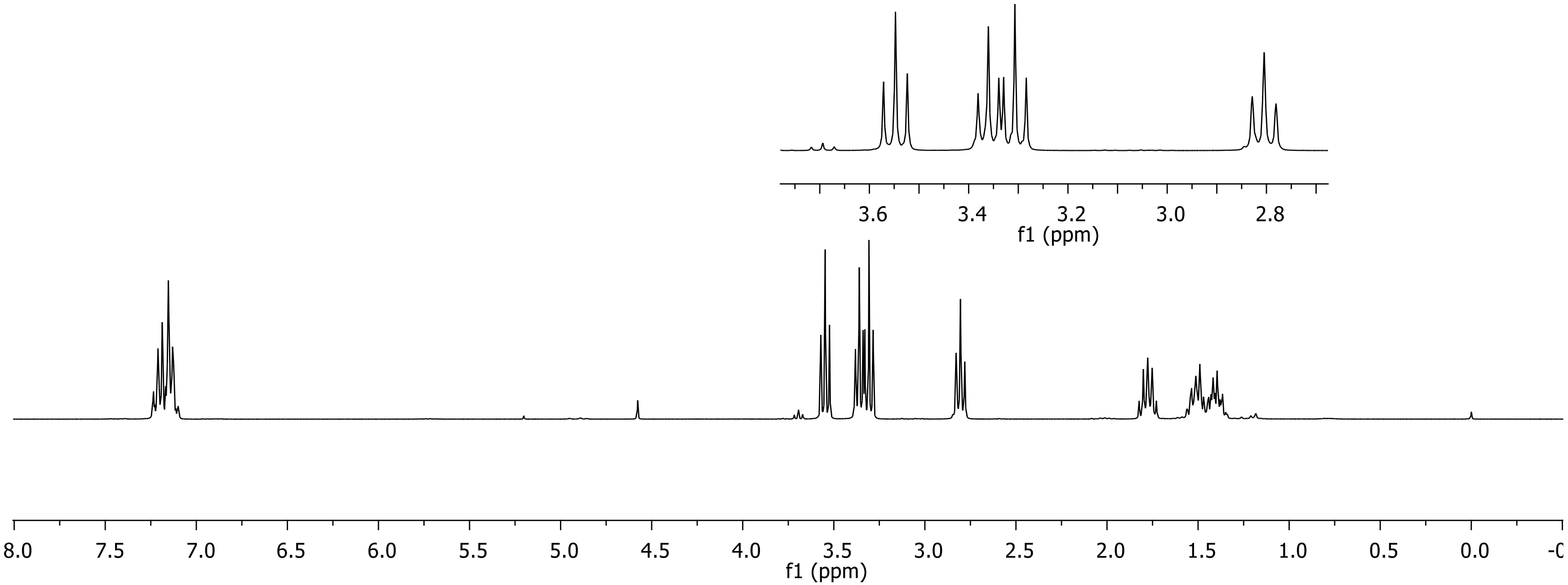

^{1}H-NMR (300 MHz, $CDCl_3$) of **8a**

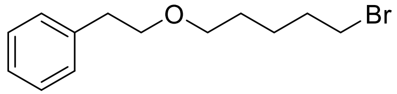

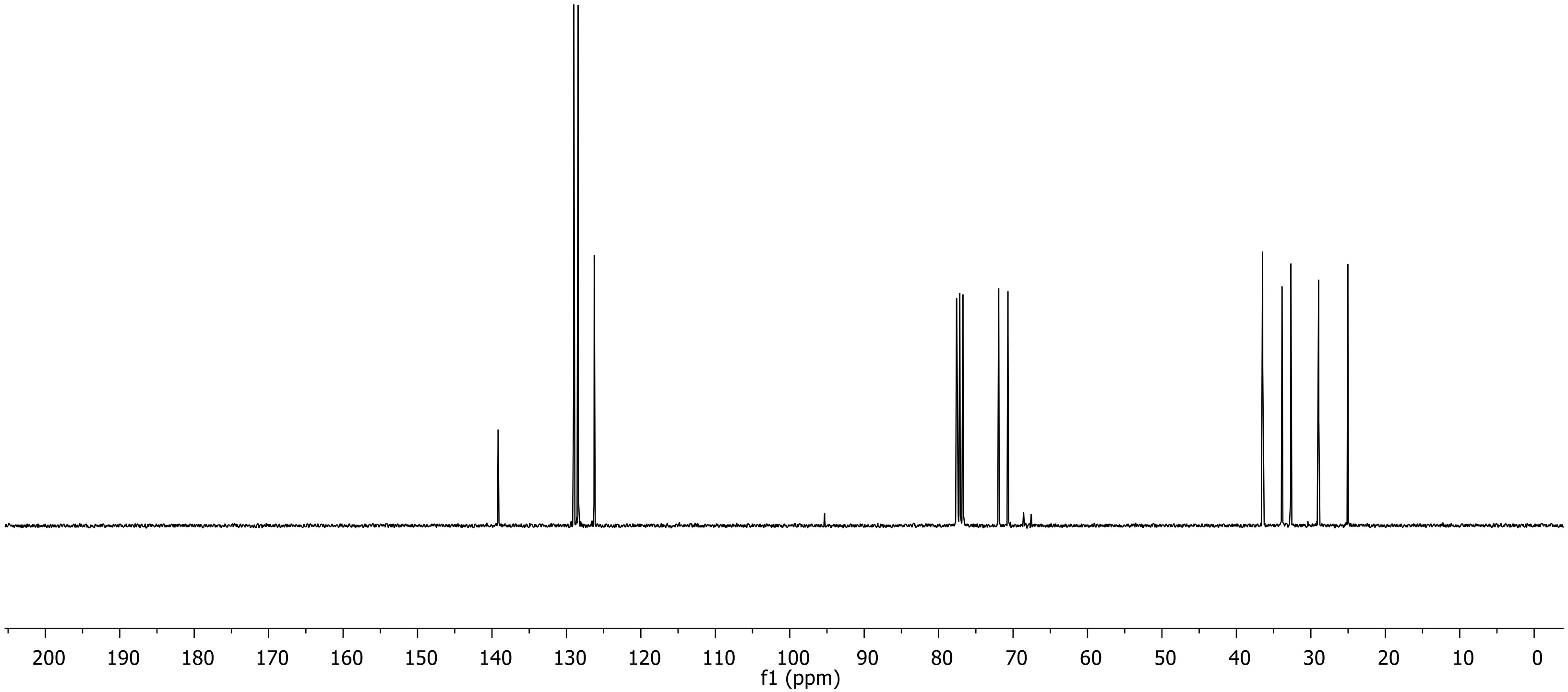

^{13}C-NMR (75.5 MHz, $CDCl_3$) of **8a**

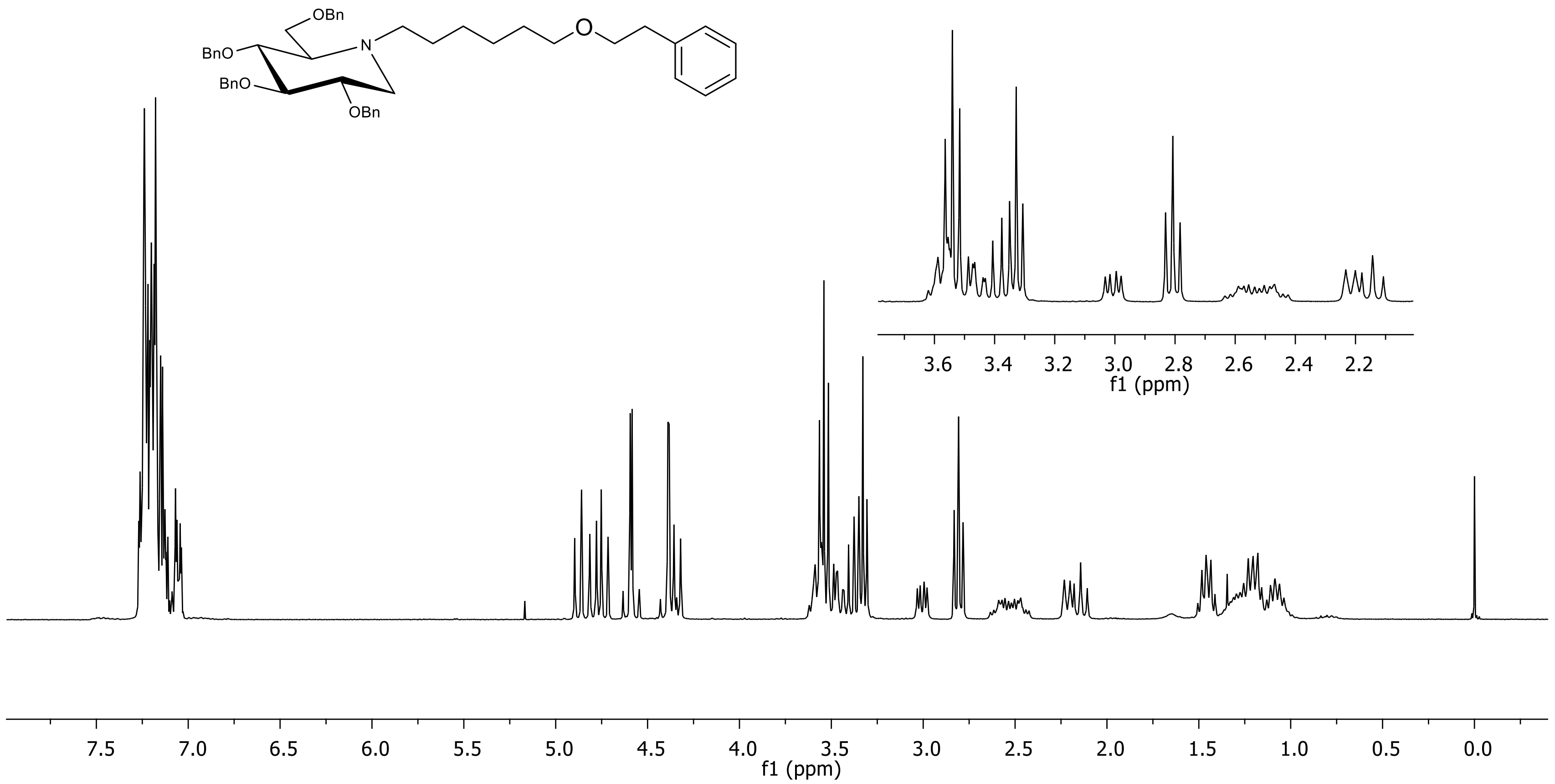

^{1}H-NMR (300 MHz, $CDCl_3$) of **9a**

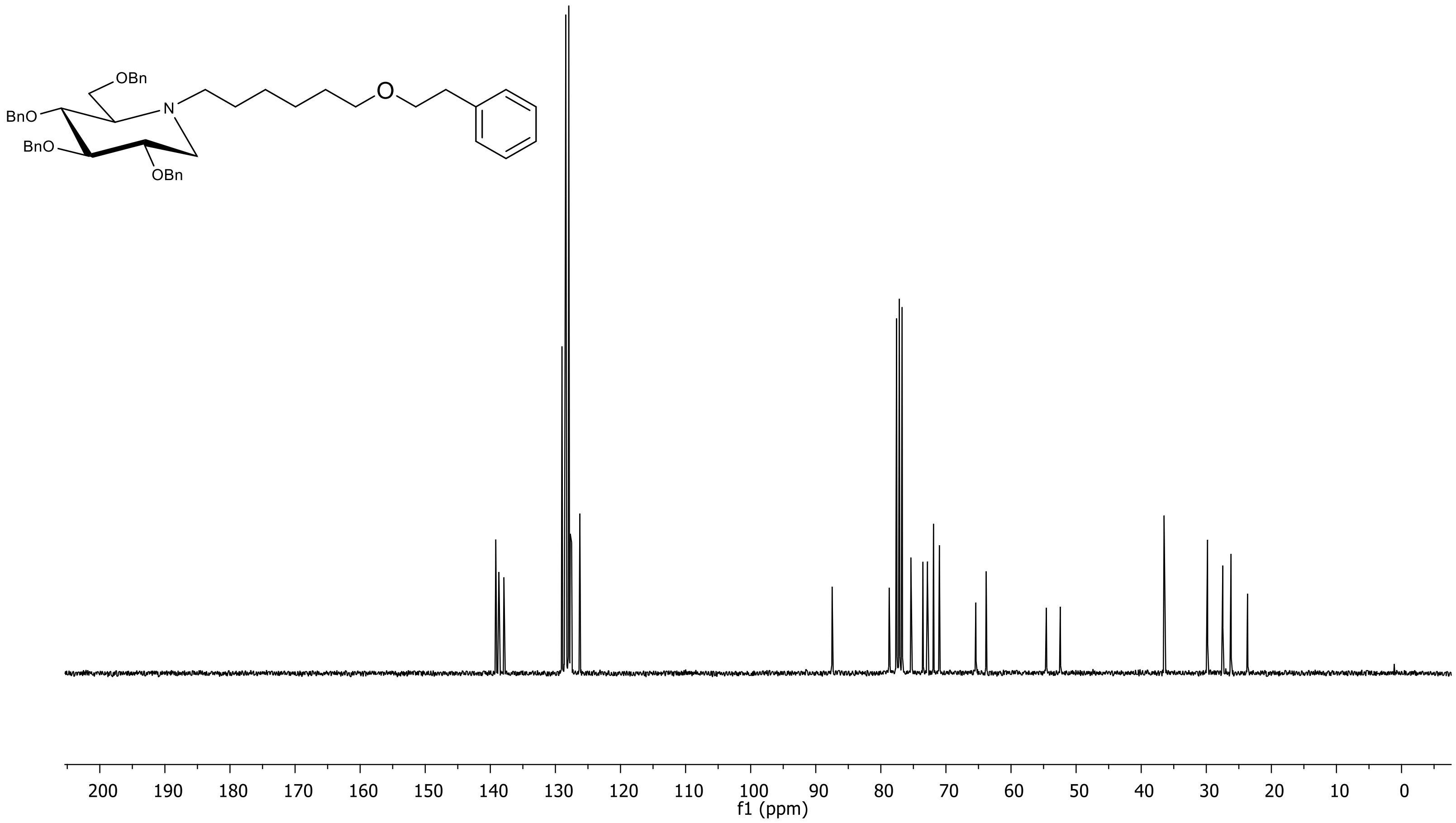

^{13}C-NMR (75.5 MHz, $CDCl_3$) of **9a**

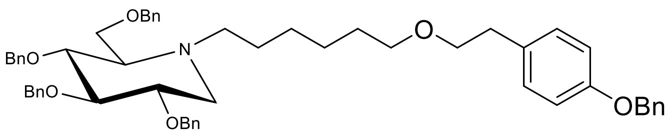

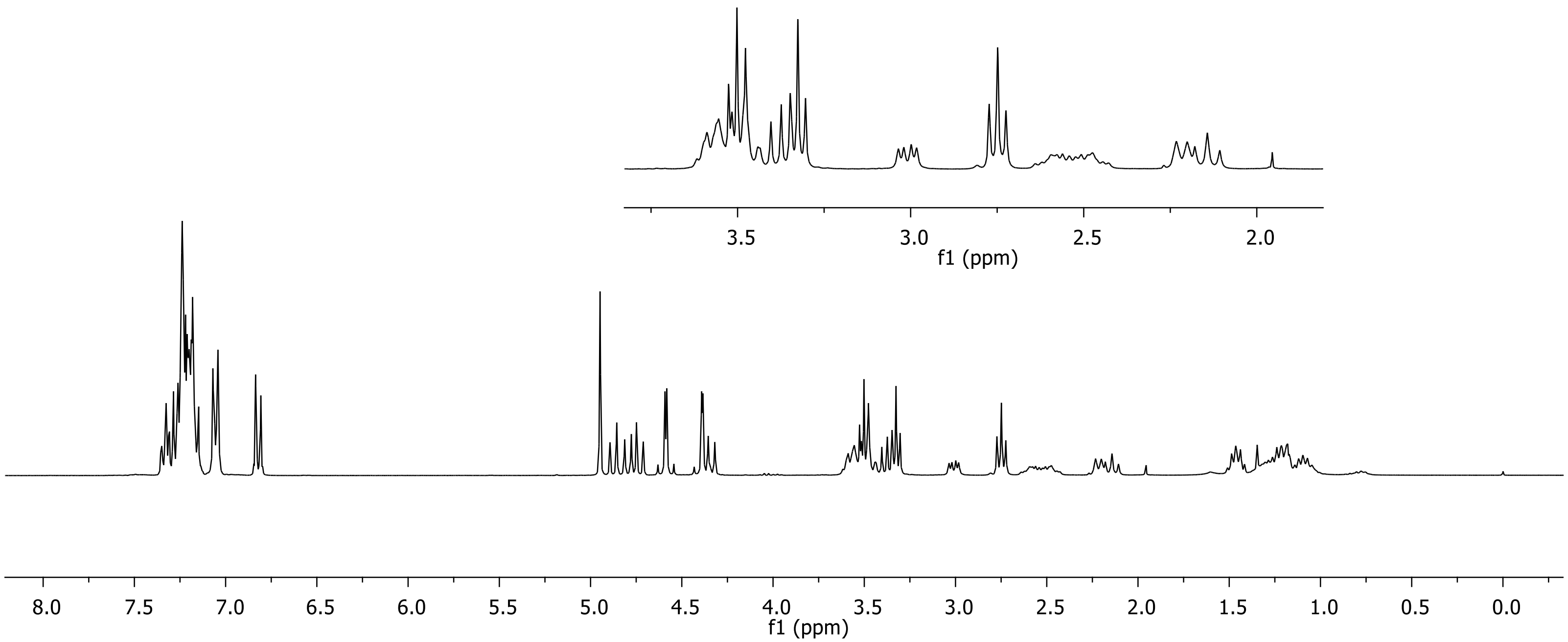

^{1}H-NMR (300 MHz, $CDCl_3$) of **9b**

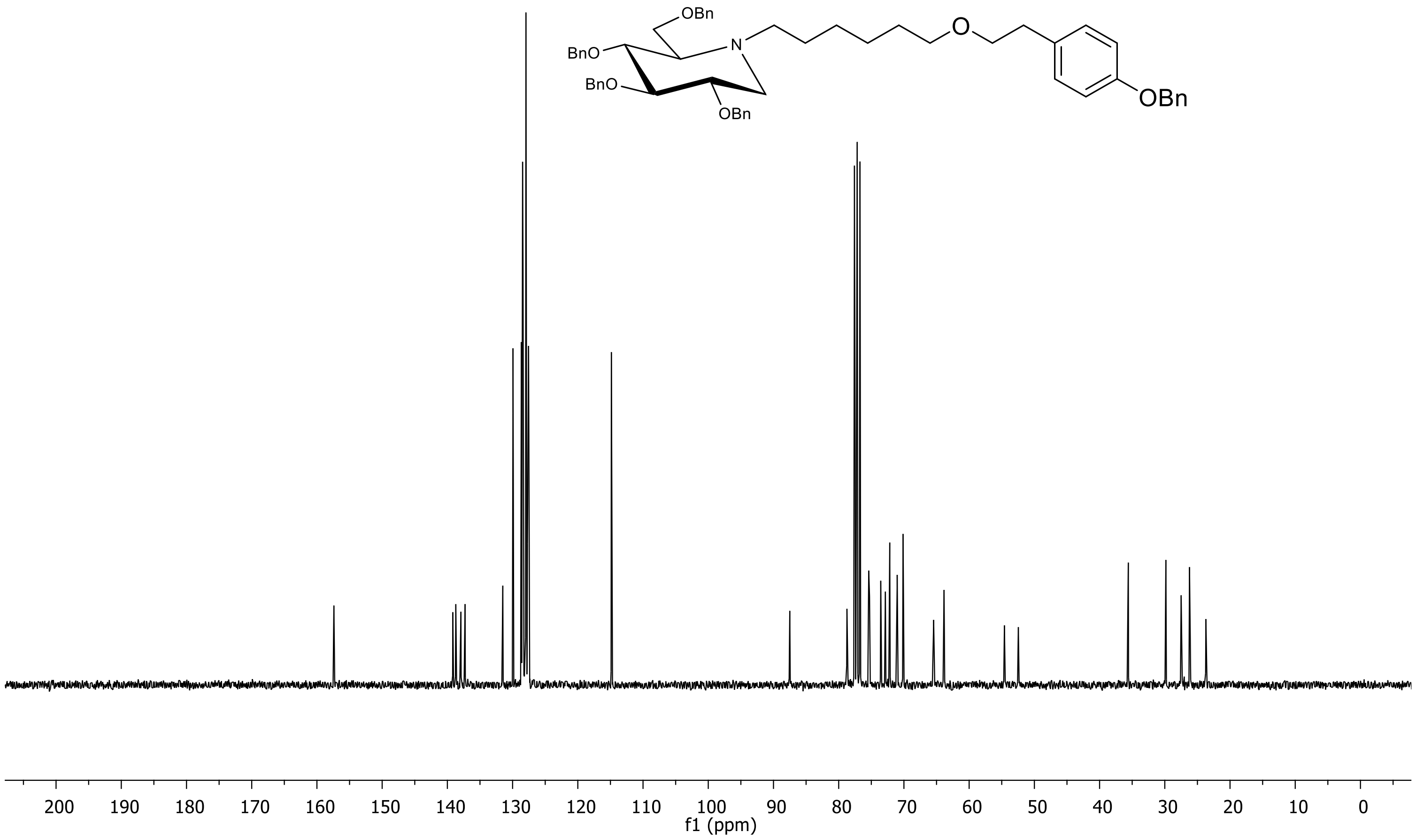

^{13}C-NMR (75.5 MHz, $CDCl_3$) of **9b**

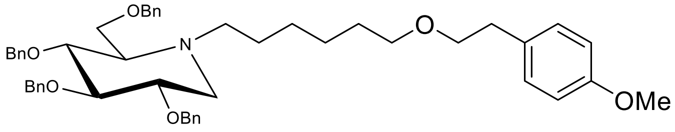

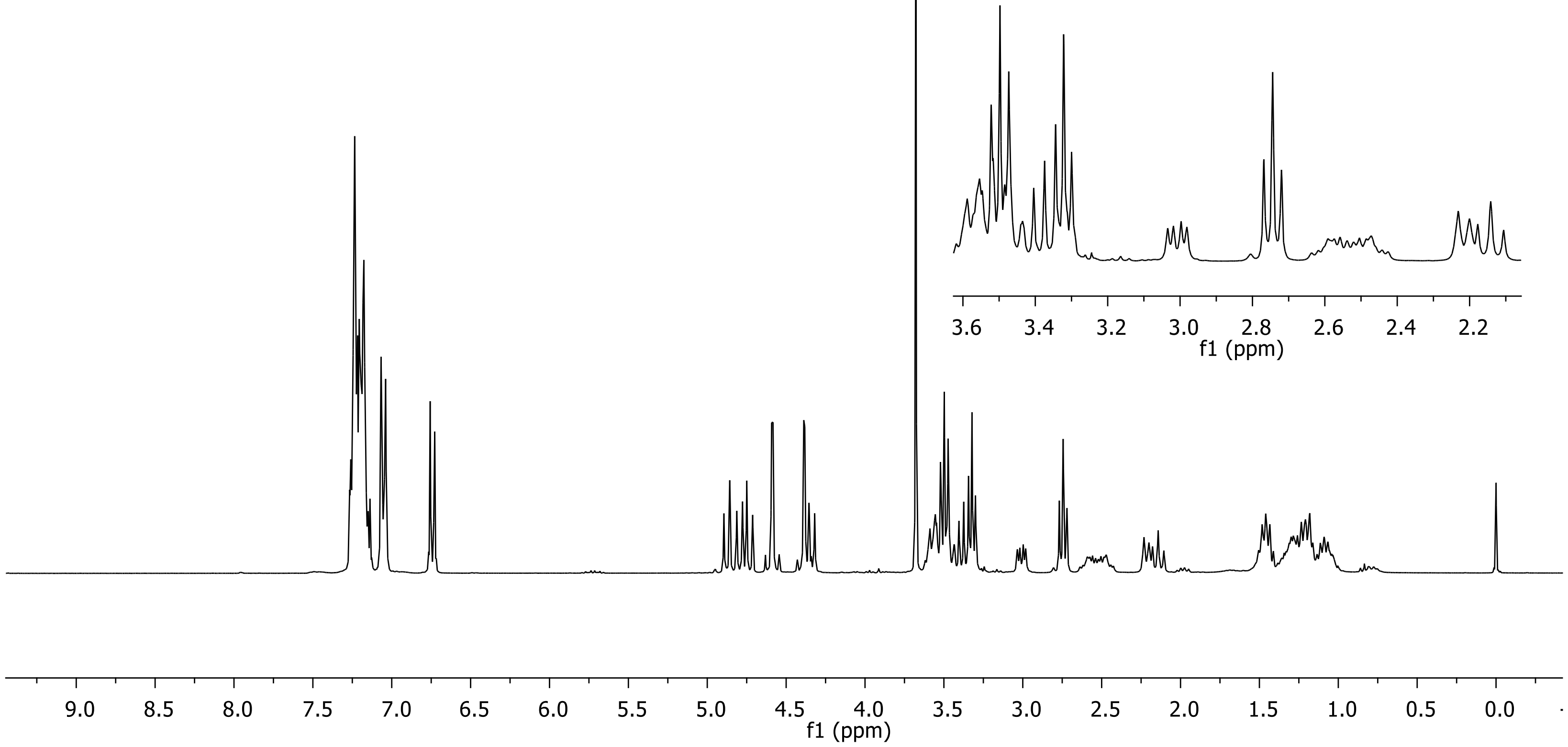

^{1}H-NMR (300 MHz, $CDCl_3$) of **9c**

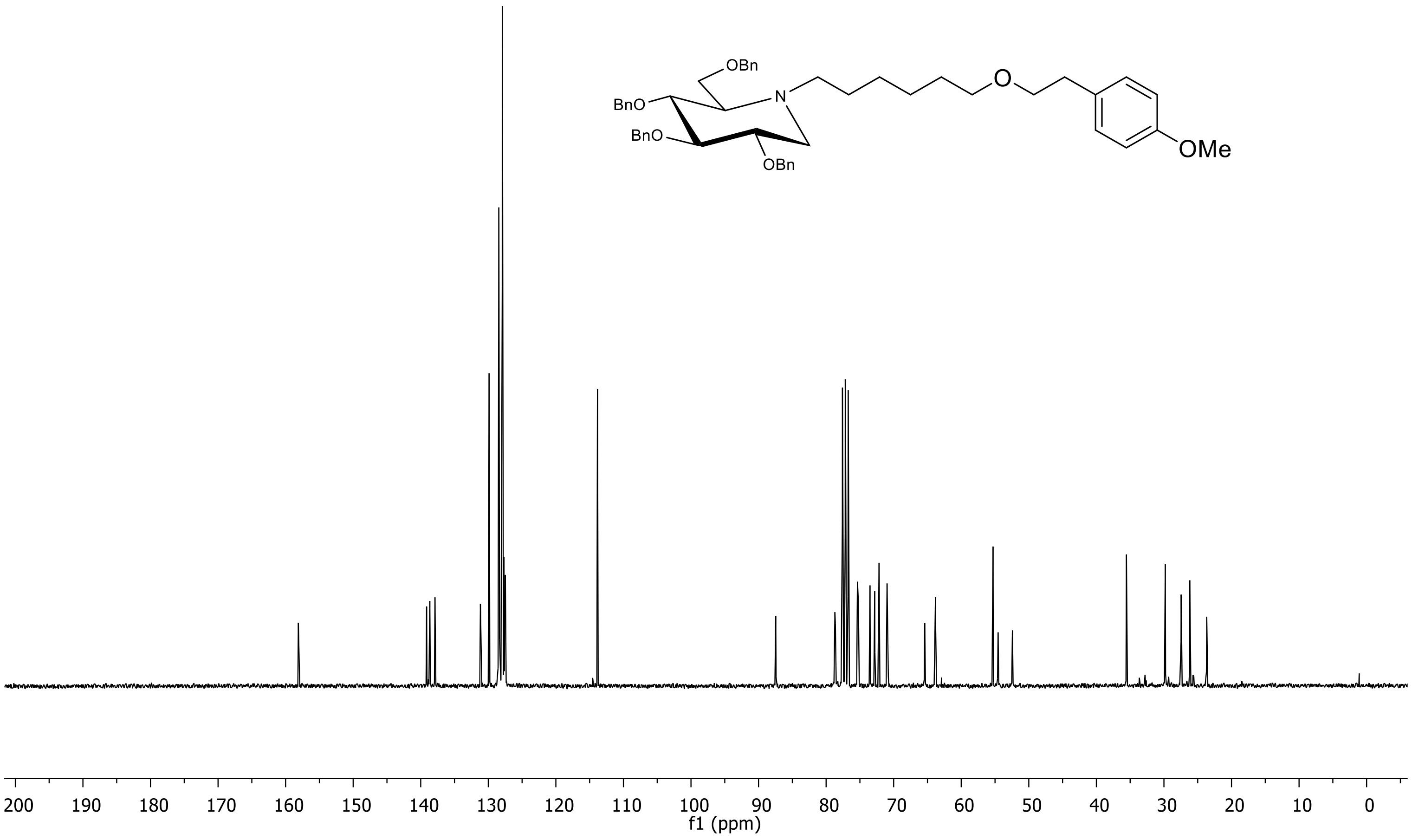

^{13}C-NMR (75.5 MHz, $CDCl_3$) of **9c**

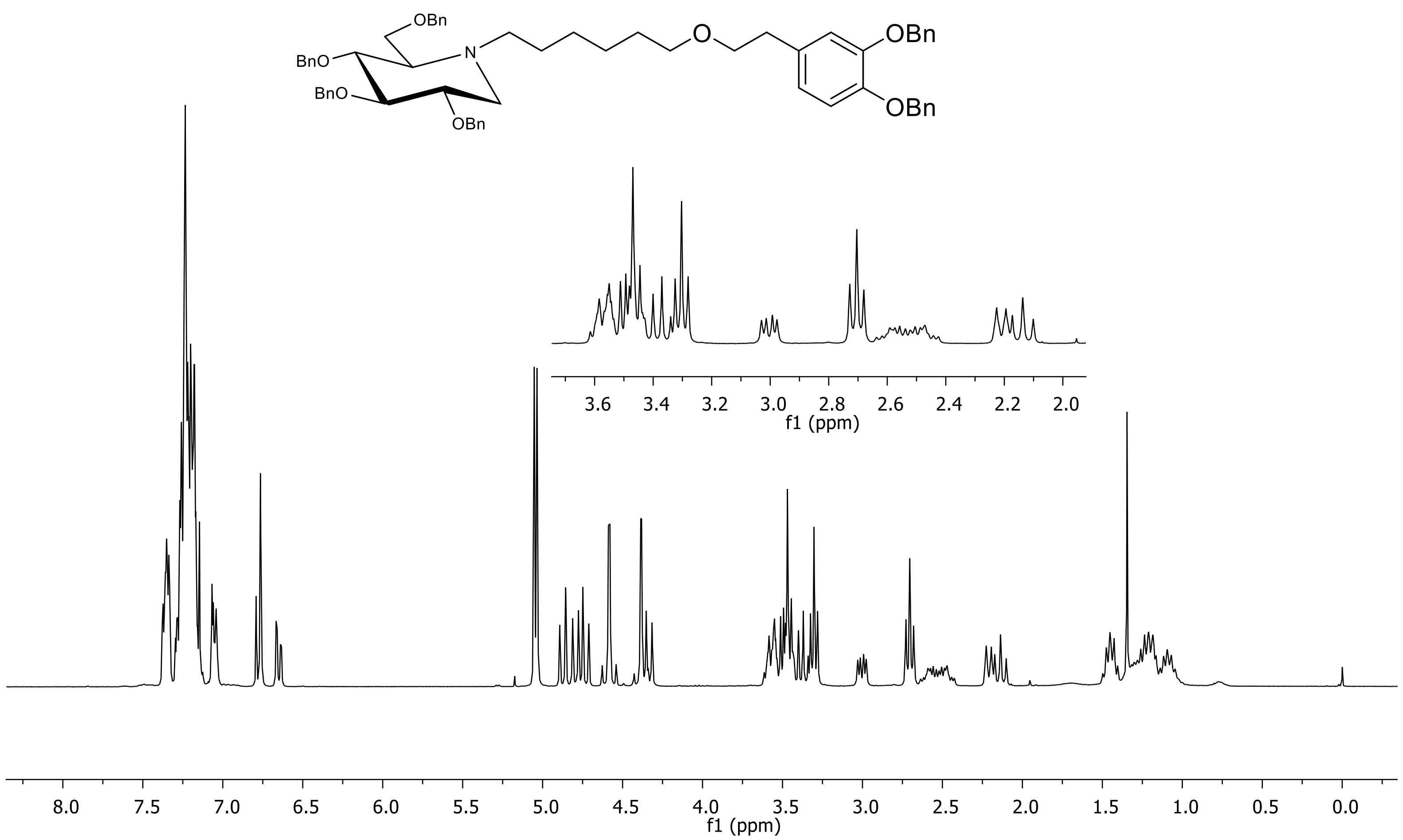

^{1}H-NMR (300 MHz, $CDCl_3$) of **9d**

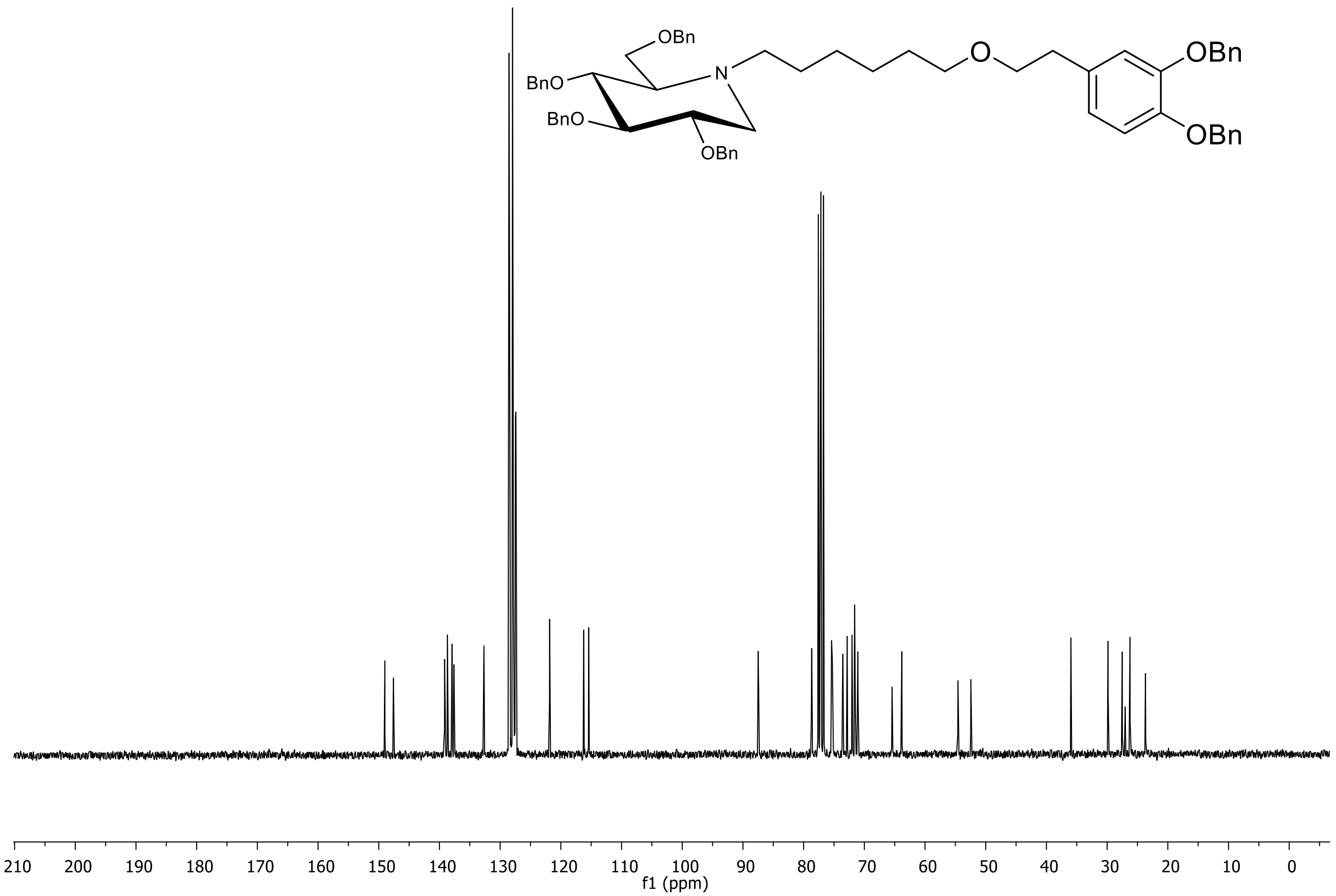

^{13}C-NMR (75.5 MHz, $CDCl_3$) of **9d**

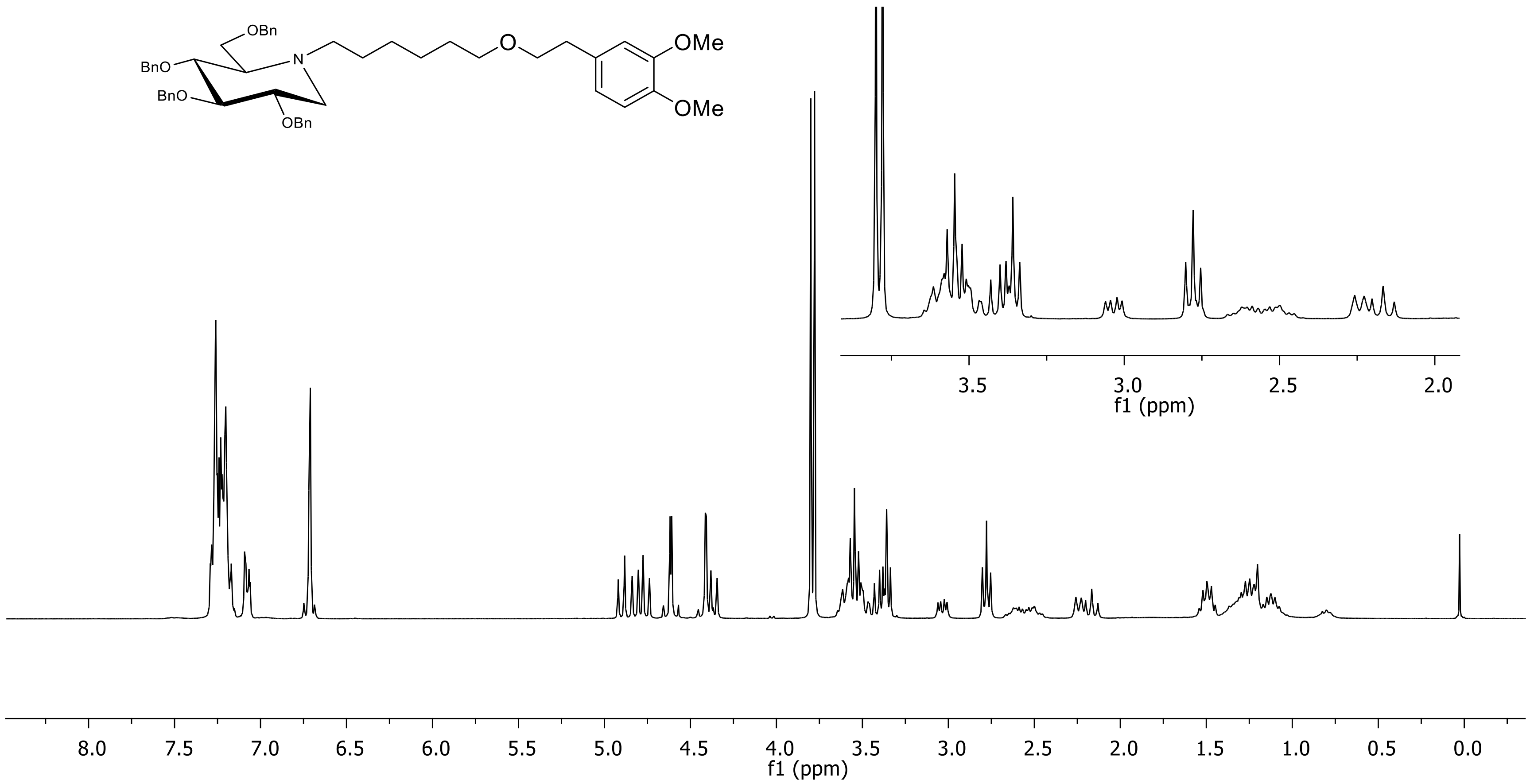

^{1}H-NMR (300 MHz, $CDCl_3$) of **9e**

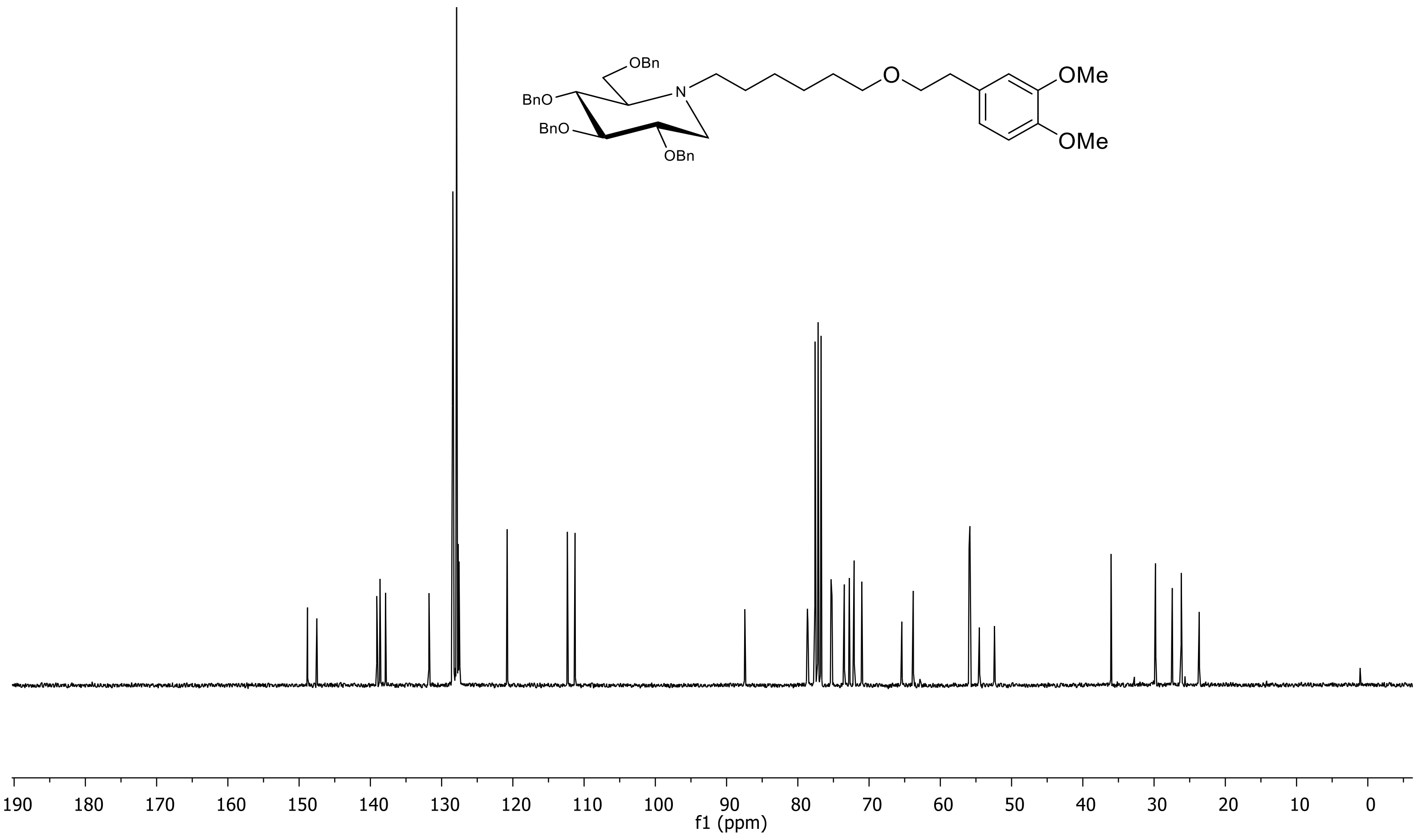

^{13}C-NMR (75.5 MHz, $CDCl_3$) of **9e**

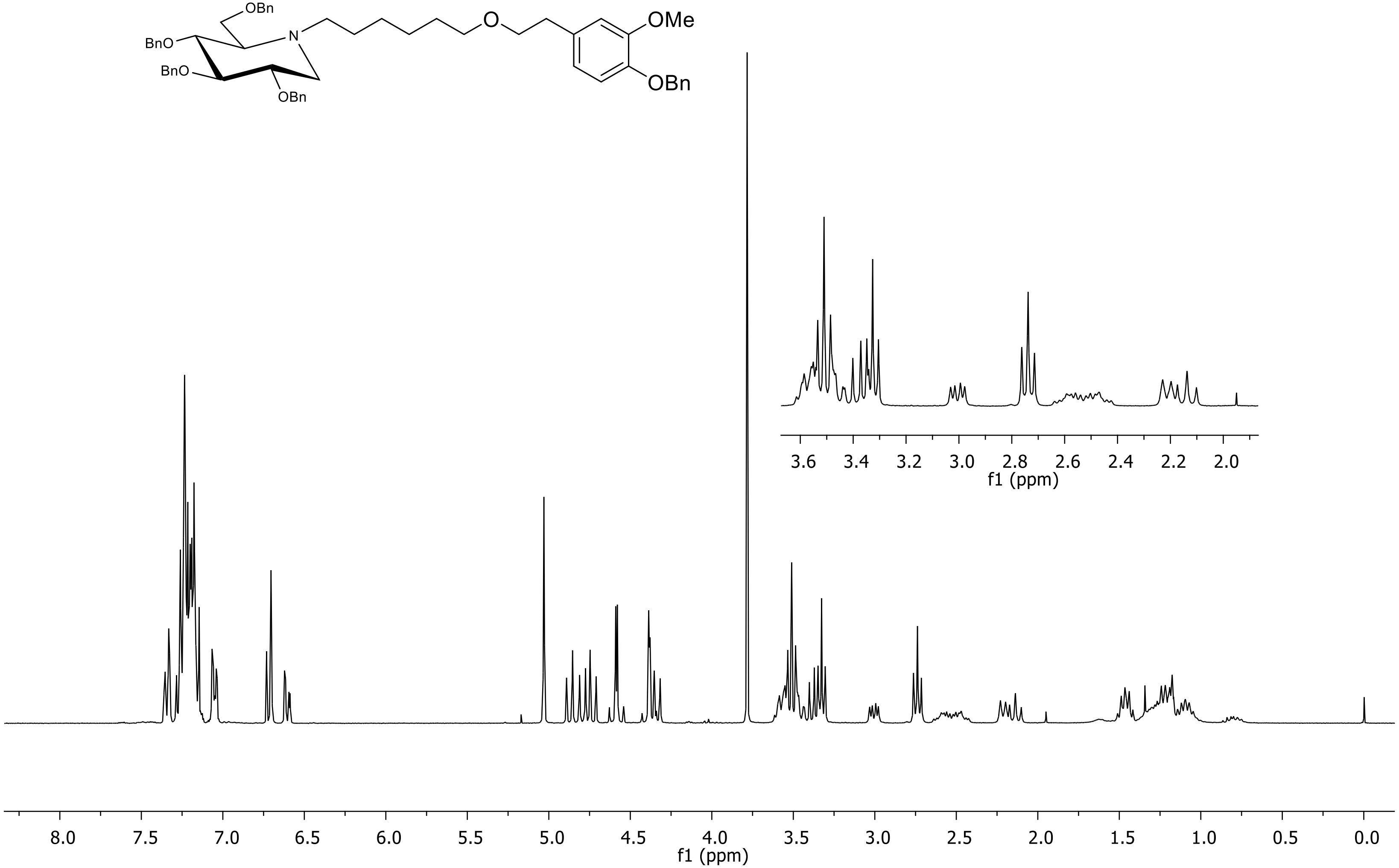

^{1}H-NMR (300 MHz, $CDCl_3$) of **9f**

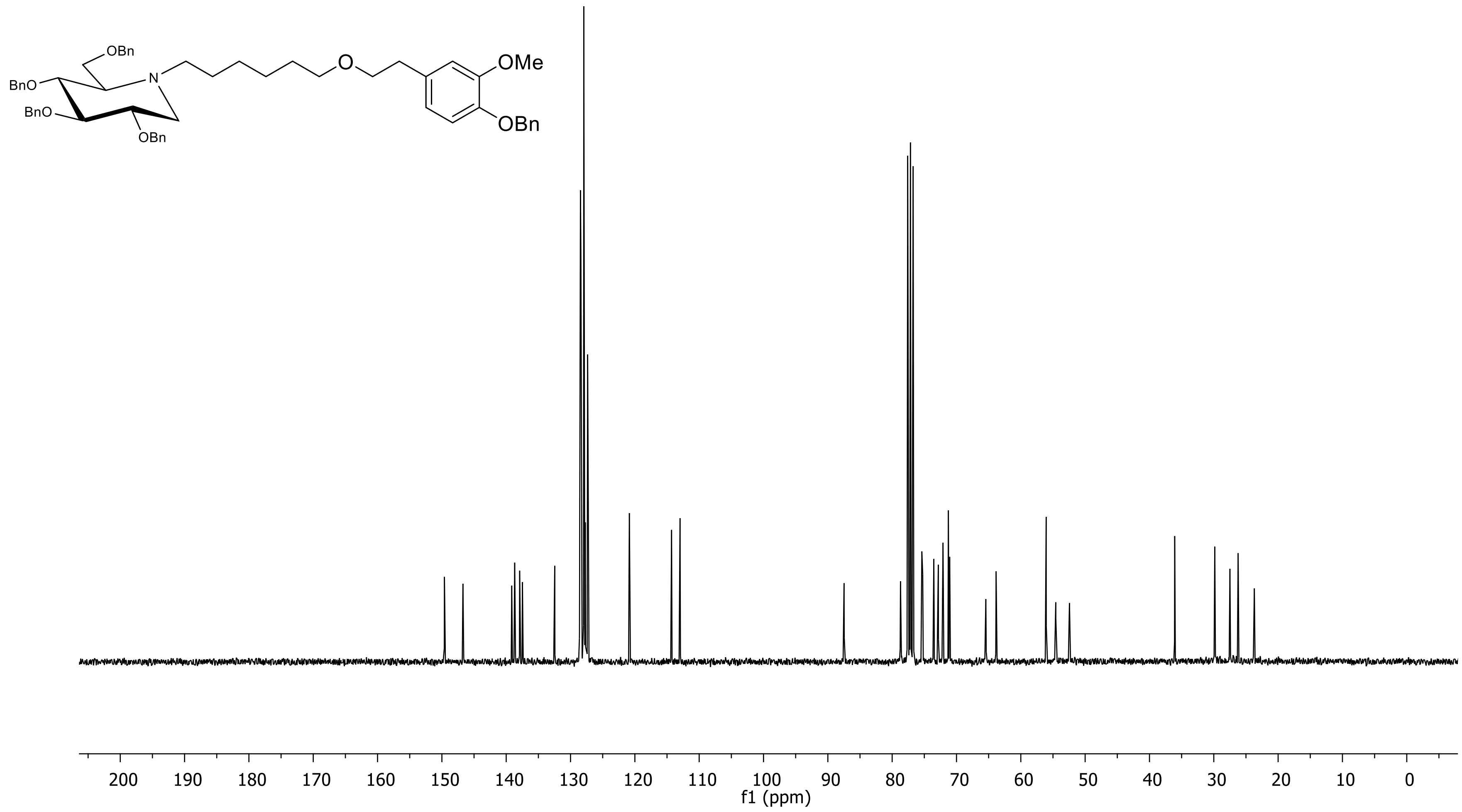

^{13}C-NMR (75.5 MHz, $CDCl_3$) of **9f**

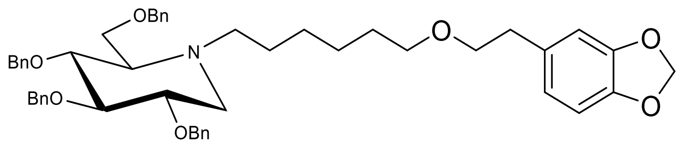

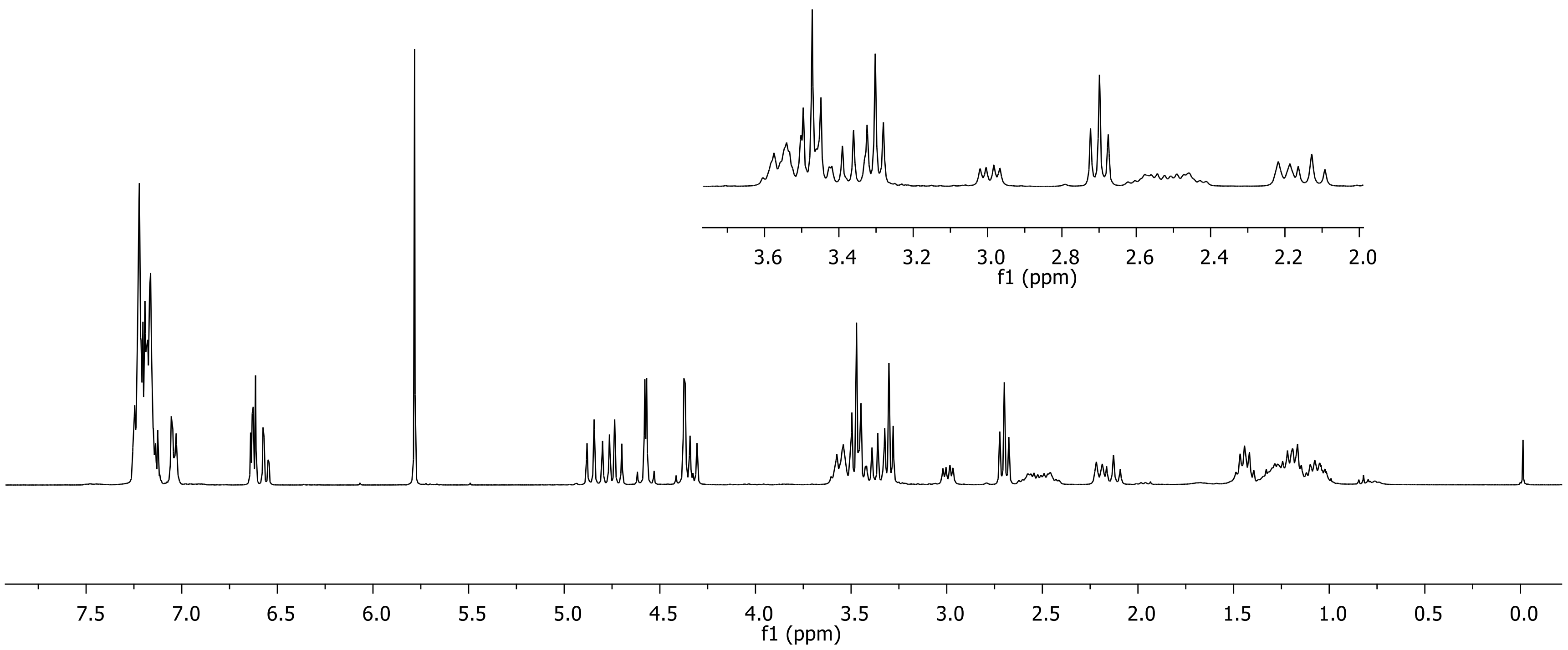

^{1}H-NMR (300 MHz, $CDCl_3$) of **9g**

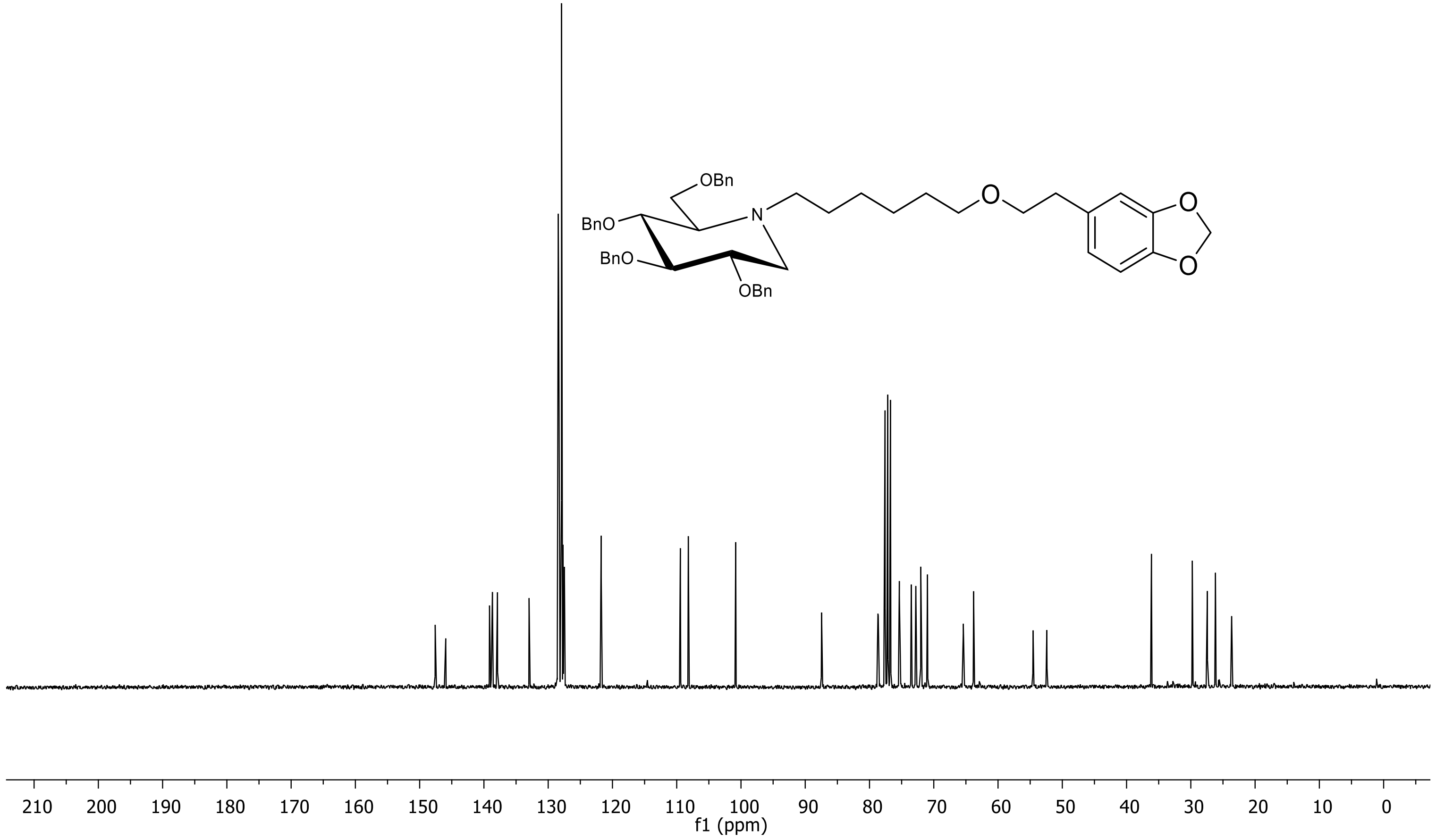

^{13}C-NMR (75.5 MHz, $CDCl_3$) of **9g**

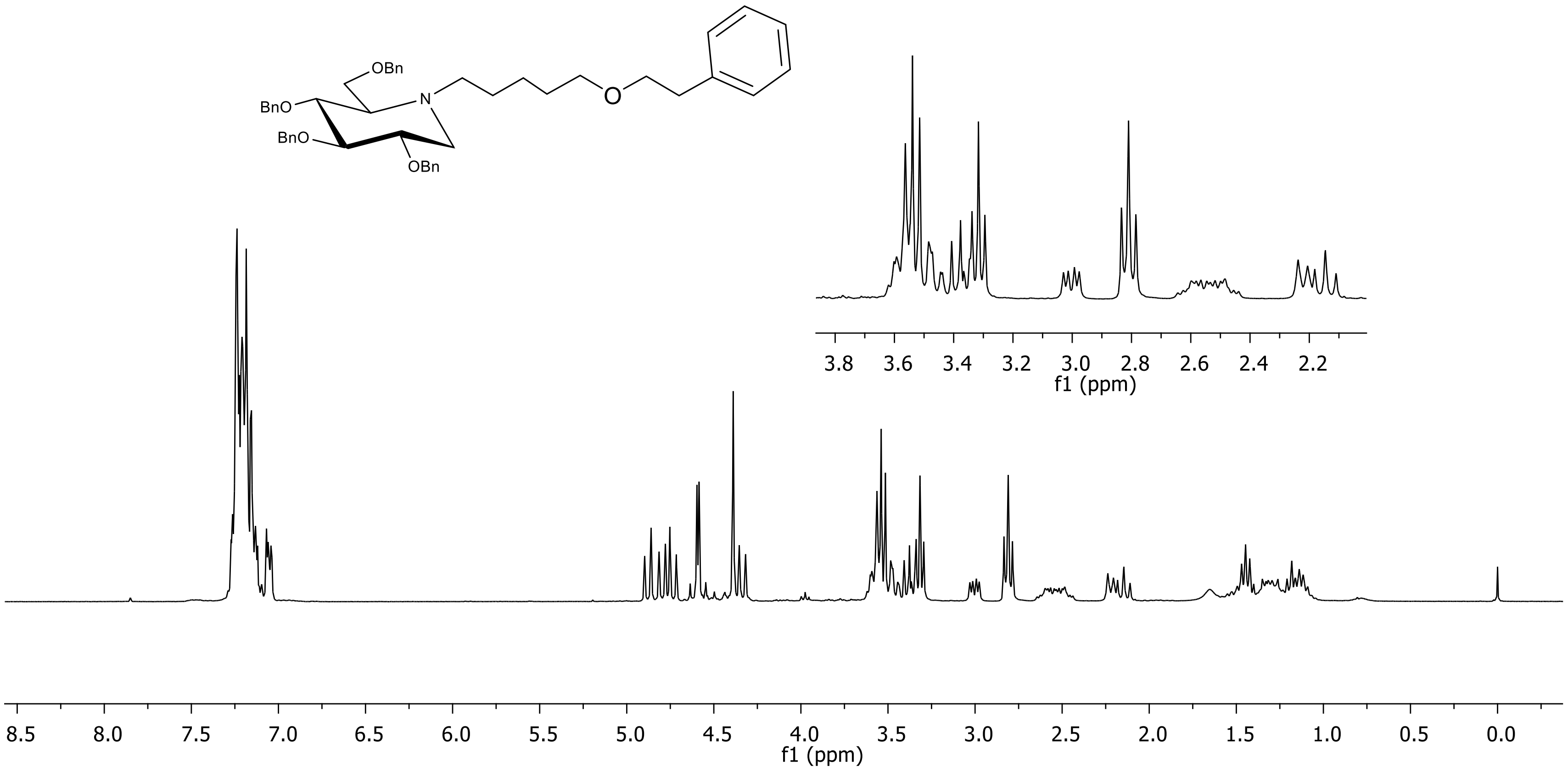

^{1}H-NMR (300 MHz, $CDCl_3$) of **10a**

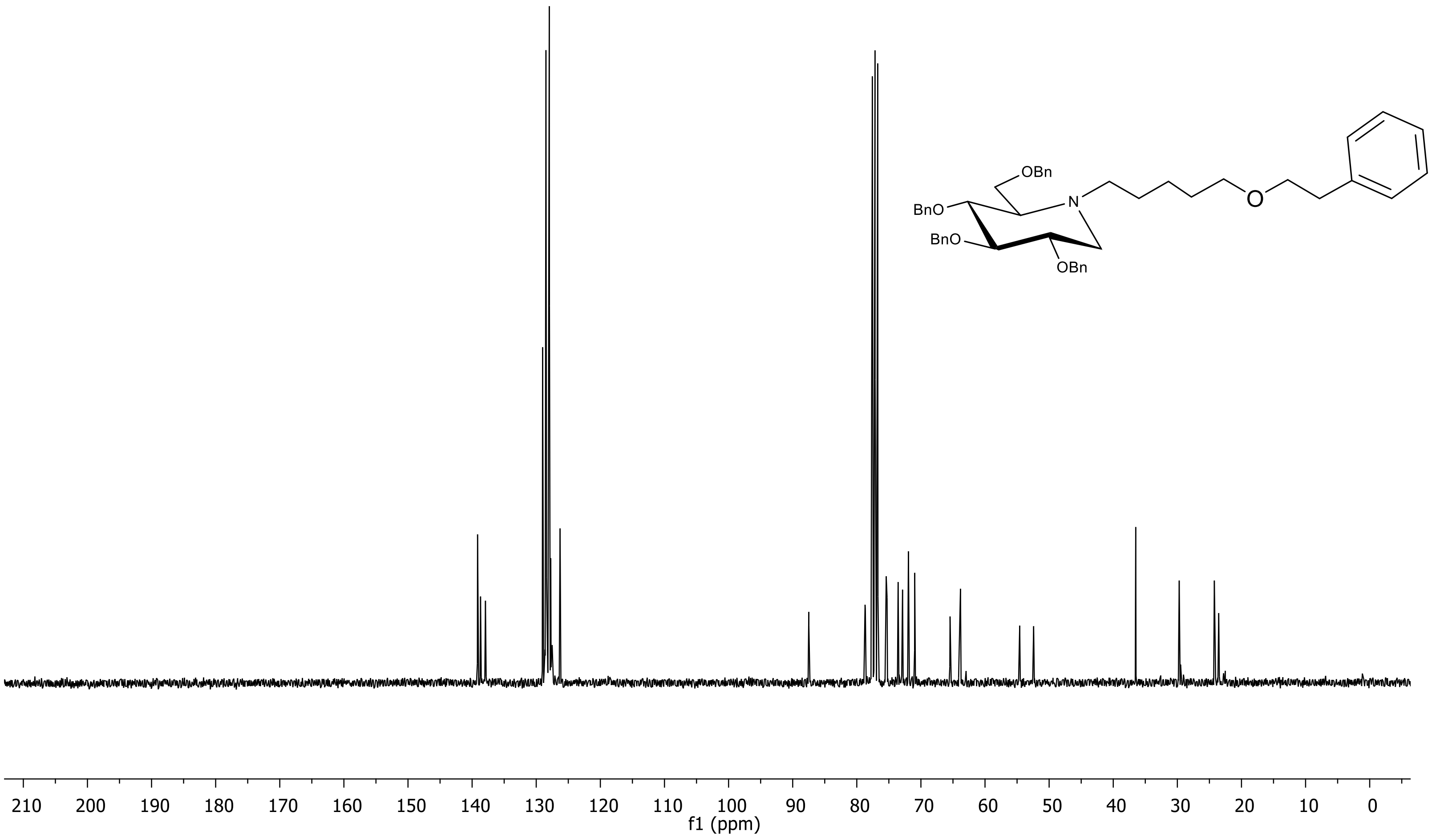

^{13}C-NMR (75.5 MHz, $CDCl_3$) of **10a**

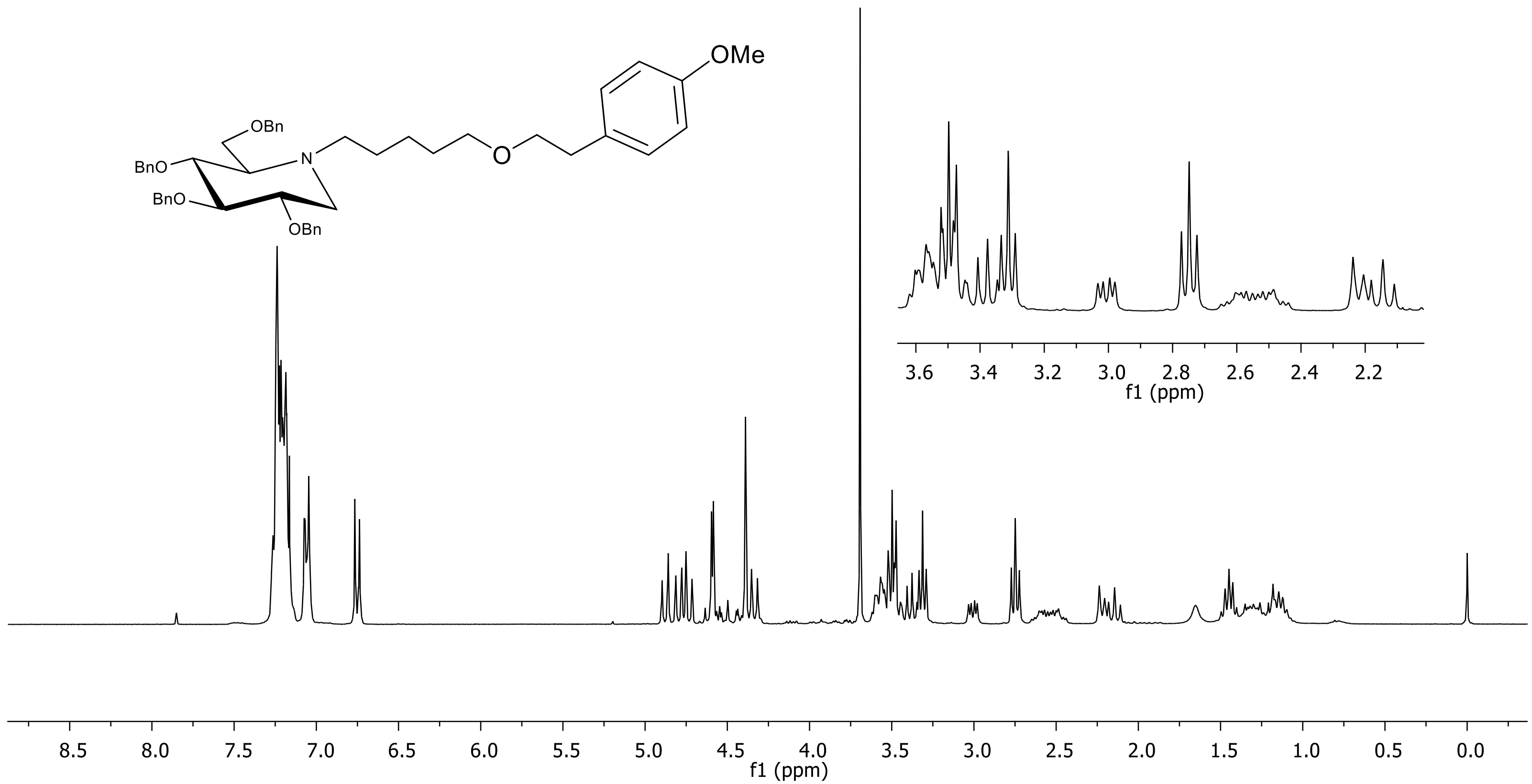

^{1}H-NMR (300 MHz, $CDCl_3$) of **10c**

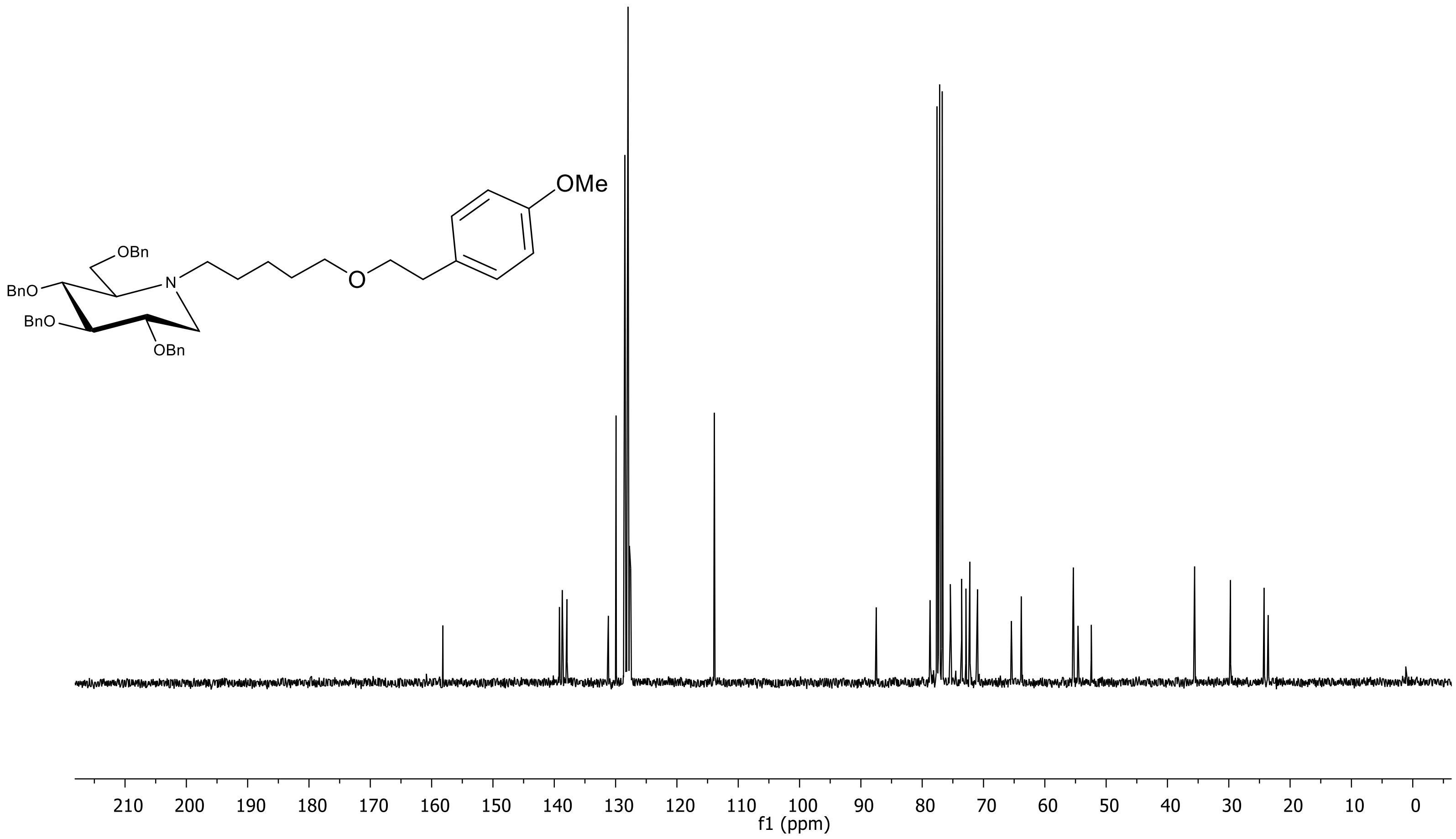

^{13}C-NMR (75.5 MHz, CDCl$_3$) of **10c**

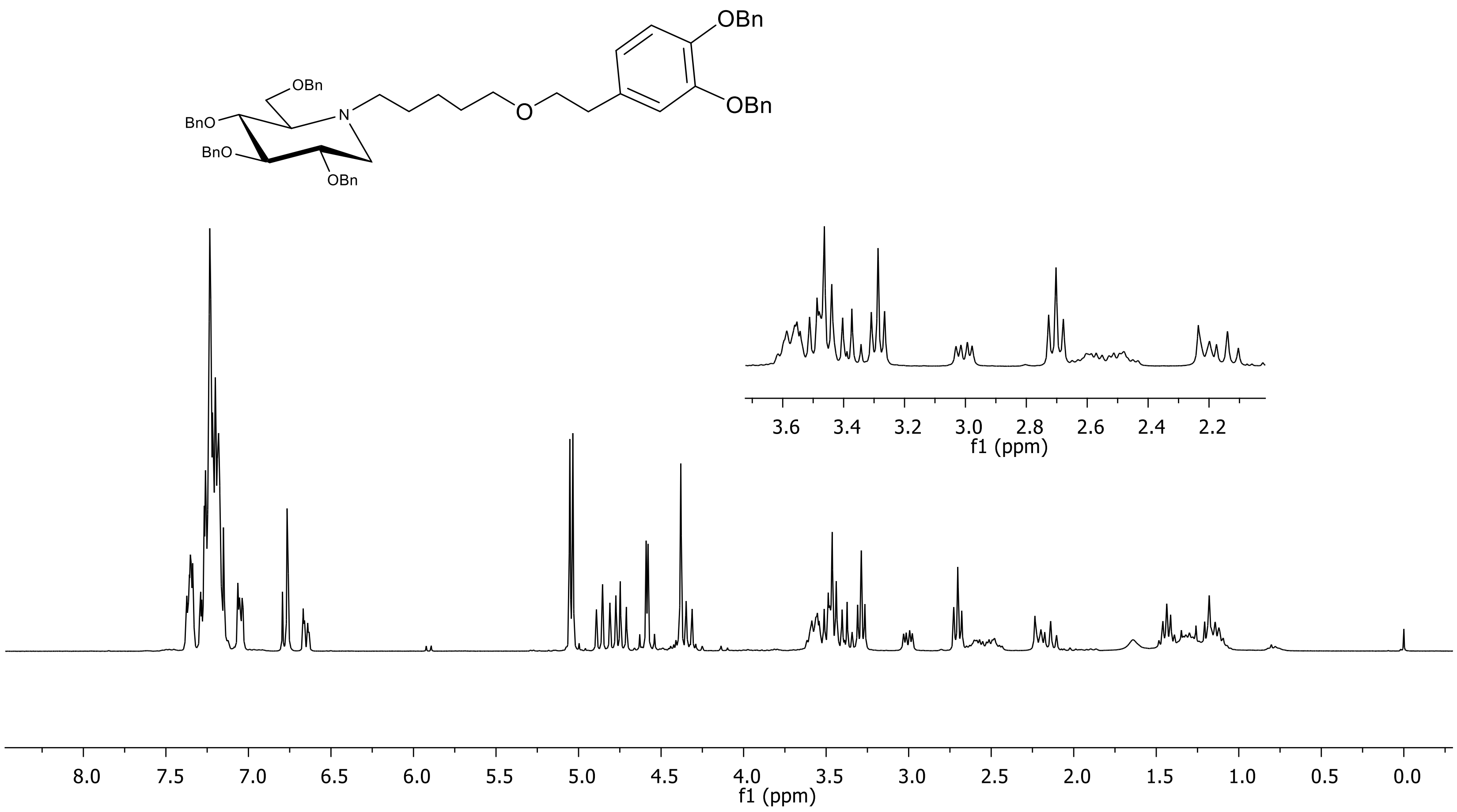

^{1}H-NMR (300 MHz, $CDCl_3$) of **10d**

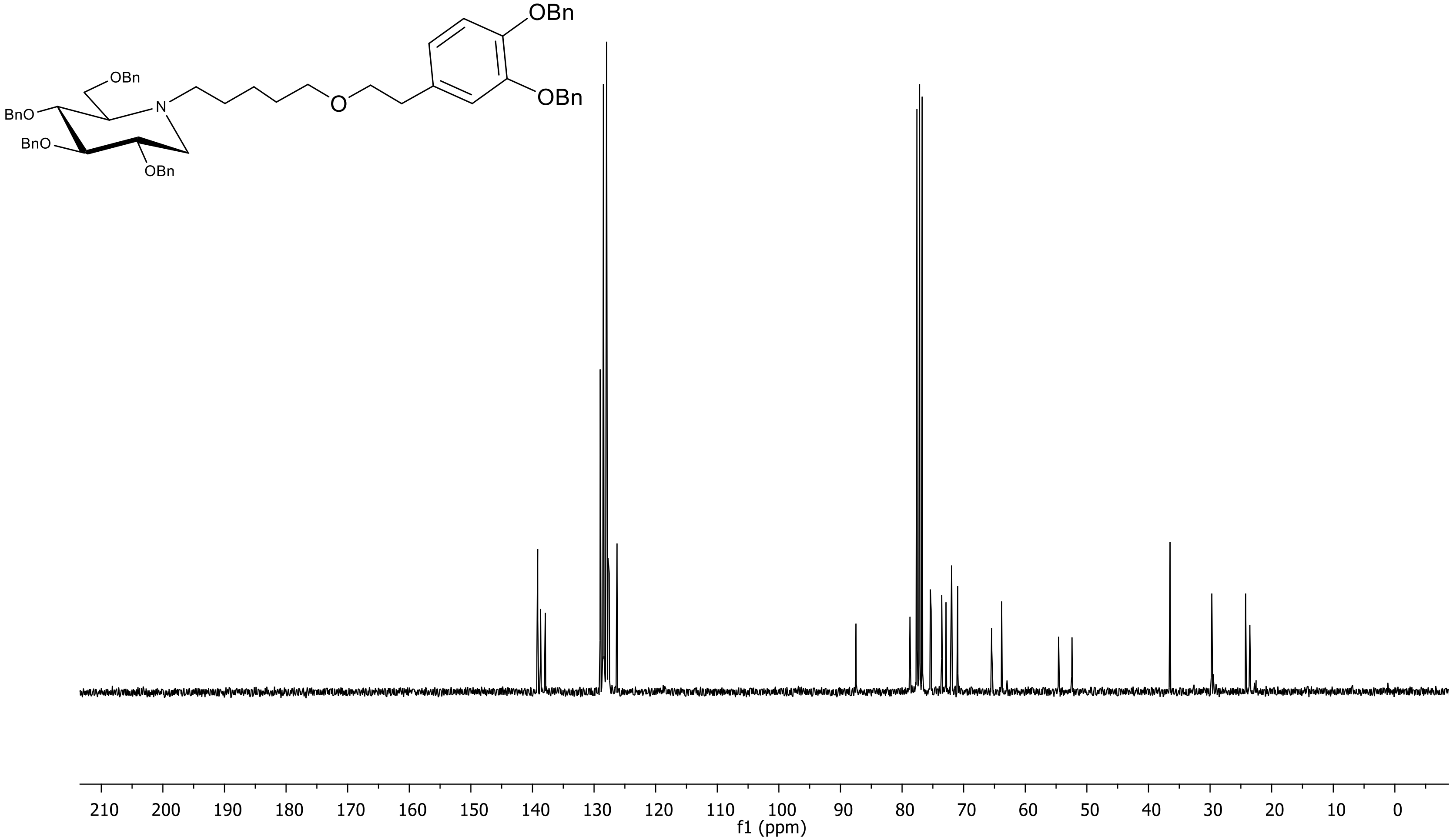

^{13}C-NMR (75.5 MHz, $CDCl_3$) of **10d**

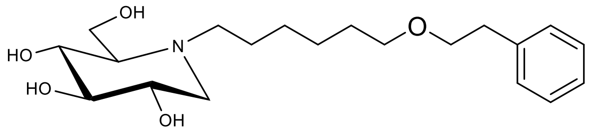

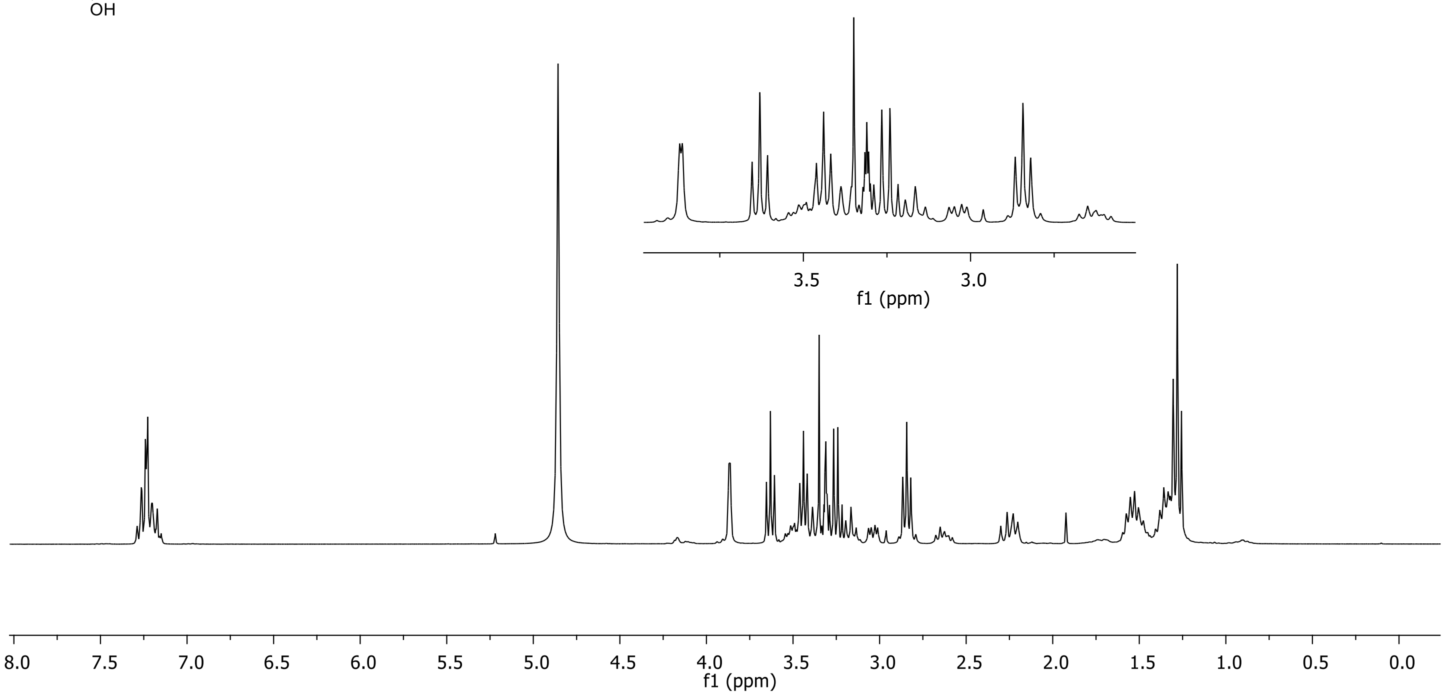

^{1}H-NMR (300 MHz, CD_3OD) of **11a**

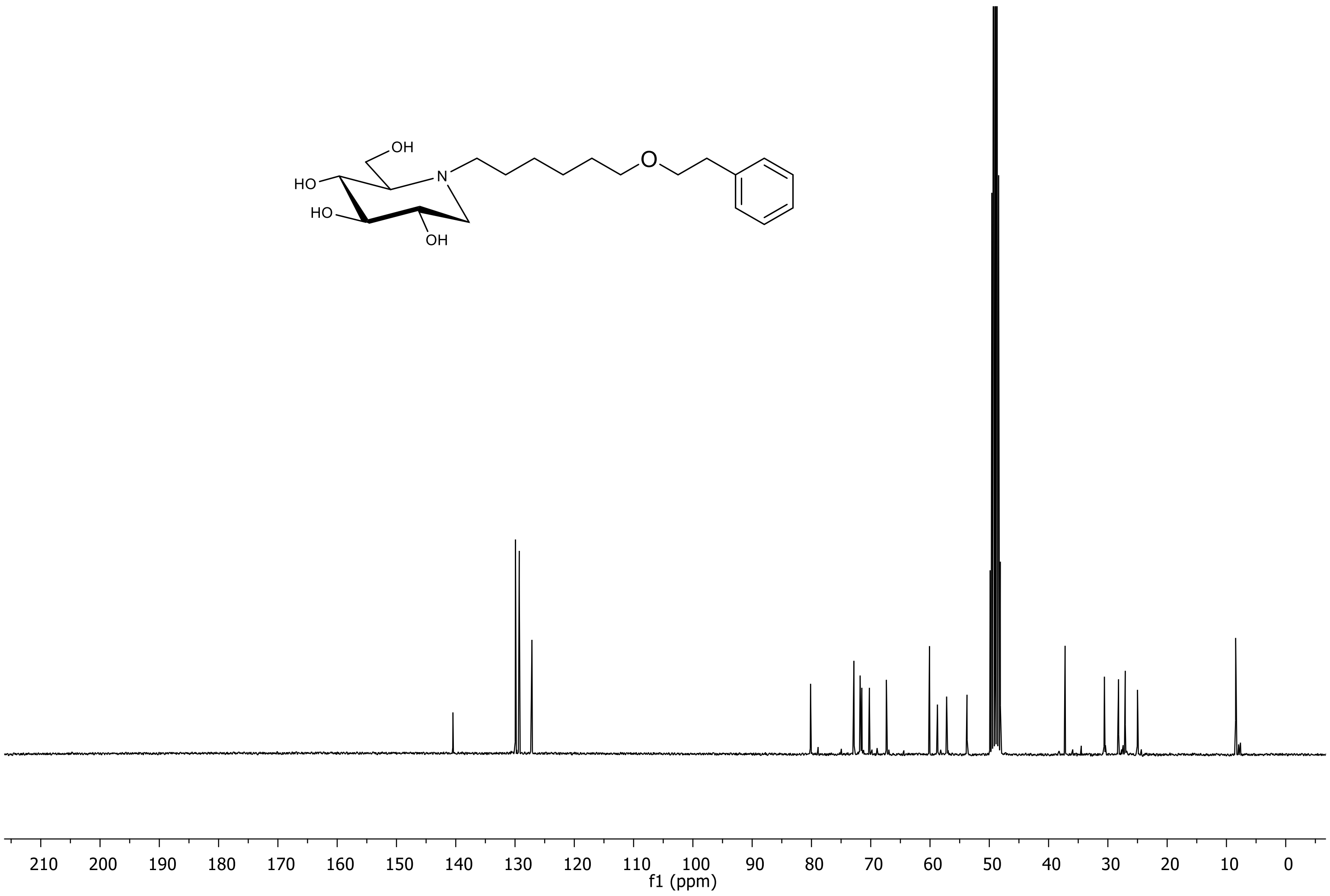

^{13}C-NMR (75.5 MHz, CD_3OD) of **11a**

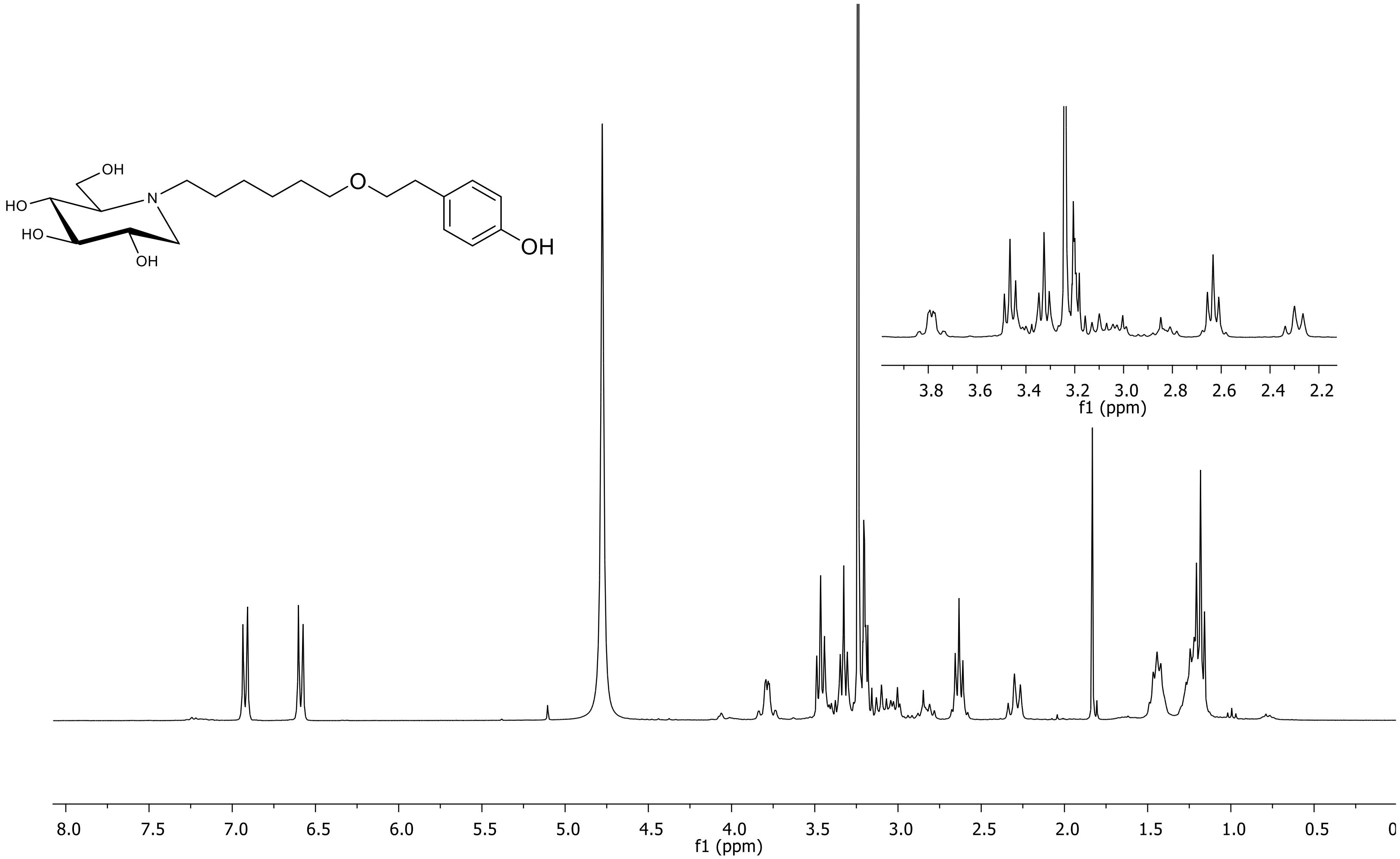

^{1}H-NMR (300 MHz, CD_3OD) of **11b**

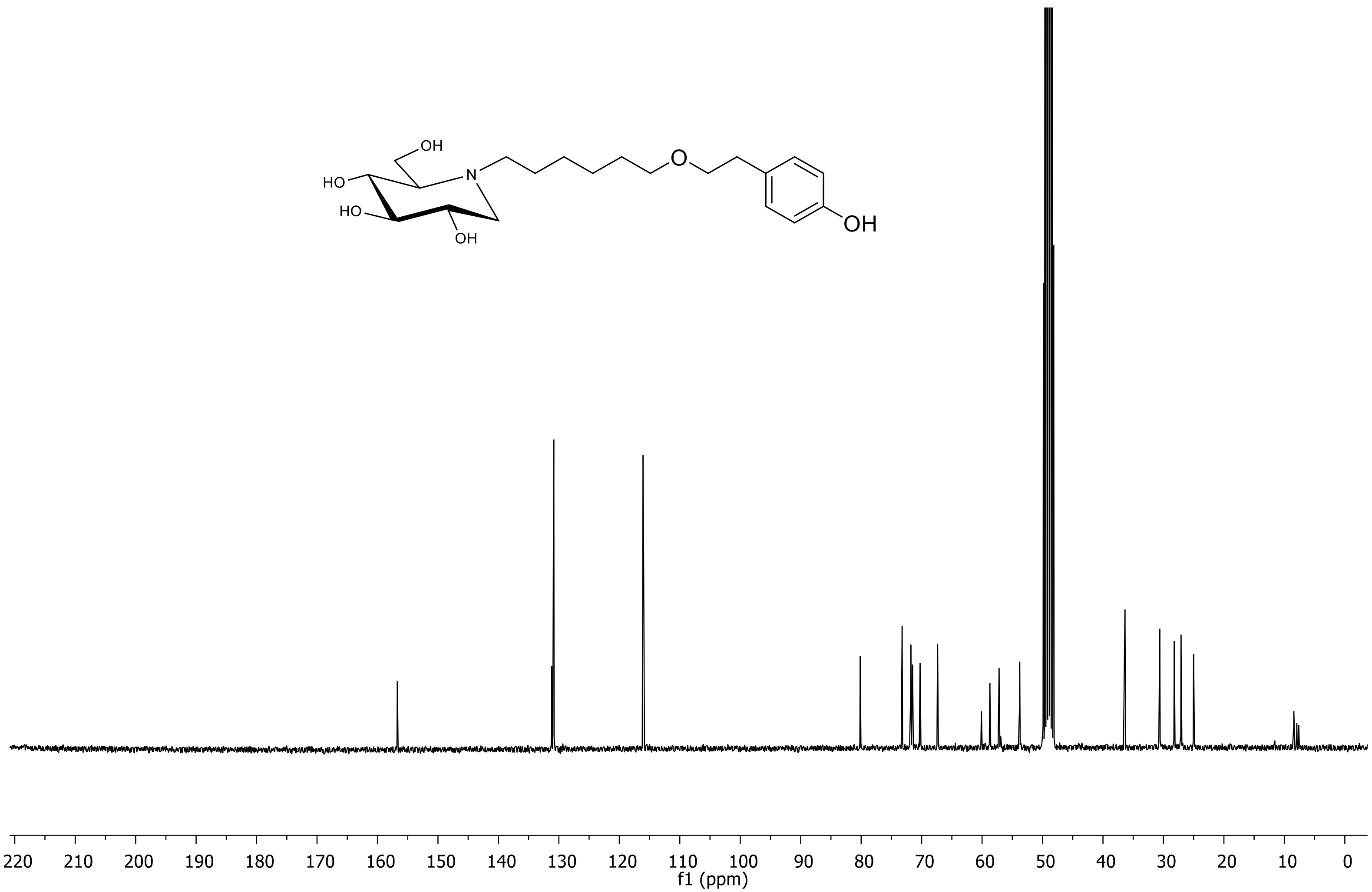

^{13}C-NMR (75.5 MHz, CD_3OD) of **11b**

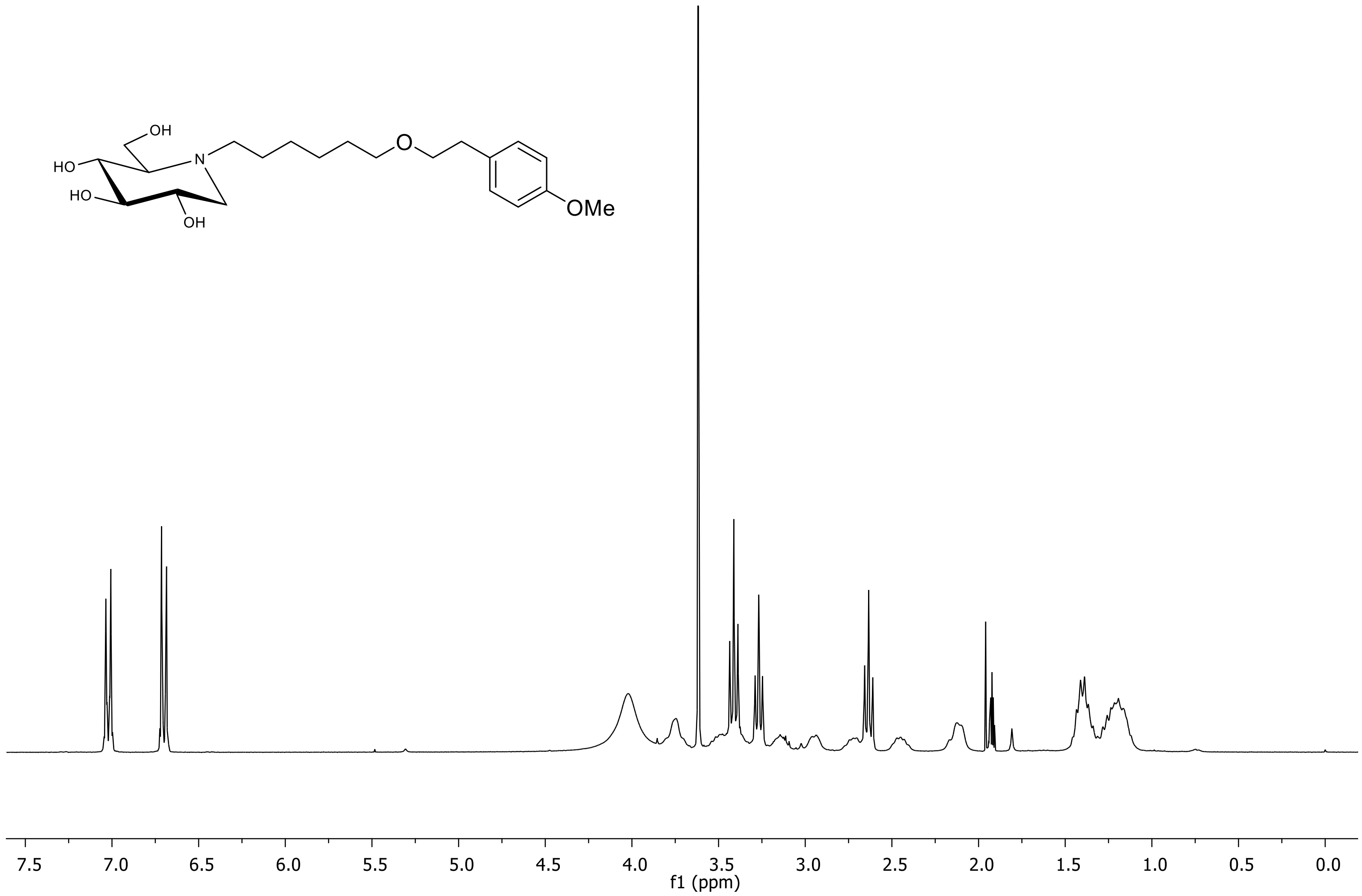

^{1}H-NMR (300 MHz, $(CD_3)_2CO$) of **11c**

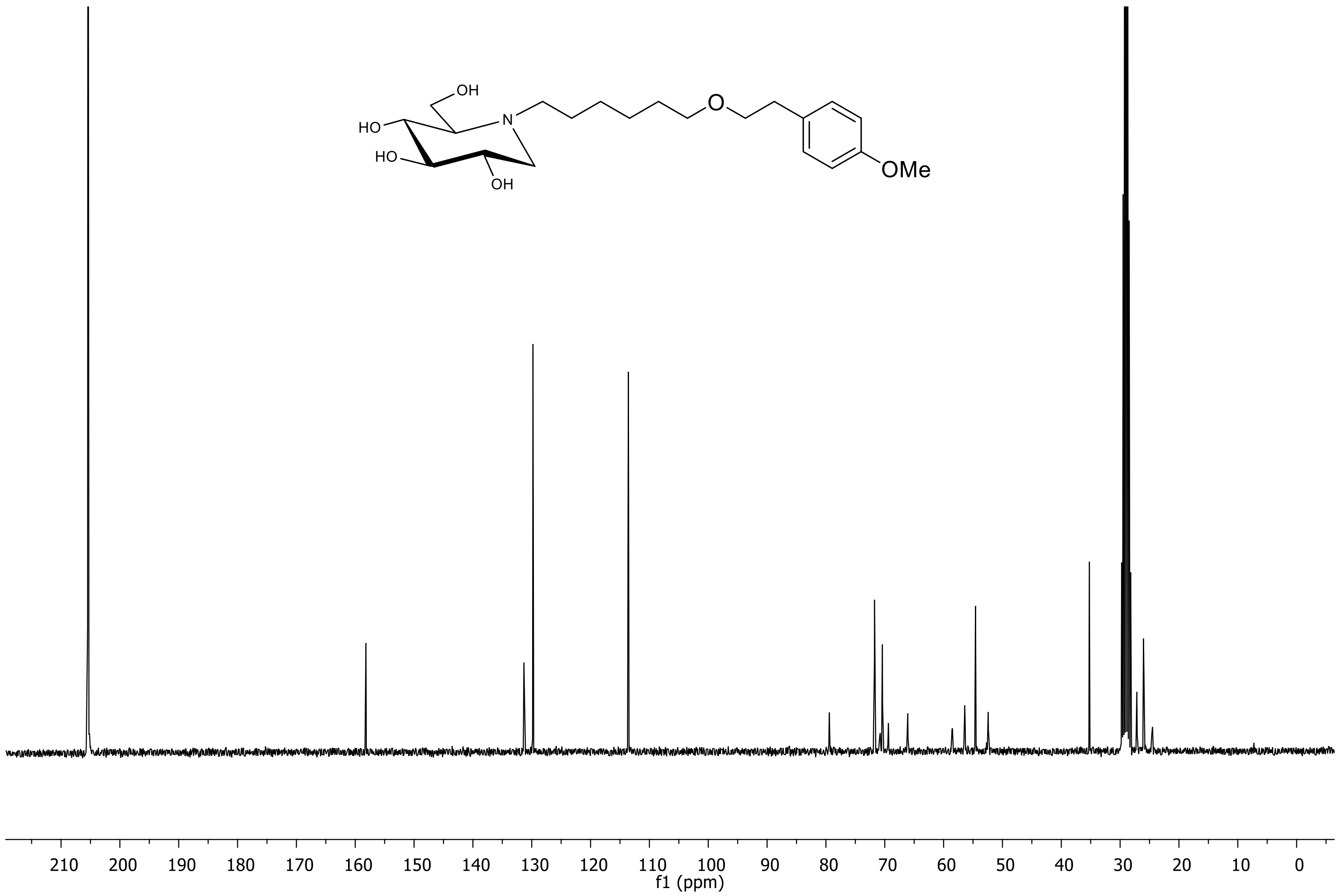

^{13}C-NMR (75.5 MHz, $(CD_3)_2CO$) of **11c**

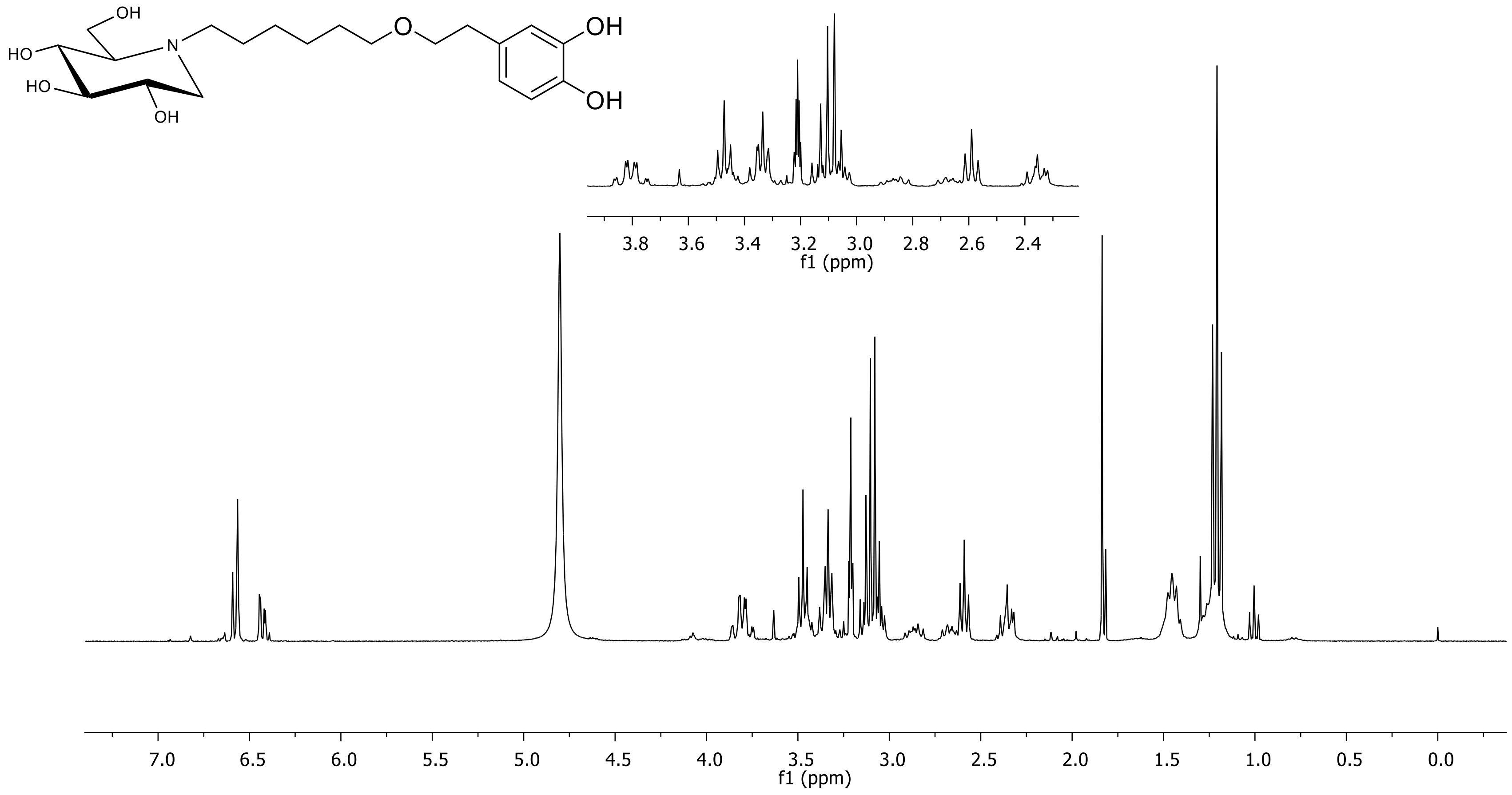

^{1}H-NMR (300 MHz, CD_3OD) of **11d**

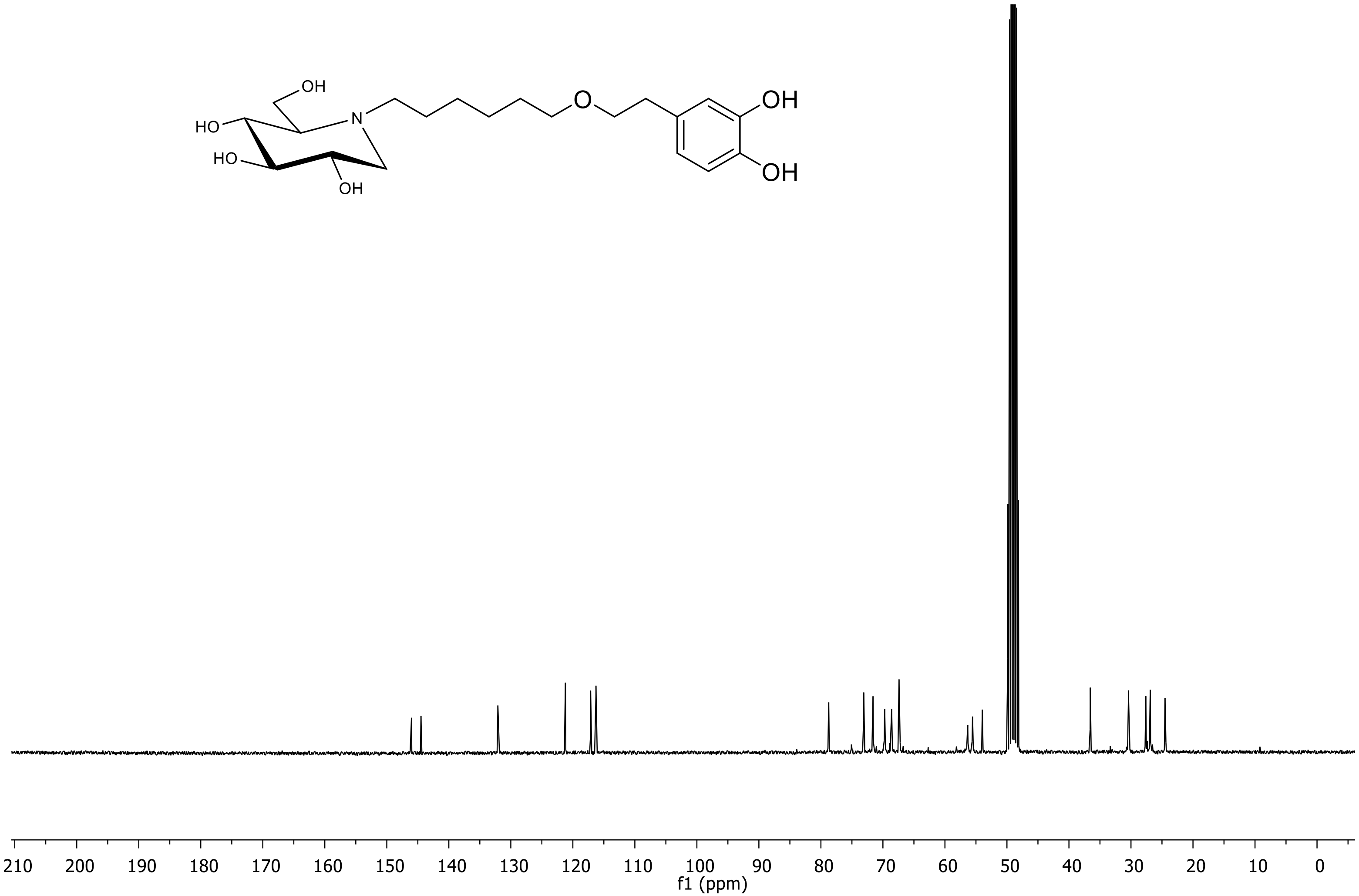

^{13}C-NMR (75.5 MHz, CD_3OD) of **11d**

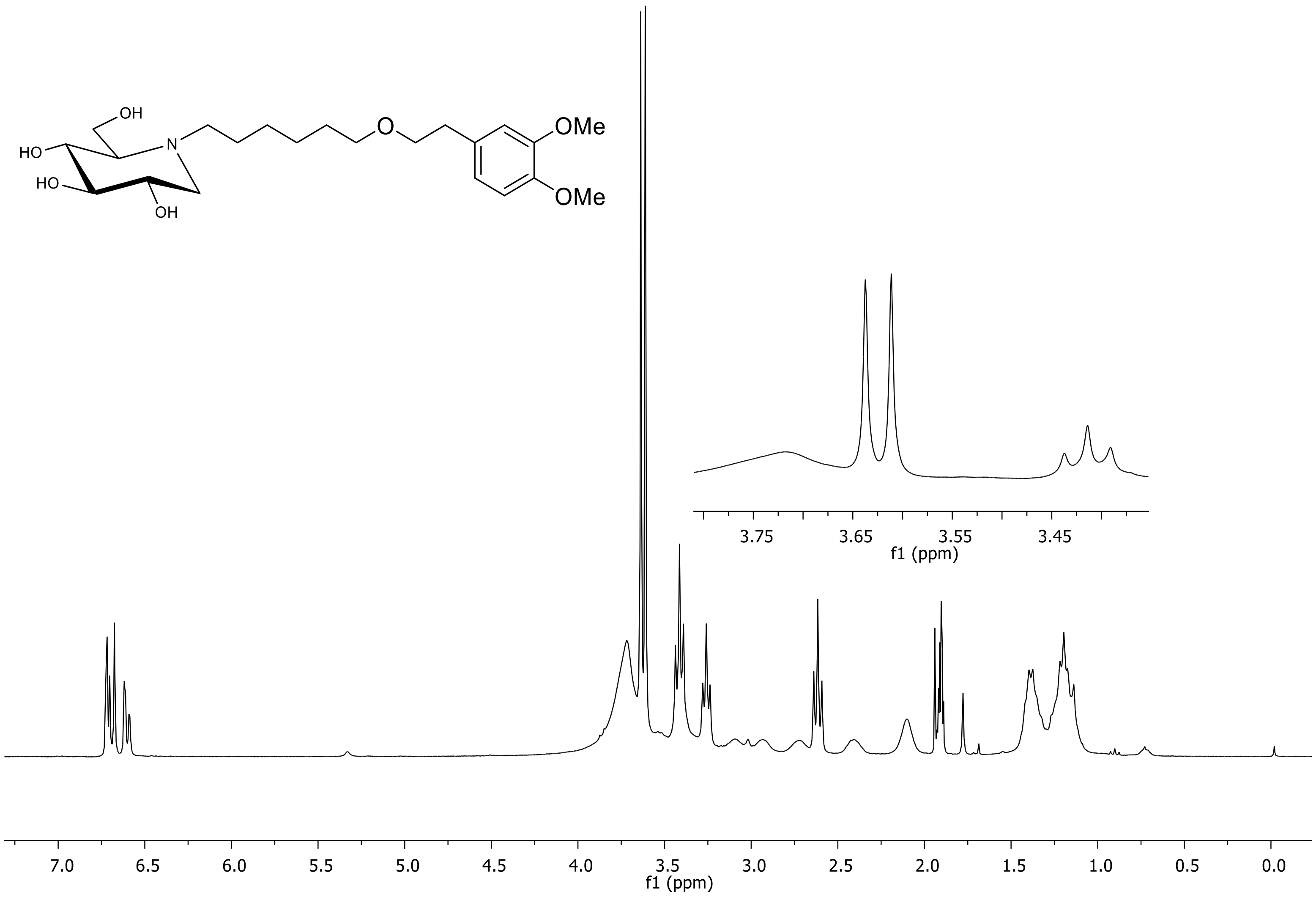

^{1}H-NMR (300 MHz, $(CD_3)_2CO$) of **11e**

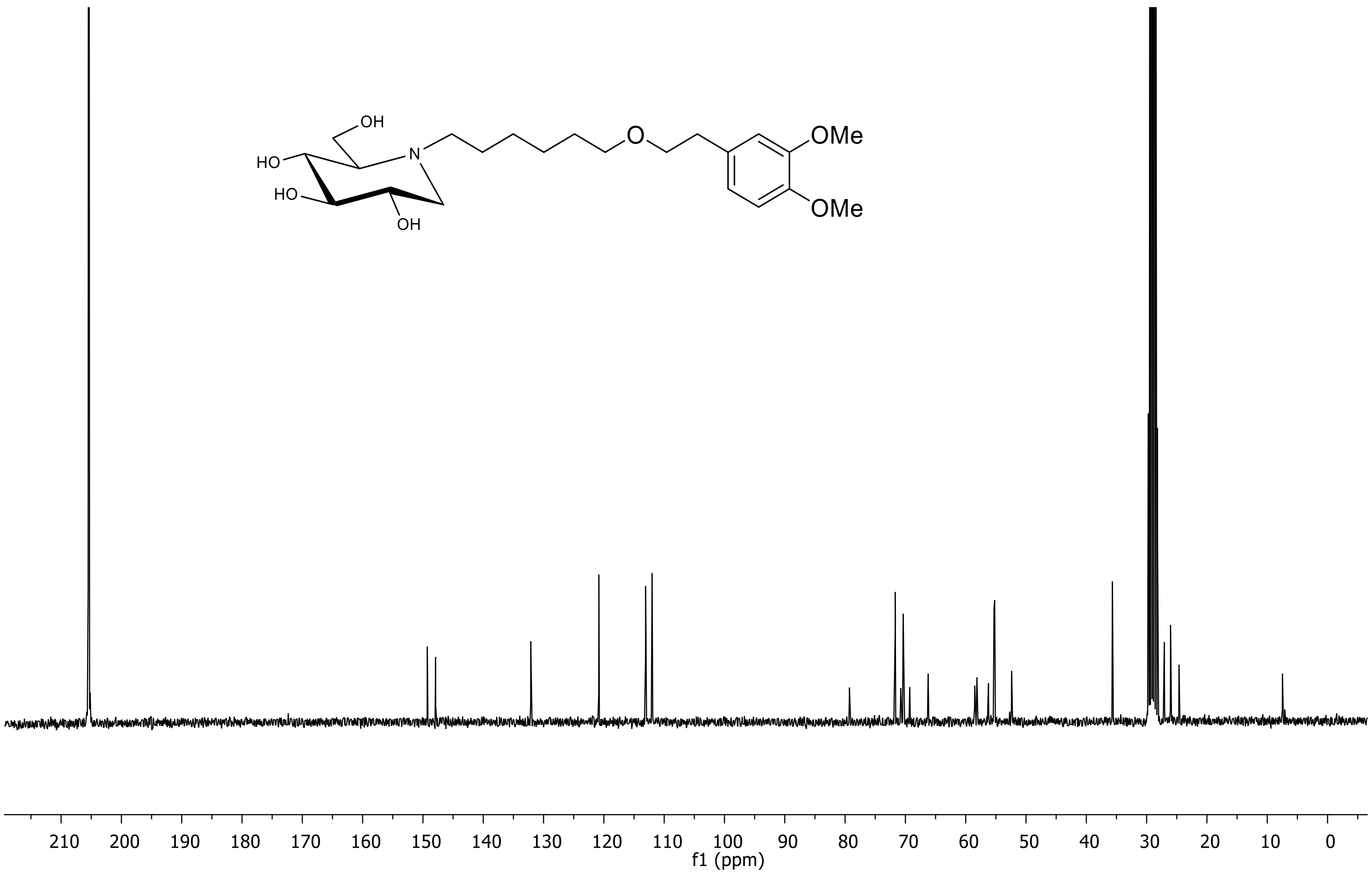

^{13}C-NMR (75.5 MHz, $(CD_3)_2CO$) of **11e**

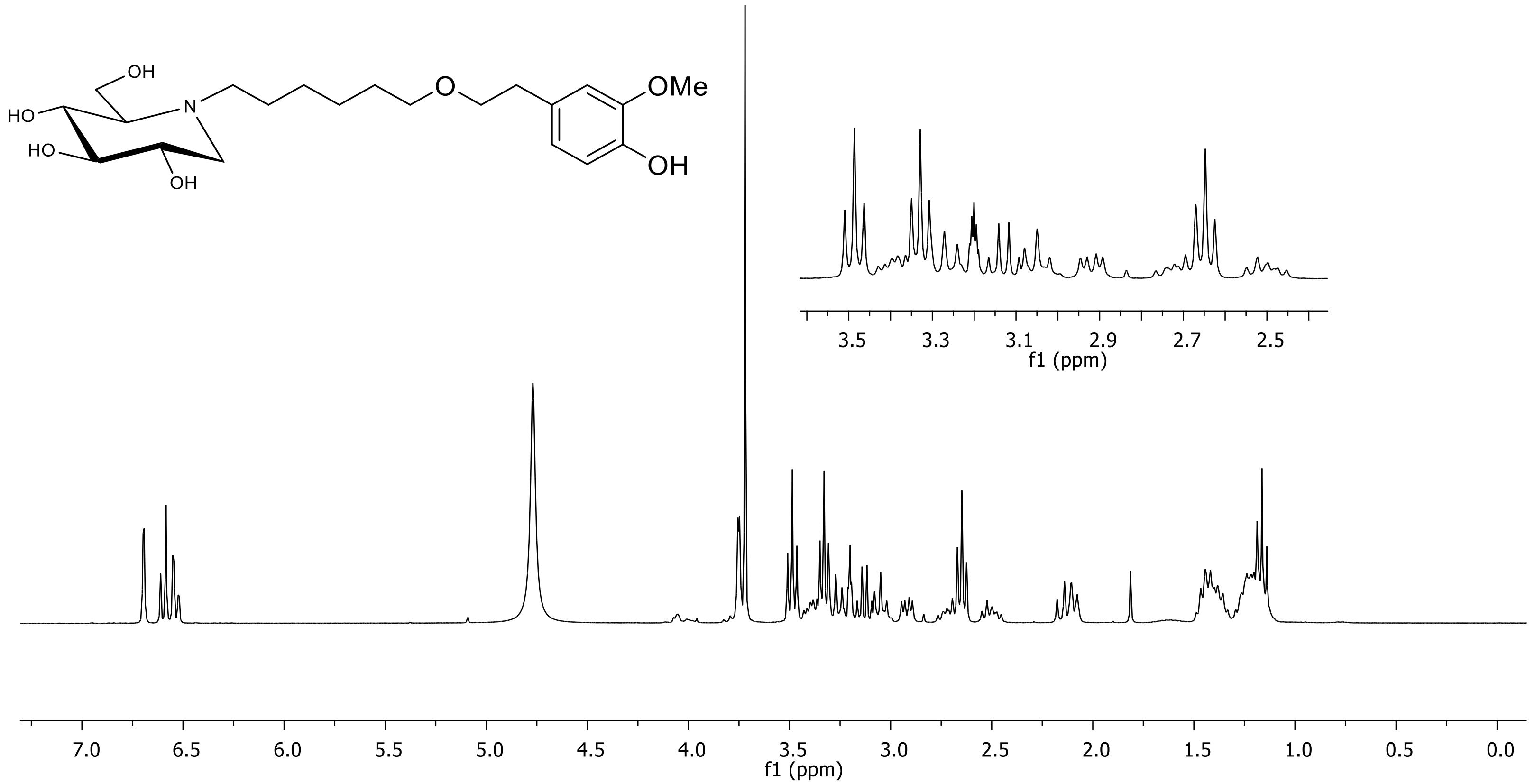

^{1}H-NMR (300 MHz, CD_3OD) of **11f**

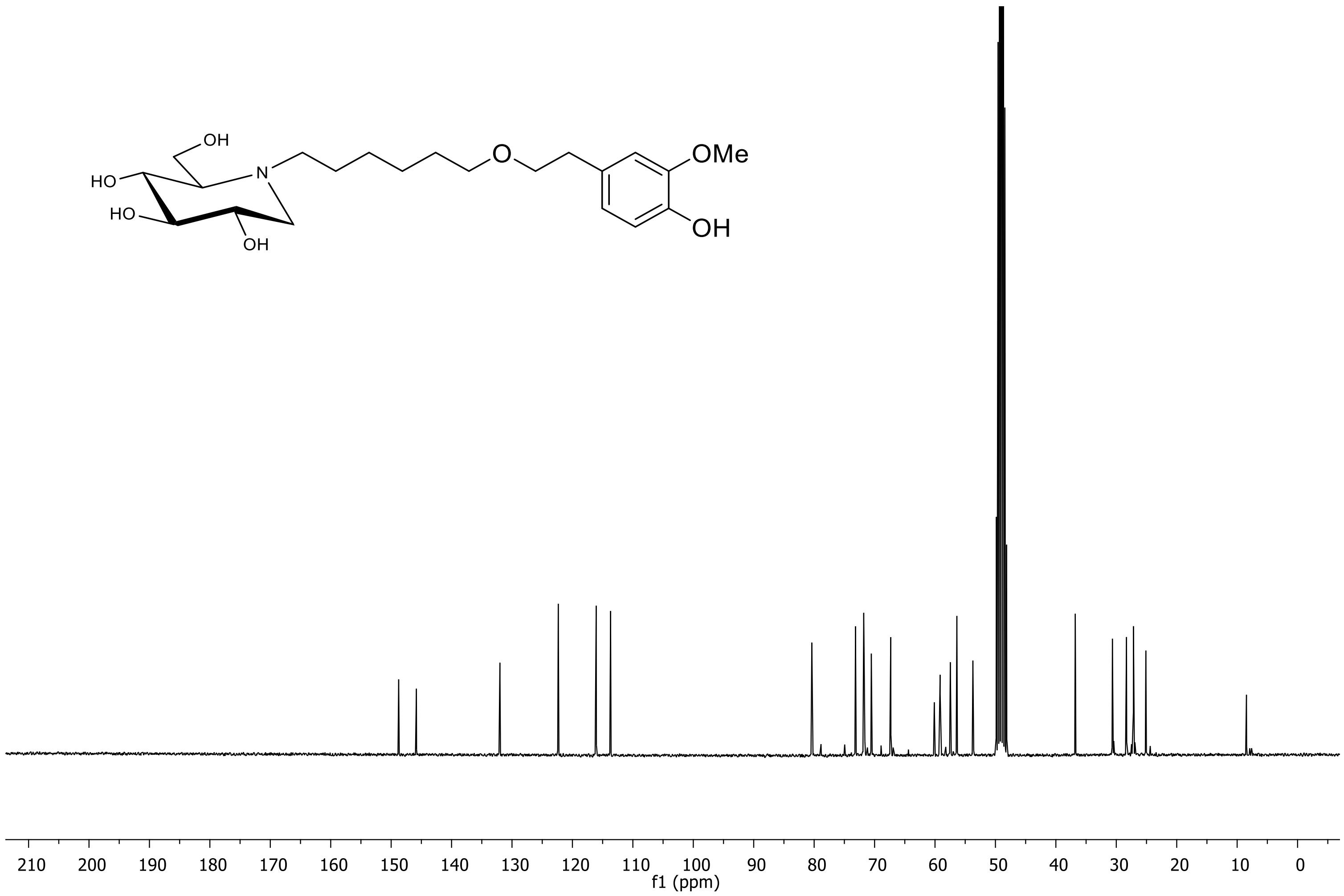

^{13}C-NMR (75.5 MHz, MeOD) of **11f**

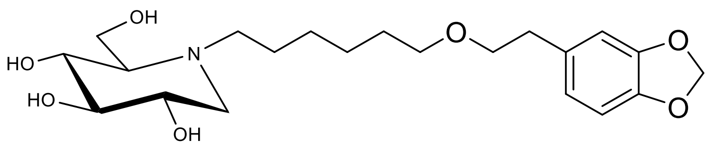

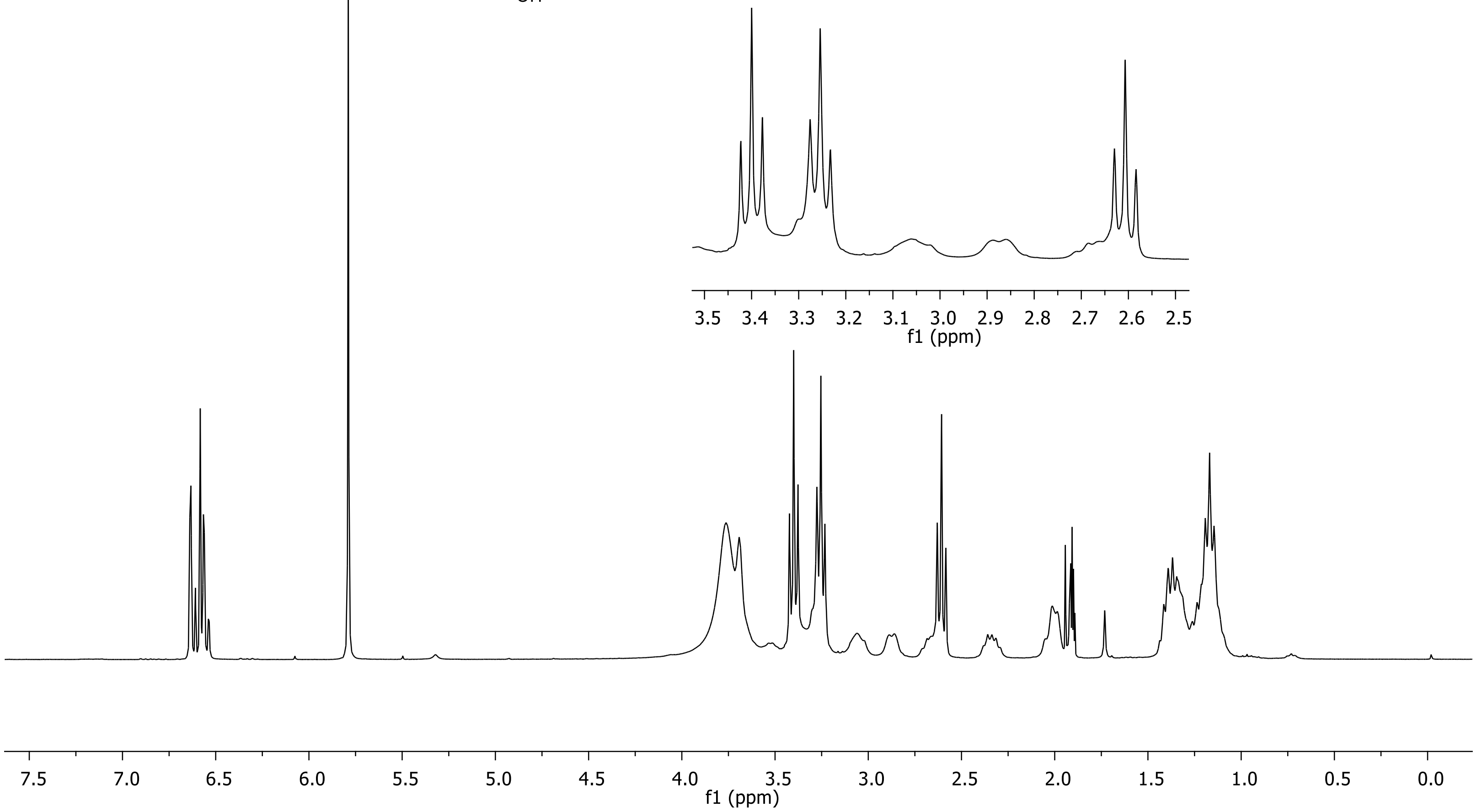

^{1}H-NMR (300 MHz, $(CD_3)_2CO$) of **11g**

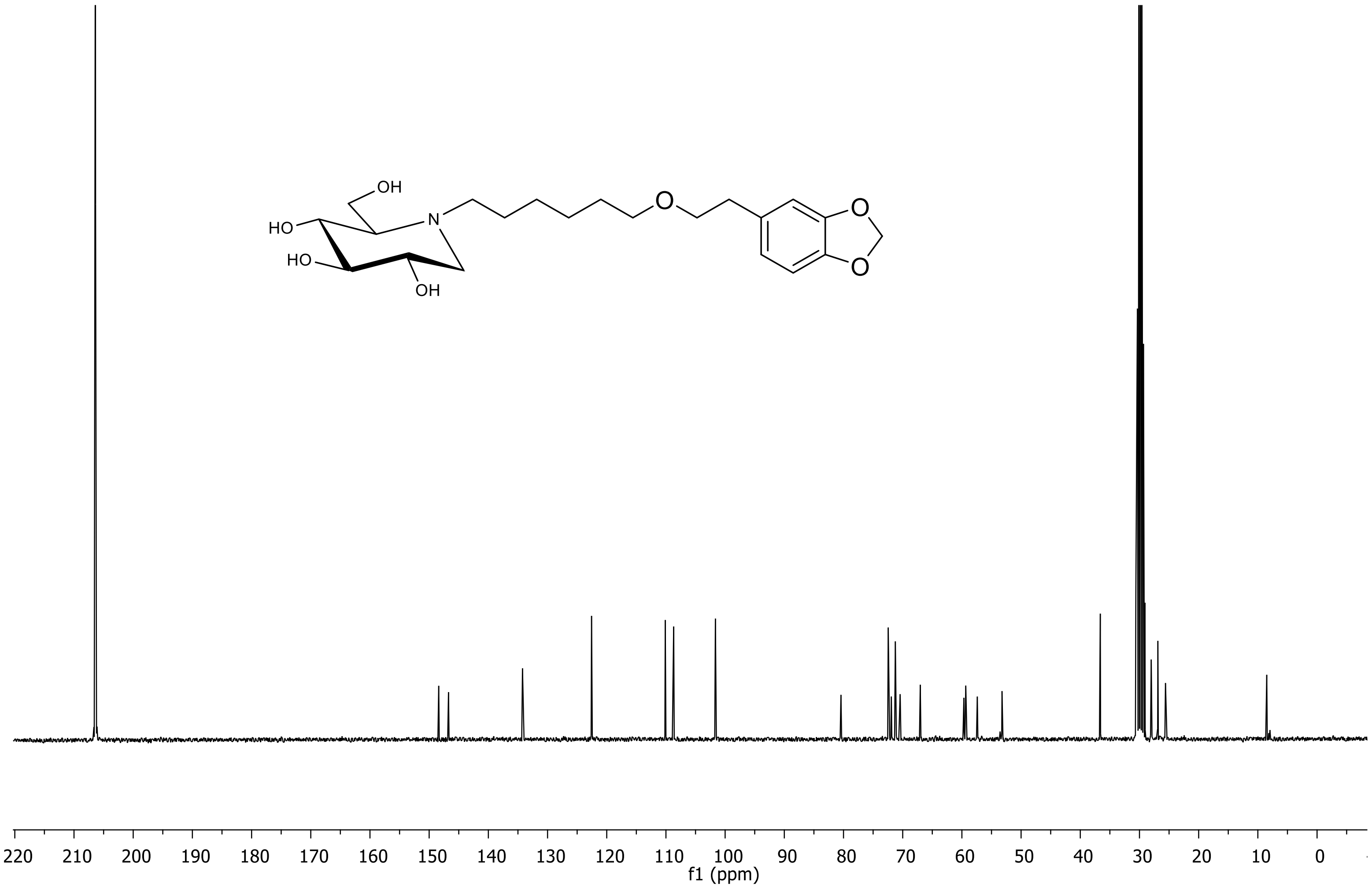

^{13}C-NMR (75.5 MHz, $(CD_3)_2CO$) of **11g**

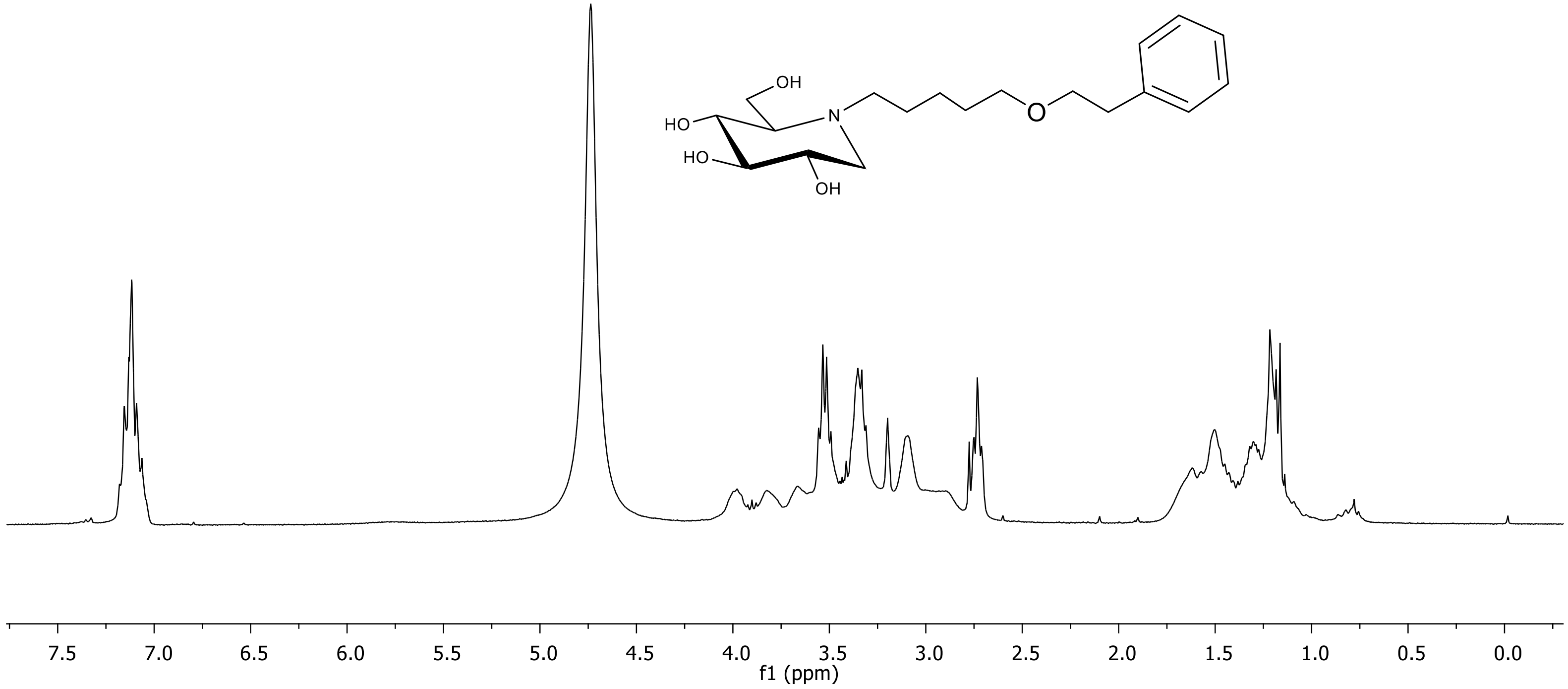

^{1}H-NMR (300 MHz, CD_3OD) of **12a**

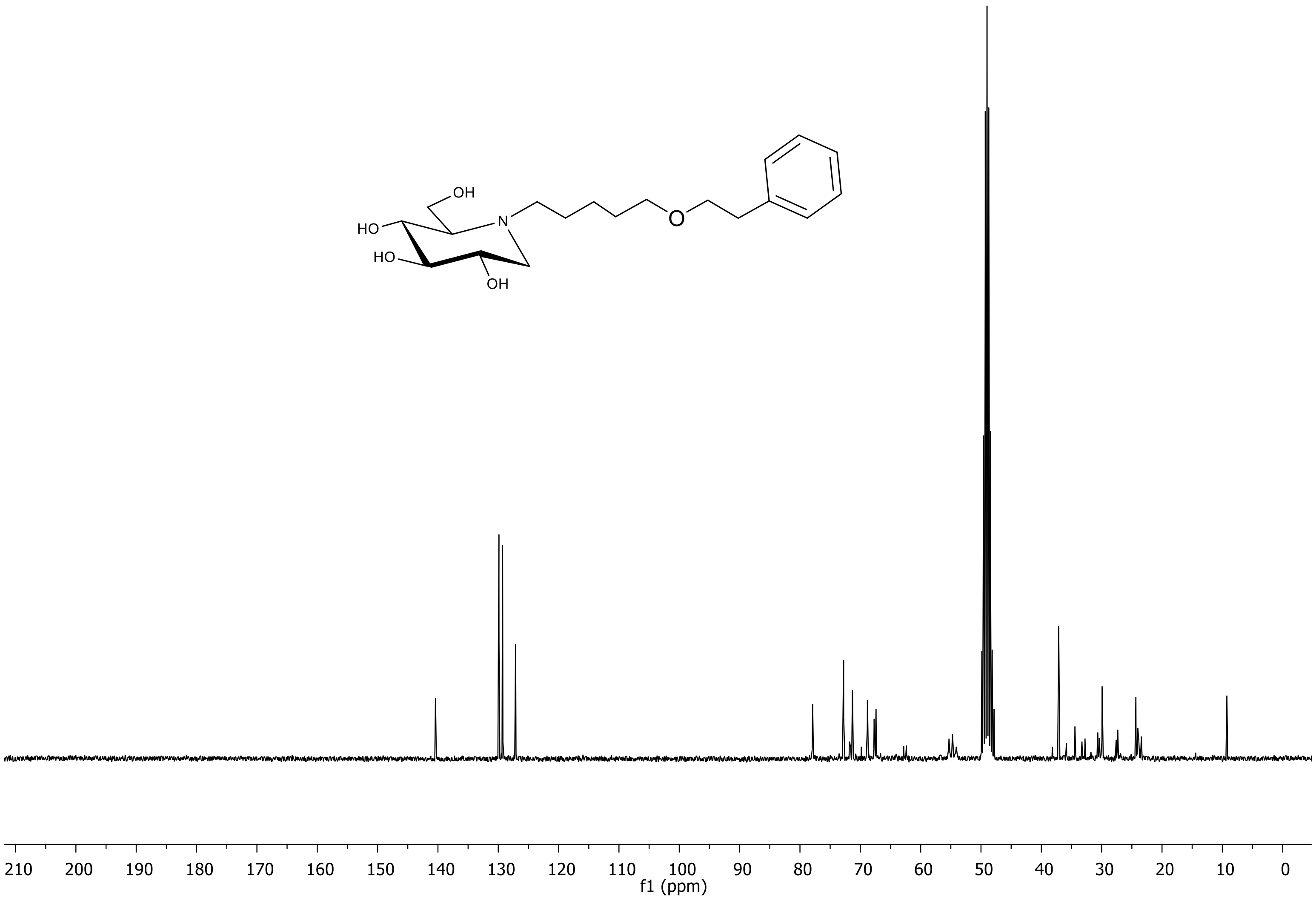

^{13}C-NMR (75.5 MHz, CD_3OD) of **12a**

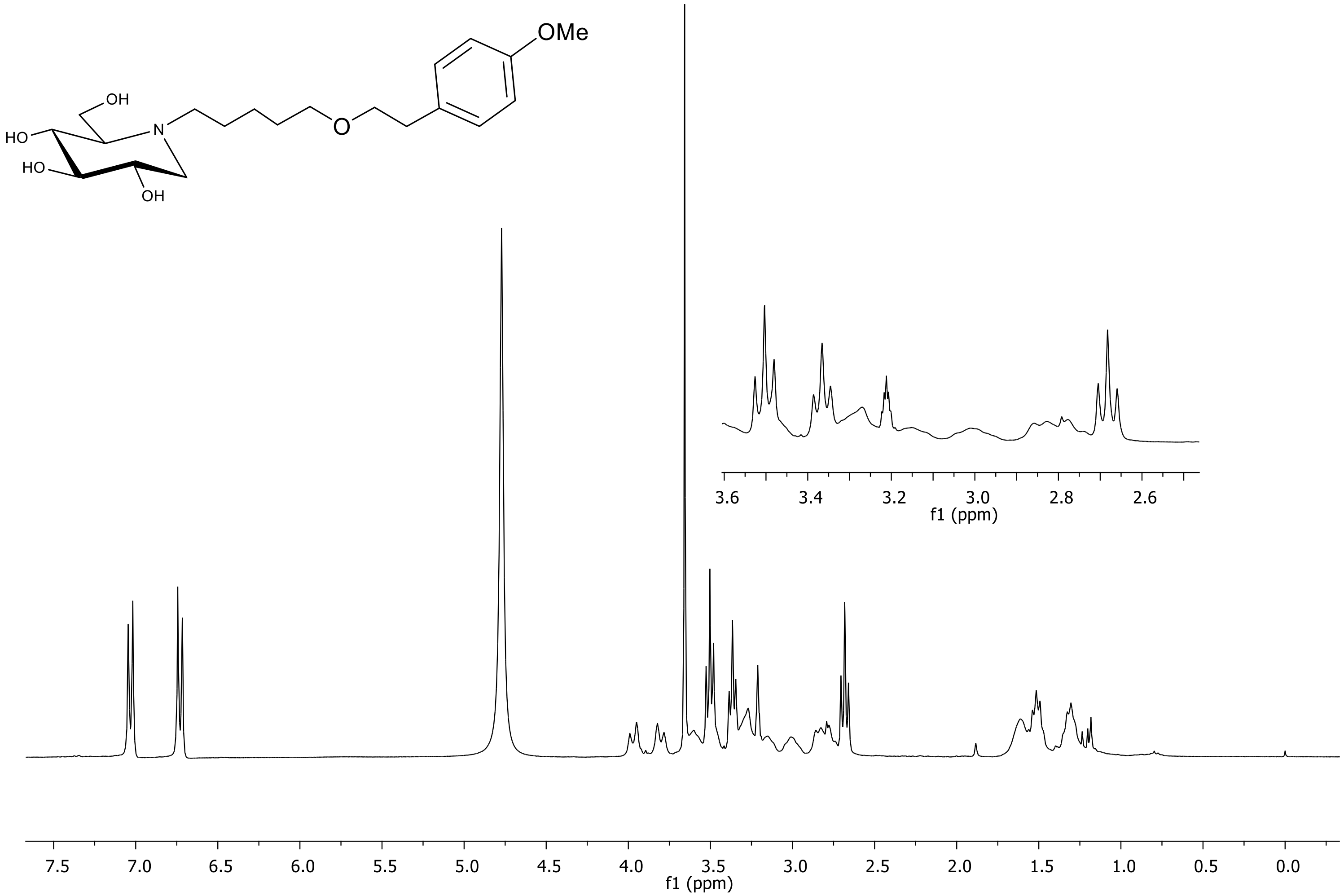

^{1}H-NMR (300 MHz, MeOD) of **12c**

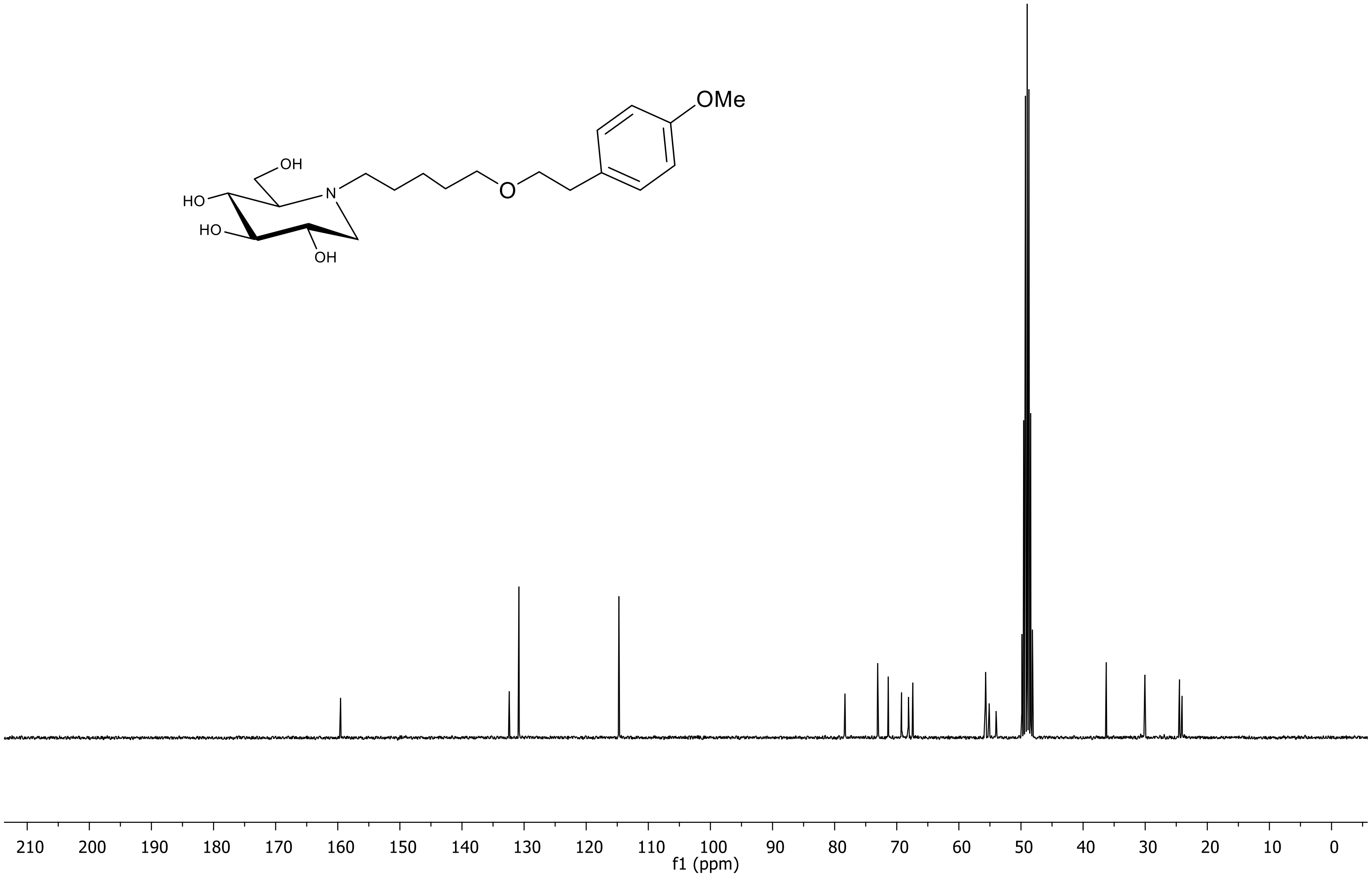

^{13}C-NMR (75.5 MHz, CD_3OD) of **12c**

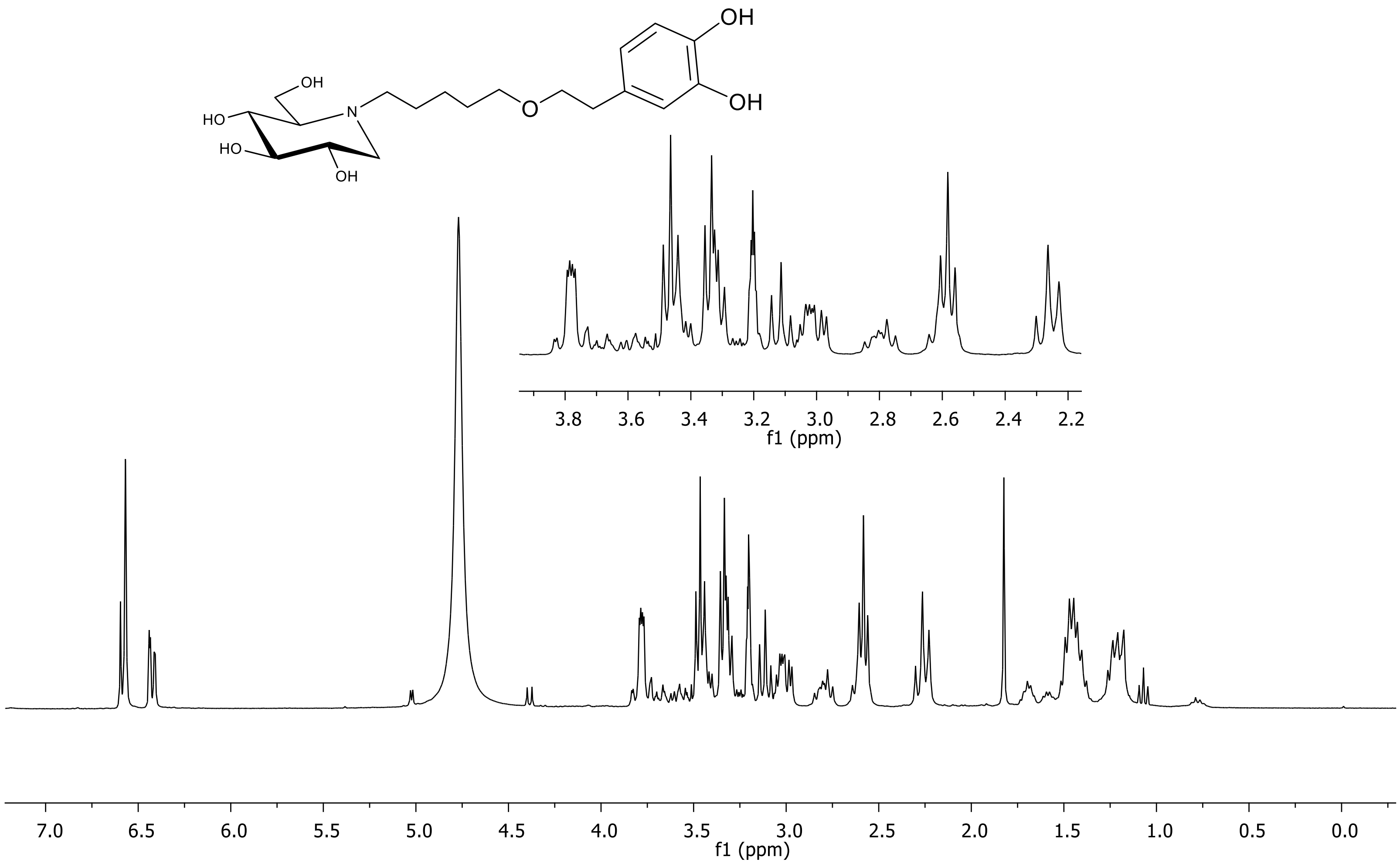

^{1}H-NMR (300 MHz, CD_3OD) of **12d**
